# Global Landscape and Thematic Evolution of Selective GLP‐1 Receptor Agonists and GLP‐1–Based Multi‐Receptor Agonists in Type 2 Diabetes Mellitus: A Bibliometric Analysis

**DOI:** 10.1002/edm2.70351

**Published:** 2026-09-30

**Authors:** Yanbing Wang, Zhanru Liu, Shifeng Wang, Cuiyan Lv, Hesham R. El‐Seedi, Shaden A. M. Khalifa, Haiyan Wang

**Affiliations:** ^1^ Health Management Program Boston University Boston Massachusetts USA; ^2^ School of Traditional Chinese Medicine Beijing University of Chinese Medicine Beijing China; ^3^ School of Chinese Materia Medica Beijing University of Chinese Medicine Beijing China; ^4^ Beijing Hospital of Traditional Chinese Medicine Capital Medical University Beijing China; ^5^ Department of Chemistry, Faculty of Science Islamic University of Madinah Madinah Saudi Arabia; ^6^ Neurology and Psychiatry Department Capio Saint Göran's Hospital Stockholm Sweden

**Keywords:** bibliometric analysis, cardiovascular outcome trials, glucagon‐like peptide‐1 receptor agonist, glucagon‐like peptide‐1–based multi‐receptor agonists, real‐world evidence, Type 2 diabetes mellitus

## Abstract

**Aim:**

To characterize the global development, collaboration structure, publication landscape, citation influence and thematic evolution of research on selective GLP‐1 receptor agonists and GLP‐1–based multi‐receptor agonists in T2DM.

**Methods:**

A bibliometric analysis was conducted on 3736 original articles published between 2004 and 2025 and retrieved from the Web of Science Core Collection. Publication trends, research contributions, collaboration networks, citation impact and thematic evolution were evaluated using complementary bibliometric indicators and network analysis.

**Results:**

Annual publication output increased substantially. Selective‐only studies remained the cumulative core of the evidence base, while mixed/comparative and multi‐receptor‐only studies expanded rapidly in recent years. The United States led in research output and international connectivity, and Novo Nordisk and Eli Lilly were the most prominent institutional contributors based on author affiliation analysis. Diabetes, Obesity and Metabolism was the leading source journal. Research output was highly concentrated across countries but more widely distributed across institutions and authors. Thematic emphasis shifted from incretin biology and glycemic efficacy toward cardiovascular and kidney outcomes, obesity, integrated treatment strategies and real‐world evidence. Major trials, particularly LEADER and REWIND, and ADA/EASD consensus statements exerted strong citation influence. Sensitivity analysis supported the overall robustness of the principal findings.

**Conclusions:**

Research on GLP‐1–based agonists in T2DM has expanded rapidly and shifted toward an integrated cardiovascular–kidney–metabolic framework. Studies of selective GLP‐1RAs remain central to the literature, while studies of newer GLP‐1–based multi‐receptor agonists are contributing to its diversification. Evidence remains comparatively less developed for longer‐term outcomes of emerging agents, treatment sustainability in routine care, patient experience and diverse populations and settings.

## Graphical abstract

Bibliometric analysis of 3736 publications (2004–2025) reveals rapid growth in GLP‐1–based T2DM research. The US leads, with Novo Nordisk and Eli Lilly as top contributors. Thematic shifts from glycemic control toward cardiovascular‐kidney‐metabolic outcomes and real‐world evidence, driven by landmark trials like LEADER and REWIND.

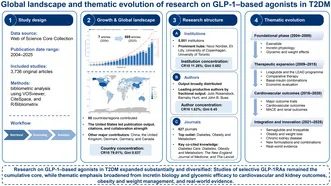

## 1 Introduction

Type 2 diabetes mellitus (T2DM) represents one of the most consequential chronic diseases facing contemporary health‐care systems. In 2024, an estimated 588.7 million adults worldwide were living with diabetes, with T2DM accounting for over 90% of cases, and prevalence is projected to rise to 852.5 million by 2050 [1]. As a progressive multisystem disorder, T2DM, beyond chronic hyperglycemia, markedly increases the risk of atherosclerotic cardiovascular disease, heart failure, chronic kidney disease, retinopathy, neuropathy and premature mortality [2]. Cardiovascular disease was reported in 34.8% T2DM patients in a 13‐country study [3], while diabetic kidney disease affects up to 35% of patients and remains a leading cause of end‐stage renal disease globally [4]. The frequent coexistence of T2DM with obesity further accelerates disease progression and complicates long‐term management [5].

These clinical realities have reshaped therapeutic priorities in diabetes care. Contemporary treatment strategies increasingly emphasize durable metabolic control [6], reduction of cardiovascular and renal risk [7, 8], weight management [9] and long‐term treatment sustainability [10], rather than short‐term glycemic targets alone. Glucose‐lowering therapies are now evaluated not only for their effects on HbA1c but also for their ability to modify hard clinical outcomes and integrate into real‐world care pathways [11]. Against this background, selective GLP‐1 receptor agonists have become established components of T2DM management [12], acting through glucose‐dependent stimulation of insulin secretion, delayed gastric emptying and reduced appetite [13]. More recently, GLP‐1–based multi‐receptor agonists have extended this therapeutic approach by engaging additional metabolic hormone receptors.

In this study, selective GLP‐1 receptor agonists and GLP‐1–based multi‐receptor agonists are collectively referred to as GLP‐1–based agonists. Although the literature on these agents has expanded rapidly, a comprehensive bibliometric assessment of the field's development, collaboration structure, citation influence and thematic evolution remains lacking. We therefore mapped these dimensions to contextualize the growth and diversification of the literature and to identify areas that remained comparatively less developed.

## 2 Methods

### 2.1 Data Source and Study Identification

The Web of Science Core Collection (WoSCC) was used as the data source for the bibliometric analysis. WoSCC was selected because it provides standardized citation metadata and stable longitudinal coverage, which are essential for mapping the development and influence of clinical evidence over time in diabetes research. An independent PubMed search was conducted as a cross‐database sensitivity analysis.

The search strategy targeted original research on GLP‐1–based agonists in T2DM and combined drug‐class terms, generic and brand names of individual agents, and disease‐specific terms, with proximity operators used to improve specificity. The exact WoSCC search string is provided in File S1.

The search covered the period from 1 January 2004 to 31 December 2025. Only original research articles published in English were included. The WoSCC search and citation‐count update were completed on July 26, 2026.

All retrieved records were screened independently by two investigators based on titles, abstracts and, when required, full texts. Screening was conducted according to a predefined eligibility assessment manual, which specifies the inclusion and exclusion criteria, hierarchical exclusion rules, screening procedures and approaches for resolving uncertain cases. The complete screening manual is provided as File S2. The detailed study identification, screening and inclusion process is presented in Figure 1.

**FIGURE 1:**
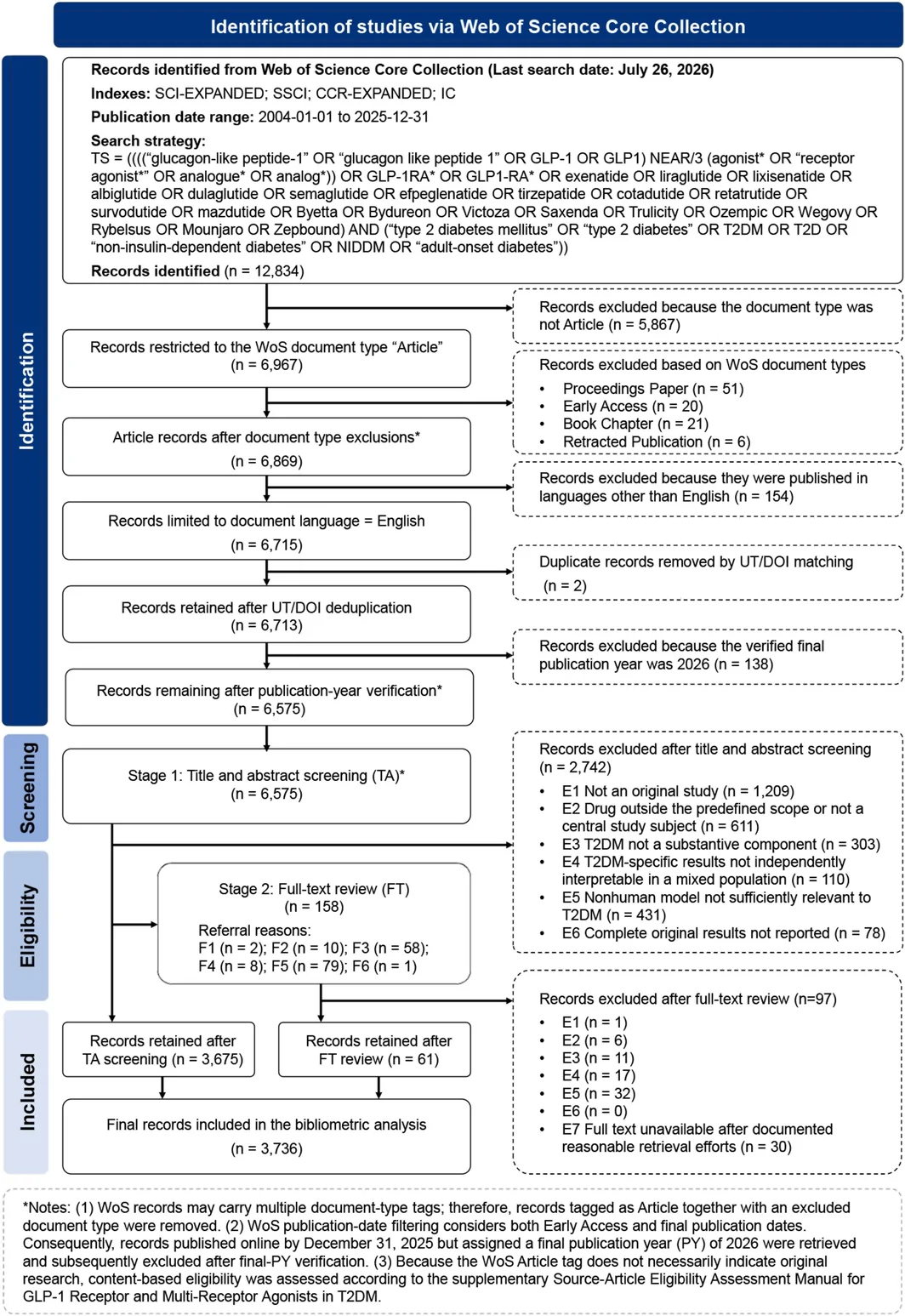
Study identification and selection flow diagram for the bibliometric analysis of research on GLP‐1–based agonists in T2DM, 2004–2025.

### 2.2 Data Cleaning and Standardization

Full bibliographic records were exported from WoSCC and de‐duplicated by matching WoS accession numbers and normalized DOIs. Country and institutional affiliations were standardized for abbreviations, spelling, punctuation and synonymous names; author names were retained at the highest resolution available. Within‐record duplicate entities were removed, and records lacking valid entity information were excluded only from the affected analysis.

Keyword standardization was performed to enhance the clinical interpretability of thematic analyses. Textual variations arising from capitalization, punctuation, spelling (American vs British) and singular/plural forms were harmonized. A predefined synonym mapping table was then applied to consolidate abbreviations, synonymous terms and drug‐related expressions.

Since administration route and dosing frequency are clinically salient features of therapy with GLP‐1–based agonists, two complementary standardization strategies were applied, depending on analytic purpose. For keyword co‐occurrence networks, trend analyses and frequency‐normalized heatmaps, drug‐level standardization was used, whereby different formulations of the same agent were merged under the generic drug name to reflect overall clinical attention. In contrast, for keyword timeline analyses and burst detection, regimen‐level terms (e.g., oral semaglutide, once‐weekly semaglutide) were retained to capture the temporal evolution of delivery strategies and treatment innovation. The corresponding drug‐level and regimen‐level standardization dictionaries are provided in Files S3 and S4, respectively.

### 2.3 Bibliometric Analysis and Visualization

VOSviewer (version 1.6.20) was used to construct co‐authorship networks for countries/regions, institutions and authors; journal citation and co‐citation networks; and keyword co‐occurrence networks. Total link strength (TLS) was defined as the sum of the strengths of all links incident to a node. Specific parameter settings are provided in Table S1.

CiteSpace (version 6.4.R1) was used for journal dual‐map overlays, keyword timelines and keyword and reference burst detection.

R (version 4.5.2) was used for record processing, descriptive and concentration analyses, citation normalization, sensitivity analyses and visualizations. Bibliometrix (version 5.2.1) was used to derive corresponding‐author single‐country publication (SCP) and multiple‐country publication (MCP) indicators.

Countries and author institution affiliations were parsed from the WoS author‐address field (C1), and authors from the full‐author‐name field (AF). Full counting assigned one credit to each distinct participating entity, whereas fractional counting divided one credit equally among the k distinct entities on each article. Country collaboration was summarized using SCP, MCP and the MCP proportion; output concentration was summarized using CR10, the Herfindahl–Hirschman index, the Gini coefficient and Lorenz curves. Dataset‐specific author H‐indices were calculated from citation counts of included articles linked through the AF field; self‐citations were retained. Institutional affiliation was determined solely from the author‐address information (C1) and was analysed independently of article‐level funding or sponsorship; consequently, industry affiliation was not treated as a proxy for funding involvement or conflict of interest, which were not analysed in the present bibliometric study.

Subject‐category assignments and counts for the included articles were obtained from WoSCC. 2025 Journal Impact Factors (JIF) and quartiles were obtained from the 2026 release of Journal Citation Reports (JCR).

### 2.4 Sensitivity and Robustness Analyses

Robustness analyses addressed cross‐database coverage, network inclusion thresholds, dataset scope and citation normalization. An independent PubMed search assessed cross‐database coverage of highly cited WoSCC articles (Table S2). Network inclusion thresholds were varied to 0.8, 1.0, and 1.2 times the primary threshold (Table S3). Robustness to dataset scope was evaluated across three progressively expanded analytical scopes: Dataset 1 (selective‐only), Datasets 1 + 2 (selective‐only plus mixed/comparative) and Datasets 1 + 2 + 3 (the full dataset, additionally including multi‐receptor‐only studies), using Top‐10 overlap, Spearman's rank correlation, the adjusted Rand index and normalized mutual information (File S5).

Citation impact was normalized within publication year by dividing each article's total citations by the annual mean. Annual citation distributions were summarized using the mean, median and interquartile range and visualized on a log1p‐transformed scale (Figure S1). The primary normalized ranking covered 2004–2024, whereas the citation‐immature 2025 cohort was ranked separately by total citations (Table S4). Sensitivity to the normalization method was evaluated by comparing the mean‐based ranking with within‐year Hazen percentile rankings using Top‐10 overlap and Spearman's rank correlation (Table S5).

Detailed equations, analytic procedures and corresponding scripts are provided in the Supporting Information S1 and Code package.

## 3 Results

### 3.1 Global Publication Output and Temporal Growth

A total of 3736 original research articles on GLP‐1–based agonists in T2DM were published between 2004 and 2025. Annual output increased from 7 articles in 2004 to 589 in 2025 (Figure 2). Selective‐only studies (Dataset 1) remained predominant (3544; 94.86%), whereas mixed/comparative studies (Dataset 2) and multi‐receptor‐only studies (Dataset 3) first appeared in 2020 and increased to 43 and 40 articles, respectively, in 2025, together accounting for 14.09% of that year's output. PubMed sensitivity analysis showed over 97% coverage of the top 100, 200 and 300 highly cited WoS articles (Table S2).

**FIGURE 2:**
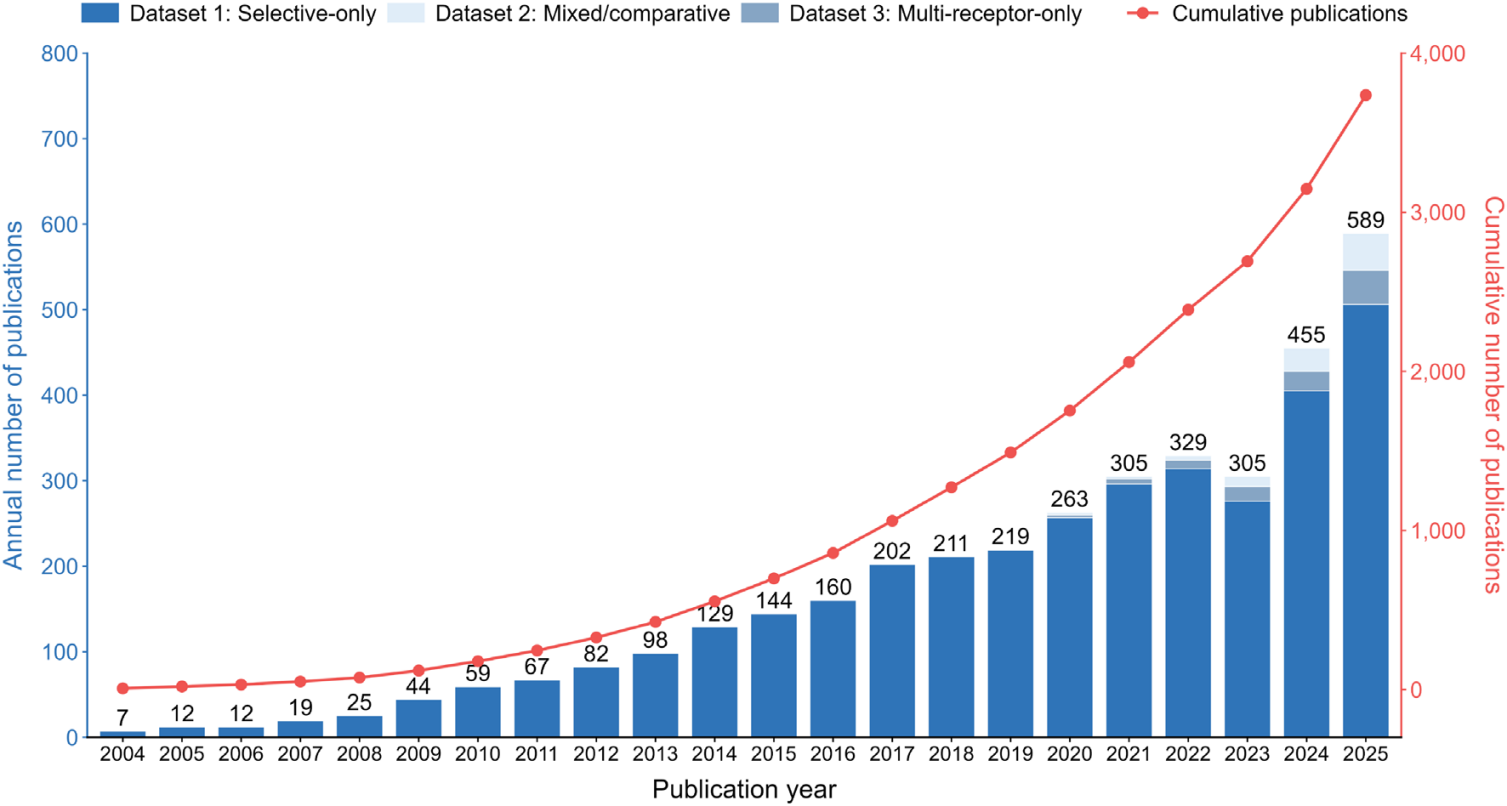
Annual publication trend in research on GLP‐1–based agonists in T2DM, 2004–2025. The stacked bars show the annual numbers of original research articles in three mutually exclusive datasets: Dataset 1 (selective‐only), comprising studies restricted to selective GLP‐1RAs; Dataset 2 (mixed/comparative), comprising studies involving both selective GLP‐1RAs and GLP‐1–based multi‐receptor agonists; and Dataset 3 (multi‐receptor‐only), comprising studies restricted to GLP‐1–based multi‐receptor agonists. Numbers above the bars indicate the total annual output across the three datasets. The line shows the cumulative number of publications across all datasets.

The distribution of publications across Web of Science subject categories is shown in Figure 3. Endocrinology & Metabolism dominates (55.03%), followed by Pharmacology & Pharmacy (13.57%) and Medicine, General & Internal (11.22%). Medicine, Research & Experimental (6.18%) and Cardiac & Cardiovascular Systems (5.65%) also contributed appreciably, showing that the literature extended across multiple biomedical and clinical disciplines.

**FIGURE 3:**
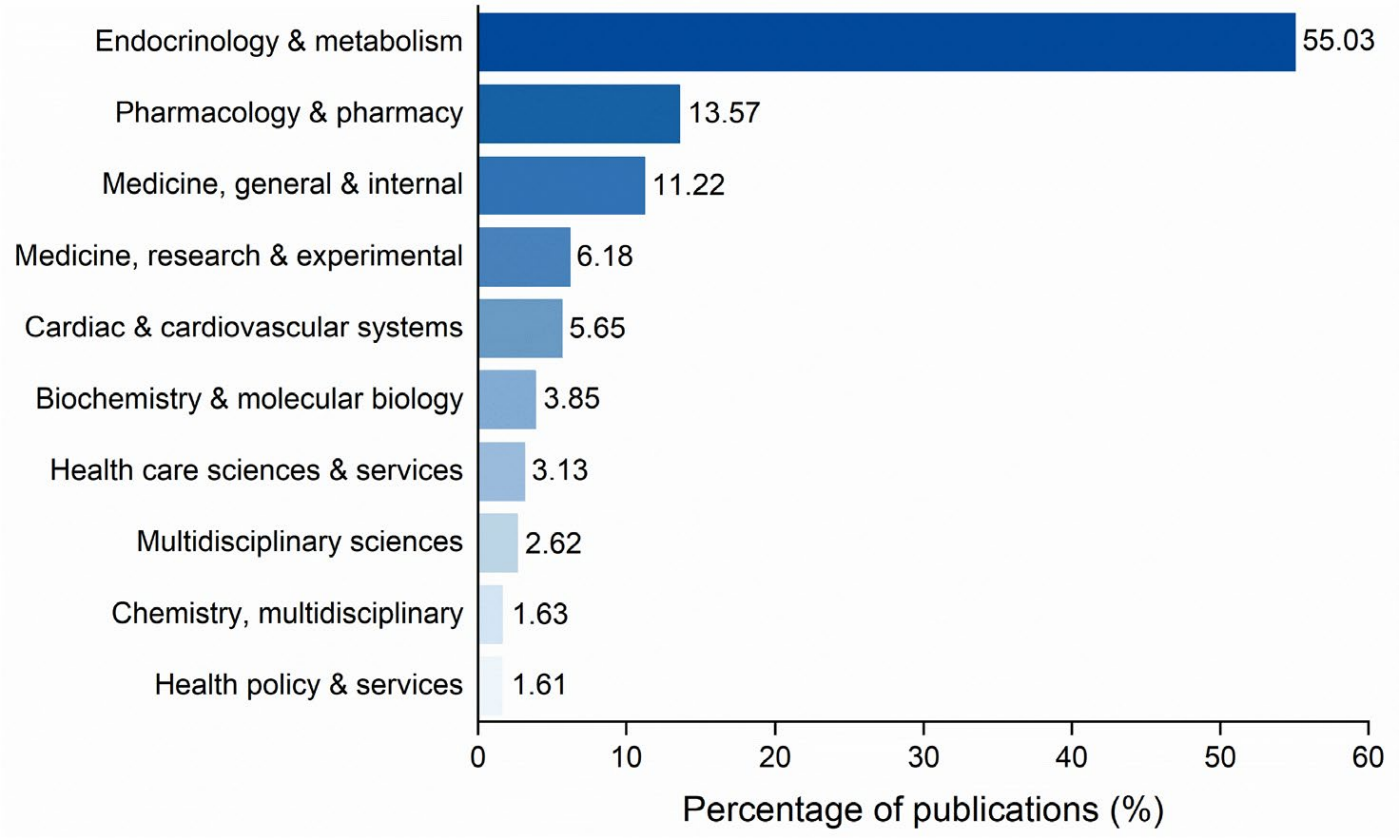
Research on GLP‐1–based agonists in T2DM across Web of Science subject categories, 2004–2025. The horizontal bars show the percentages of the 3736 included articles assigned to the ten most represented Web of Science subject categories. Because an article may be assigned to more than one category, the percentages are not mutually exclusive and do not sum to 100%.

### 3.2 Geographic Distribution and International Collaboration

#### 3.2.1 Geographic Distribution and Country‐Level Research Performance

A total of 80 countries/regions contributed to research on GLP‐1–based agonists in T2DM. Research activity was geographically widespread but concentrated primarily in North America, Western Europe and East Asia, whereas contributions from most countries in Africa, Central Asia and parts of South America remained comparatively limited (Figure 4a).

**FIGURE 4:**
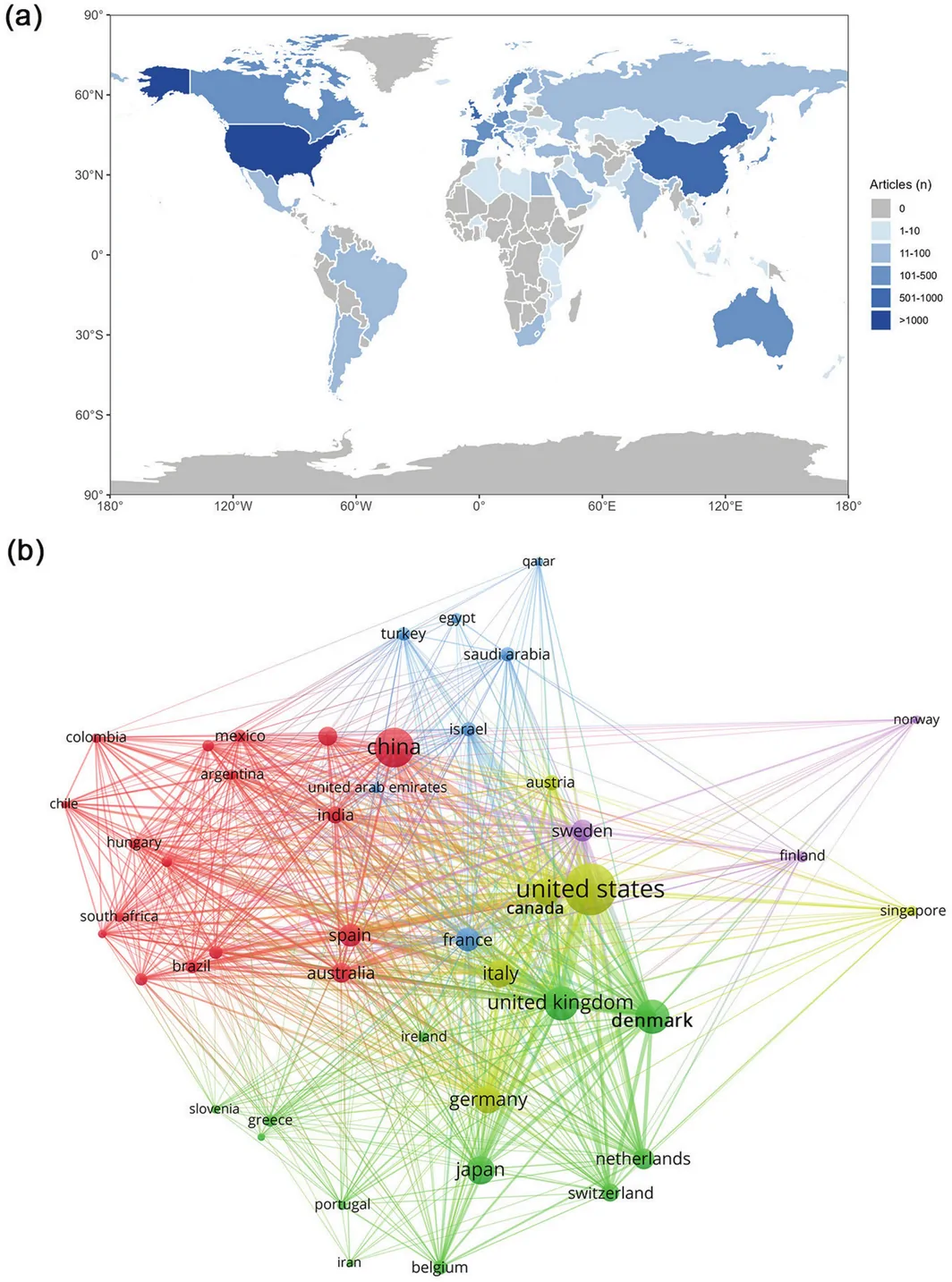
Geographic distribution and international co‐authorship collaboration networks in research on GLP‐1–based agonists in T2DM, 2004–2025. (a) World map showing the geographic distribution of publication output. Darker shades of blue indicate larger numbers of articles, whereas grey indicates no publications. (b) International co‐authorship network map generated using VOSviewer. The network included 45 countries/regions with at least 10 publications. Node size represents publication output, node colour indicates network cluster membership and link thickness represents the strength of bilateral co‐authorship.

Under full counting, the United States ranked first in publication output, total citations and total collaboration strength, with 1558 articles, 112,863 citations and a TLS of 2378, respectively (Figure 5, Table 1). China ranked second in publication output with 773 articles, followed by the United Kingdom with 543 and Denmark with 537. However, rankings differed across indicators. The United Kingdom and Denmark ranked second and third in total citations, with 52,474 and 51,961 citations, respectively, whereas China ranked tenth in TLS (532) despite its comparatively high publication output. The United Kingdom and Denmark also received more citations per publication than the United States, averaging 96.6, 96.8 and 72.4 citations per article, respectively.

**FIGURE 5:**
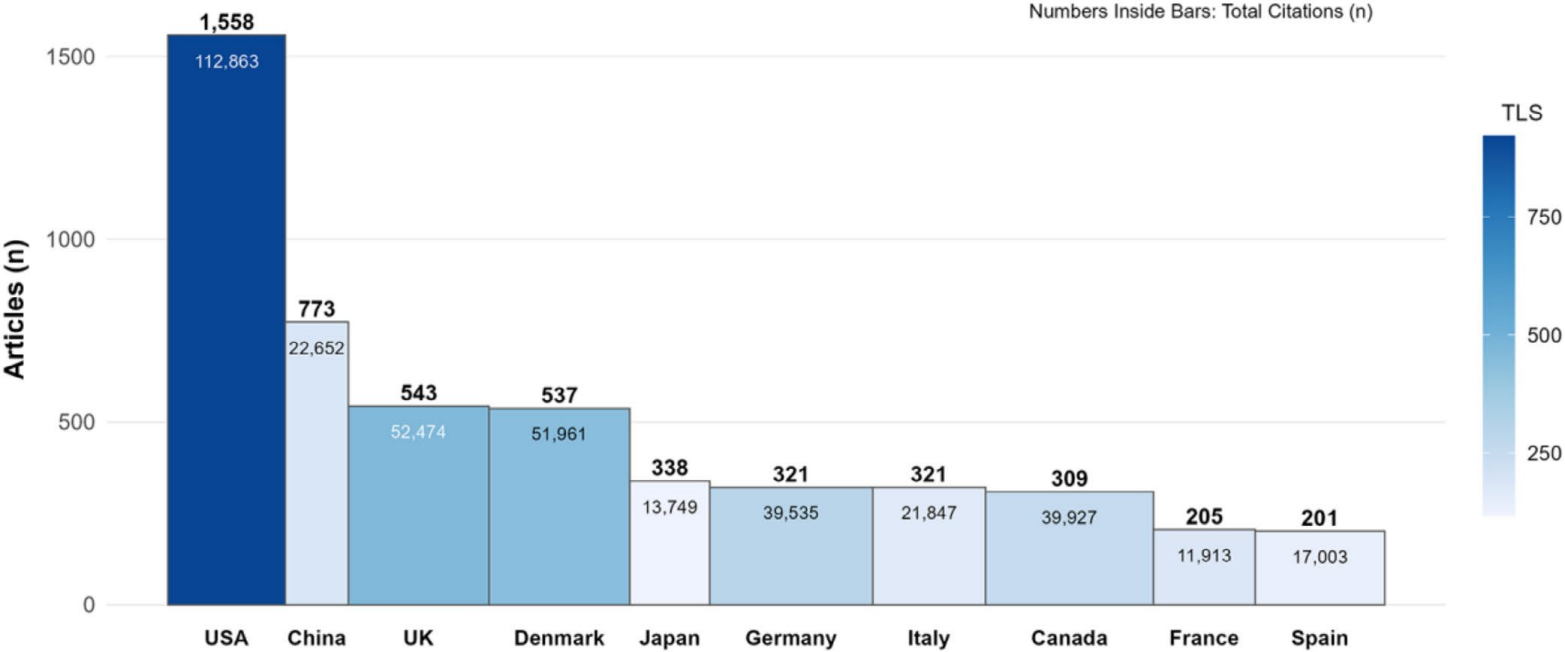
Publication output, citation impact and collaboration strength of the 10 most productive countries in research on GLP‐1–based agonists in T2DM, 2004–2025. Bar height and the numbers above the bars represent the full‐counted number of articles attributed to each country. Numbers inside the bars indicate total citations, and colour intensity represents TLS.

**TABLE 1:** Top 10 countries in research on GLP‐1–based agonists in T2DM, 2004–2025, ranked by TLS.

Rank	Country	Degree centrality	Normalized degree centrality	TLS
1	United States	44	1.000	2378
2	United Kingdom	44	1.000	1453
3	Denmark	41	0.932	1200
4	Germany	41	0.932	1025
5	Canada	40	0.909	939
6	Spain	40	0.909	643
7	France	42	0.955	634
8	Sweden	41	0.932	606
9	Italy	41	0.932	600
10	China	39	0.886	532

*Note:* The network included 45 countries/regions with at least 10 publications. Degree centrality represents the number of direct collaborative links of a country/region. Normalized degree centrality was calculated as $\text{degree}/\left(N-1\right)$, where *N* = 45. TLS represents the sum of the strengths of all collaborative links of a country/region.

#### 3.2.2 Concentration and Structure of International Collaboration

After multinational publications were fractionally allocated among participating countries, country‐level research output remained highly concentrated. The 10 most productive countries collectively contributed 79.01% of the total fractional publication output, while the United States and China alone accounted for 43.11% (25.58% and 17.53%, respectively). The marked deviation of the Lorenz curve from the line of equality, together with a Gini coefficient of 0.837, indicated substantial inequality in the distribution of research output across countries (Figure 6).

**FIGURE 6:**
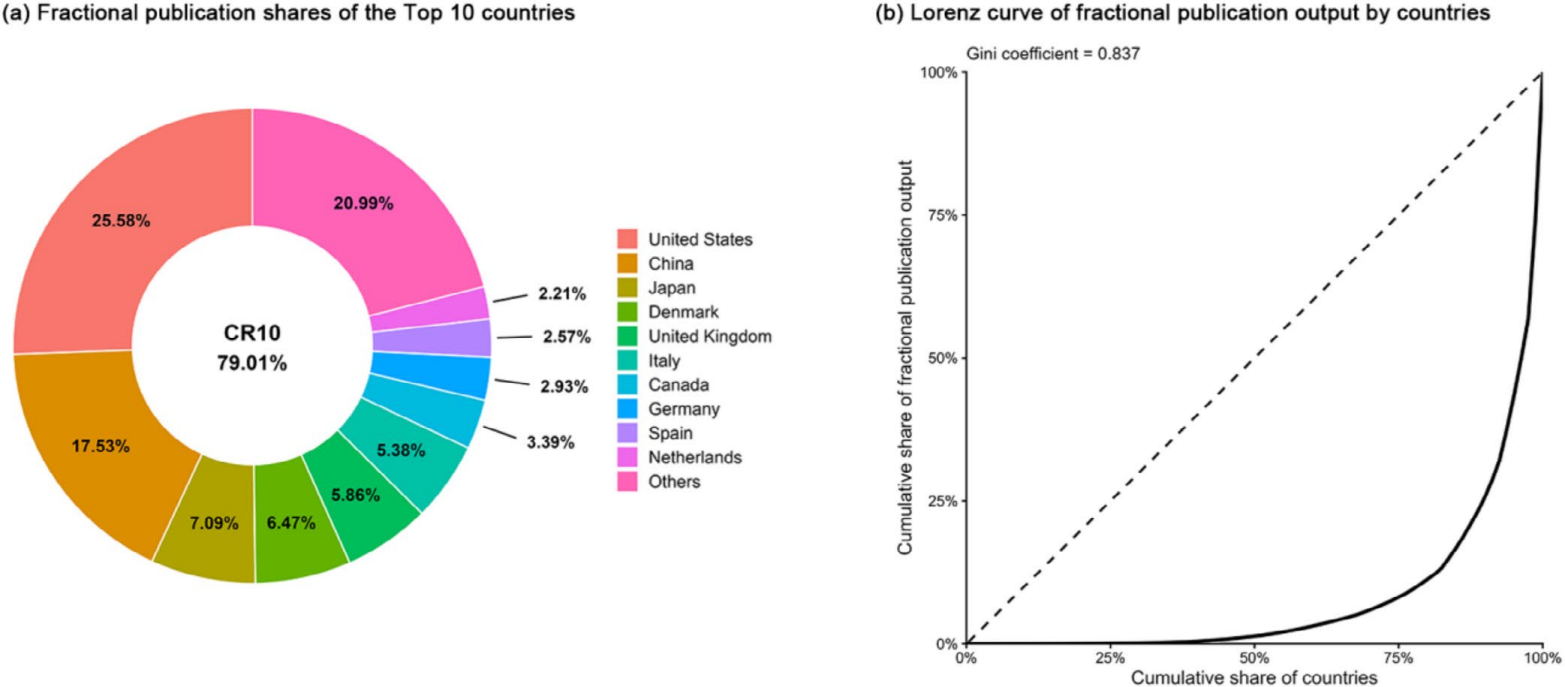
Concentration of country‐level publication output in research on GLP‐1–based agonists in T2DM, 2004–2025. (a) Fractional publication shares of the 10 most productive countries and all remaining countries. CR10 denotes the cumulative share of fractional publication output contributed by the top 10 countries. (b) Lorenz curve of fractional publication output across countries. The dashed diagonal indicates perfect equality.

The international co‐authorship network comprised 45 countries/regions with at least 10 publications (Figure 4b). The United States and the United Kingdom each had a degree centrality of 44 and a normalized degree centrality of 1.000, indicating direct collaboration with all other countries/regions in the network. Despite their identical collaboration breadth, the United States had a substantially higher TLS than the United Kingdom (2378 vs. 1453). Denmark, Germany and Canada also occupied prominent positions in the weighted collaboration network, with TLS values of 1200, 1025 and 939, respectively (Table 1).

Collaboration breadth and strength were not necessarily concordant. France was directly connected to 42 of the other 44 countries/regions (normalized degree centrality = 0.955) but ranked seventh in TLS (634). China also showed broad international connectivity, with a degree centrality of 39 and a normalized degree centrality of 0.886. Conversely, Sweden ranked eighth in TLS (606) despite not being among the 10 largest contributors by fractional publication output.

Corresponding‐author analysis further revealed substantial differences in the relative contribution of international collaboration (Figure 7). The United States and China had the largest corresponding‐author publication outputs, but MCPs accounted for 39.8% and 16.7% of their respective outputs. Germany and the United Kingdom had the highest MCP proportions, at 74.7% and 69.9%, respectively, followed by Canada (60.4%) and the Netherlands (54.5%). In contrast, China, Italy and Japan relied predominantly on SCPs.

**FIGURE 7:**
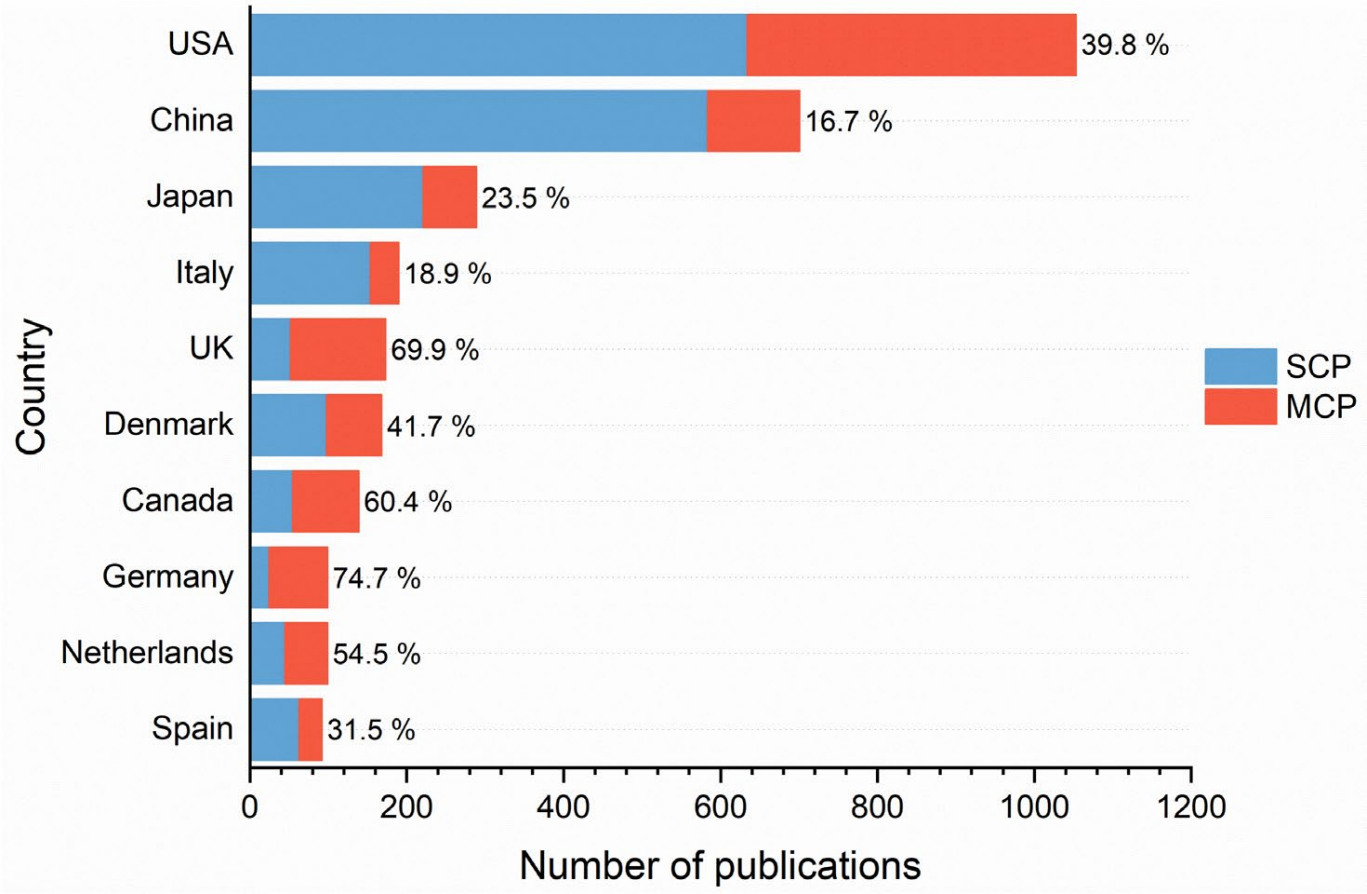
Leading corresponding‐author countries and their international collaboration patterns in research on GLP‐1–based agonists in T2DM, 2004–2025. Stacked bar lengths represent the total numbers of publications attributed to corresponding authors from each country. Publications are classified as single‐country publications (SCPs) or multiple‐country publications (MCPs), with MCPs involving authors from more than one country. Percentages indicate the proportion of MCPs among the corresponding‐author publications attributed to each country.

### 3.3 Institutional Contributions and Collaboration

#### 3.3.1 Institutional Productivity, Citation Impact and Fractional Contribution

A total of 5051 institutions, based on author affiliation, contributed to research on GLP‐1–based agonists in T2DM. Pharmaceutical companies and academic research centers were both prominent among the leading institutions. Novo Nordisk and Eli Lilly ranked first and second in both full‐counted publication output (426 and 343 articles) and total citations (43,640 and 32,211), respectively. Among academic institutions, the University of Toronto ranked third in total citations (26,937), followed by the University of Texas Southwestern Medical Center (24,561), the University of North Carolina (19,191) and the University of Copenhagen (12,468) (Table 2).

**TABLE 2:** Top 10 institutions in research on GLP‐1–based agonists in T2DM, 2004–2025, ranked by total citations.

Rank	Institution	Citations (*n*)	Country
1	Novo Nordisk	43,640	Denmark
2	Eli Lilly	32,211	United States
3	University of Toronto	26,937	Canada
4	University of Texas Southwestern Medical Center	24,561	United States
5	University of North Carolina	19,191	United States
6	University of Copenhagen	12,468	Denmark
7	Amylin Pharmaceuticals	11,864	United States
8	Imperial College London	11,249	United Kingdom
9	University of Washington	11,040	United States
10	Duke University	8010	United States

Under fractional counting, Eli Lilly ranked first with 111.68 fractional documents, followed by Novo Nordisk with 107.78 and the University of Copenhagen with 34.41 (Table 3). Although the order of the two leading institutions was reversed, Eli Lilly and Novo Nordisk remained the top two under both full and fractional counting, indicating that their high publication outputs were not solely attributable to repeated full counting of multi‐institutional articles.

**TABLE 3:** Top 10 institutions in research on GLP‐1–based agonists in T2DM, 2004–2025, ranked by fractionalized publication output.

Rank	Institution	Full documents	Fractional documents	Fractional/Full	Full/Fractional
1	Eli Lilly	343	111.68	0.326	3.07
2	Novo Nordisk	426	107.78	0.253	3.95
3	University of Copenhagen	133	34.41	0.259	3.86
4	Harvard Medical School	118	28.28	0.24	4.17
5	AstraZeneca	88	25.71	0.292	3.42
6	University of Toronto	130	25.15	0.193	5.17
7	Sanofi‐Aventis	115	24.75	0.215	4.65
8	Amylin Pharmaceuticals	62	24.68	0.398	2.51
9	Shanghai Jiao Tong University	42	20.57	0.49	2.04
10	University of Texas Southwestern Medical Center	94	17.16	0.183	5.48

*Note:* Institutions were ranked in descending order of fractional publication output. Under full counting, each article was counted once for every distinct institution listed in the author affiliations. Under fractional counting, each institution was assigned a weight of 1/k for an article involving k distinct institutions. Fractional/Full represents the mean fractional credit per affiliated publication, whereas Full/Fractional is its inverse and provides an approximate indication of the extent of multi‐institutional participation.

Fractional‐to‐full ratios varied considerably among the leading institutions. The University of Texas Southwestern Medical Center and the University of Toronto had the lowest ratios, at 0.183 and 0.193, respectively, indicating that their publications generally involved larger numbers of participating institutions. By contrast, Shanghai Jiao Tong University and Amylin Pharmaceuticals had the highest ratios, at 0.490 and 0.398, respectively.

#### 3.3.2 Concentration of Institutional Output and Collaboration Structure

Institution‐level publication output exhibited a long‐tailed and unequal distribution. The 10 leading institutions collectively accounted for 11.26% of the total fractional publication output, whereas all remaining institutions contributed 88.74%. The Lorenz curve deviated substantially from the line of equality, with a Gini coefficient of 0.682 (Figure 8). These indicators show that output was substantially unequal across the full institutional distribution.

**FIGURE 8:**
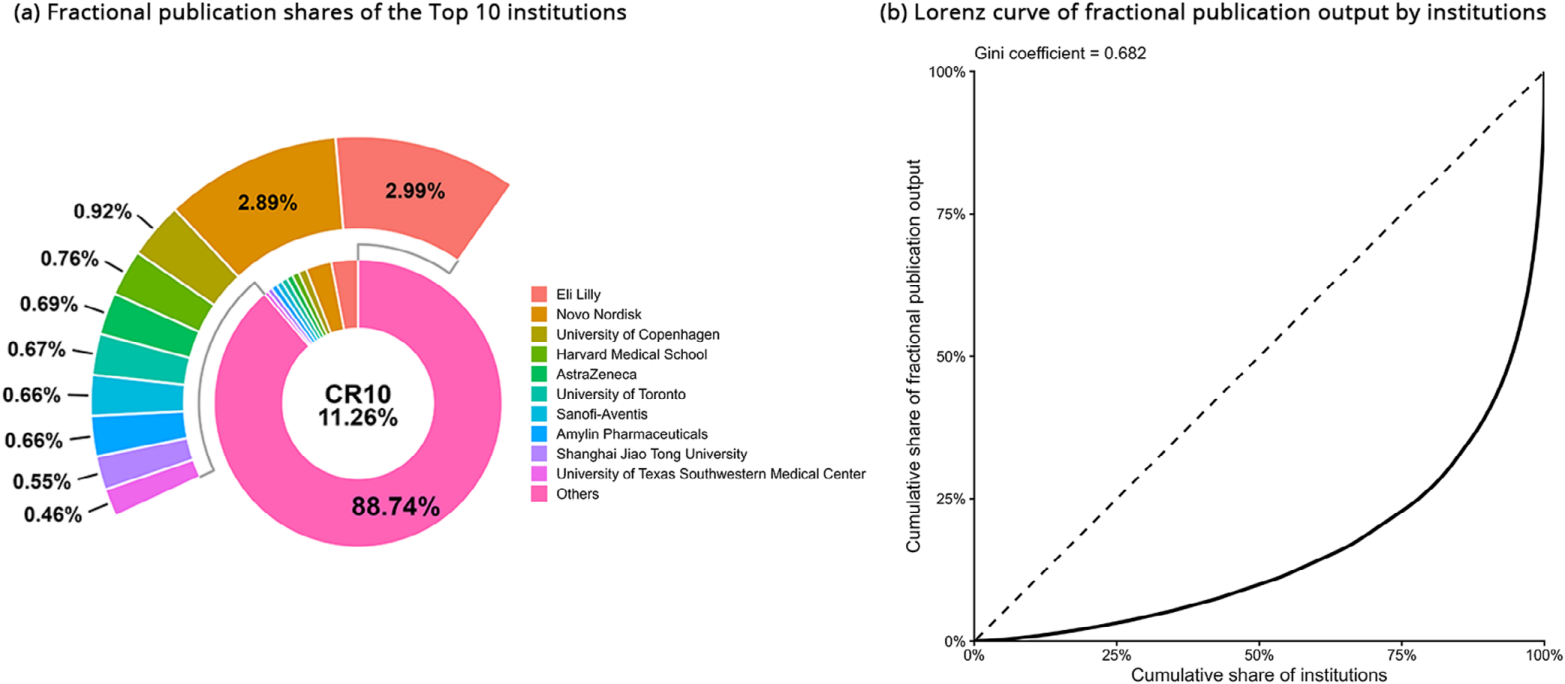
Concentration of institution‐level publication output in research on GLP‐1–based agonists in T2DM, 2004–2025. (a) Fractional publication shares of the top 10 leading institutions and all remaining institutions. CR10 denotes the cumulative share contributed by the top 10 institutions. (b) Lorenz curve of fractional publication output across institutions. The dashed diagonal represents perfect equality.

The institutional co‐authorship network comprised 38 institutions with at least 35 publications (Figure 9). Novo Nordisk had the highest TLS (457), followed by the University of Toronto (TLS = 291), Eli Lilly (TLS = 227), the University of North Carolina (TLS = 224) and the University of Texas Southwestern Medical Center (TLS = 215) (Table 4). These institutions constituted the principal weighted collaboration hubs within the thresholded network.

**FIGURE 9:**
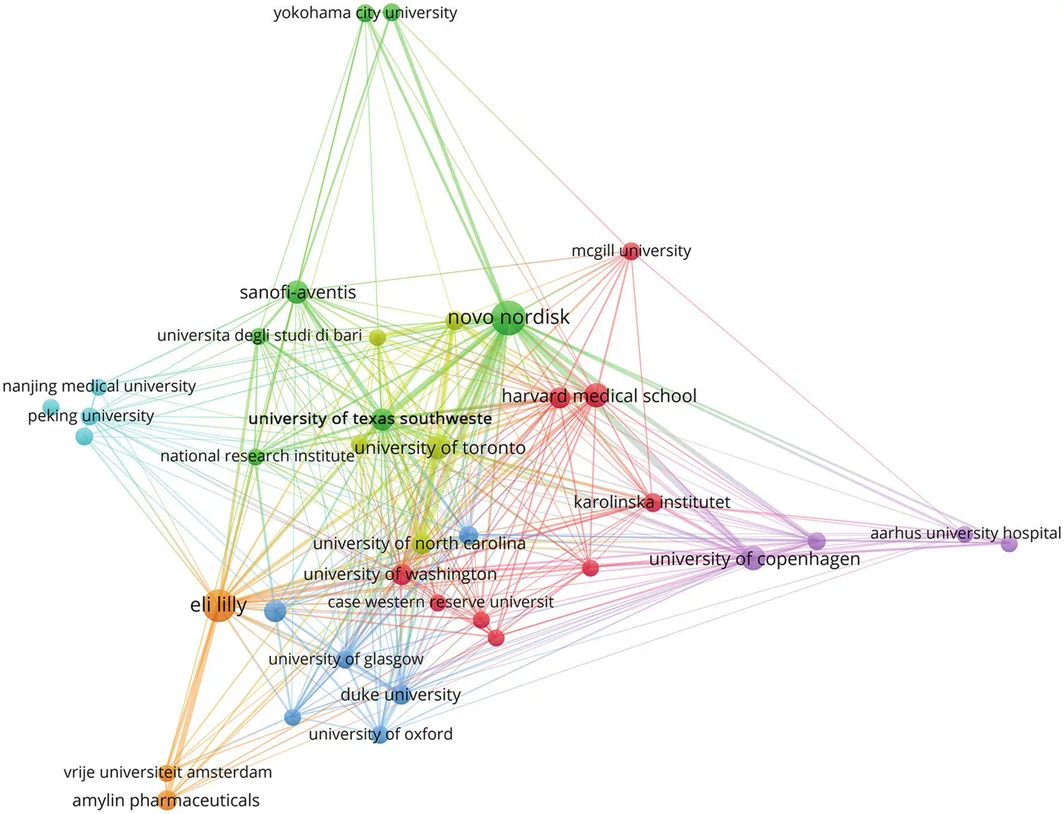
Institutional collaboration network in research on GLP‐1–based agonists in T2DM, 2004–2025. The network comprises 38 institutions with at least 35 publications. Node size represents publication output, node colour indicates network cluster membership, and link thickness represents the strength of bilateral institutional co‐authorship.

**TABLE 4:** Top 10 institutions in research on GLP‐1–based agonists in T2DM, ranked by TLS.

Rank	Institution	Degree centrality	Normalized degree centrality	TLS
1	Novo Nordisk	31	0.838	457
2	University of Toronto	28	0.757	291
3	Eli Lilly	29	0.784	227
4	University of North Carolina	28	0.757	224
5	University of Texas Southwestern Medical Center	26	0.703	215
6	University of Copenhagen	26	0.703	211
7	University of Washington	28	0.757	155
8	Harvard Medical School	24	0.649	152
9	Brigham & Women's Hospital	24	0.649	144
10	Swansea University	24	0.649	134

*Note:* The network included 38 institutions with at least 35 publications. Degree centrality represents the number of direct collaborative links of an institution. Normalized degree centrality was calculated as degree/(*N* − 1), where *N* = 38. TLS represents the sum of the strengths of all collaborative links associated with an institution.

Collaboration breadth and aggregate collaboration strength were not fully concordant. Novo Nordisk was directly connected to 31 of the other 37 institutions, corresponding to a normalized degree centrality of 0.838, whereas Eli Lilly had 29 direct connections and a normalized degree centrality of 0.784. Despite their broadly similar collaboration breadth, Novo Nordisk had approximately twice the TLS of Eli Lilly (457 vs. 227). The University of Toronto, the University of North Carolina and the University of Washington each had 28 direct connections, but their TLS values differed considerably.

Across the evaluated indicators, Novo Nordisk showed the most consistently prominent institutional profile, ranking first in full‐counted publication output, total citations and TLS, and second in fractional publication output. Eli Lilly ranked first in fractional output, second in full‐counted output and citations, and third in TLS. The University of Toronto also demonstrated strong citation and collaboration performance, ranking third in citations and second in TLS despite ranking sixth in fractional output. Shanghai Jiao Tong University and Amylin Pharmaceuticals retained comparatively high mean fractional credits per publication but did not rank among the 10 institutions with the highest TLS.

### 3.4 Author Productivity and Collaboration

Julio Rosenstock ranked first in both full‐counted (75 articles) and fractional publication output (9.22 fractional documents), followed in fractional output by Barnaby Hunt (8.28) and John B. Buse (7.08). Rosenstock also had the highest dataset‐specific h‐index (41), closely followed by Buse (40), while Ildiko Lingvay and Michael A. Nauck each had an h‐index of 27 (Table 5). The relatively high fractional rankings of authors such as Hunt and Tomonori Oura, despite their lower full publication counts, reflected differences in co‐authorship team sizes.

**TABLE 5:** Top 10 authors in research on GLP‐1–based agonists in T2DM, 2004–2025, ranked by fractional publication output.

Rank	Author	Full documents	Fractional documents	Output share (%)	h‐index
1	Rosenstock, Julio	75	9.22	0.25	41
2	Hunt, Barnaby	44	8.28	0.22	16
3	Buse, John B	63	7.08	0.19	40
4	Lingvay, Ildiko	45	5.69	0.15	27
5	Patorno, Elisabetta	35	5.36	0.14	24
6	Terauchi, Yasuo	34	5.28	0.14	14
7	Boye, Kristina S	32	5.04	0.13	16
8	Nauck, Michael A	35	4.92	0.13	27
9	Seino, Yutaka	31	4.92	0.13	17
10	Oura, Tomonori	22	4.74	0.13	10

*Note:* Full documents represent the number of included publications authored by each researcher. Fractional documents were calculated by assigning each author a weight of 1/k for a publication with k distinct authors. Output share represents the percentage of the total fractional publication output attributed to each author. The h‐index was calculated exclusively from the publications included in the present dataset and does not represent the authors' lifetime h‐index. One article without author information in the AF field was excluded from the author‐level analysis; therefore, the total fractional output was 3735. At the author level, the CR10 was 1.62%, the Herfindahl–Hirschman index was 0.00017 on a scale of 0–1, and the Gini coefficient was 0.45.

Author‐level output showed a long‐tailed distribution. The Gini coefficient was 0.45, indicating moderate inequality across individual authors. However, the top 10 authors collectively accounted for only 1.62% of the total fractional output; the Herfindahl–Hirschman index was 0.00017, and the leading author contributed only 0.25%. Thus, although author productivity varied considerably, overall output was widely distributed.

Of the 83 authors meeting the minimum threshold of 15 publications, 82 were included in the displayed connected co‐authorship network, which comprised 10 clusters (Figure 10). The network contained a densely interconnected central component together with several smaller collaborative subgroups. Rosenstock and Buse occupied prominent positions in the central network, while Hunt and Lingvay were also embedded within major collaborative groups.

**FIGURE 10:**
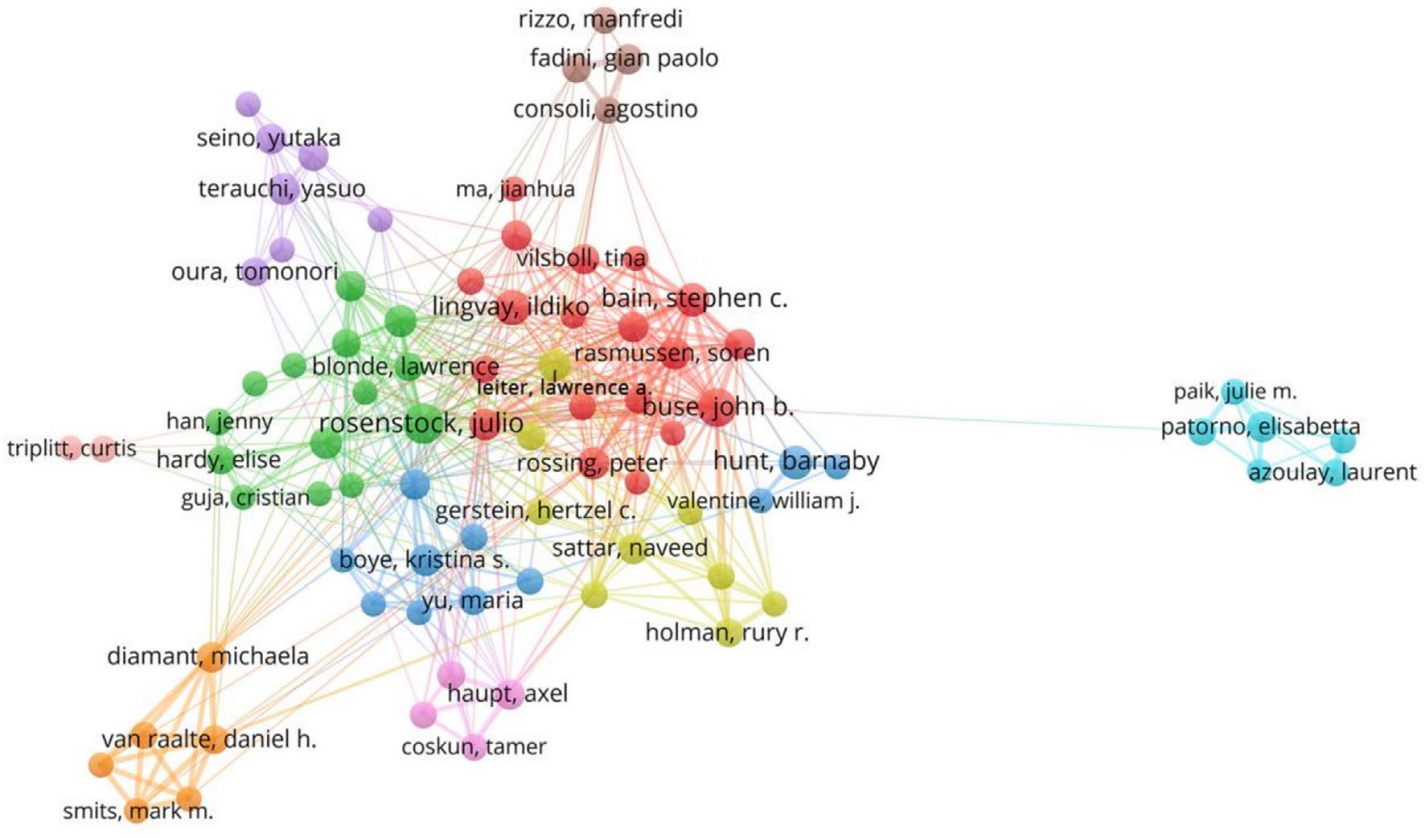
Author co‐authorship network in research on GLP‐1–based agonists in T2DM, 2004–2025. Of the 83 authors with at least 15 publications, 82 were included in the displayed connected network, which comprised 10 clusters. Node size represents publication output, node colour indicates cluster membership and link thickness represents the frequency of co‐authorship between authors.

### 3.5 Distribution of Source Journals

The 3736 included publications were distributed across 627 journals, indicating broad dissemination across publication outlets. Nevertheless, a recognizable core of specialty journals was evident. The 10 most productive journals published 1368 articles, accounting for 36.62% of all included publications. Diabetes, Obesity and Metabolism was the most productive journal, with 494 articles, followed by Diabetes Therapy (219) and Diabetes Care (186) (Figure 11a).

**FIGURE 11:**
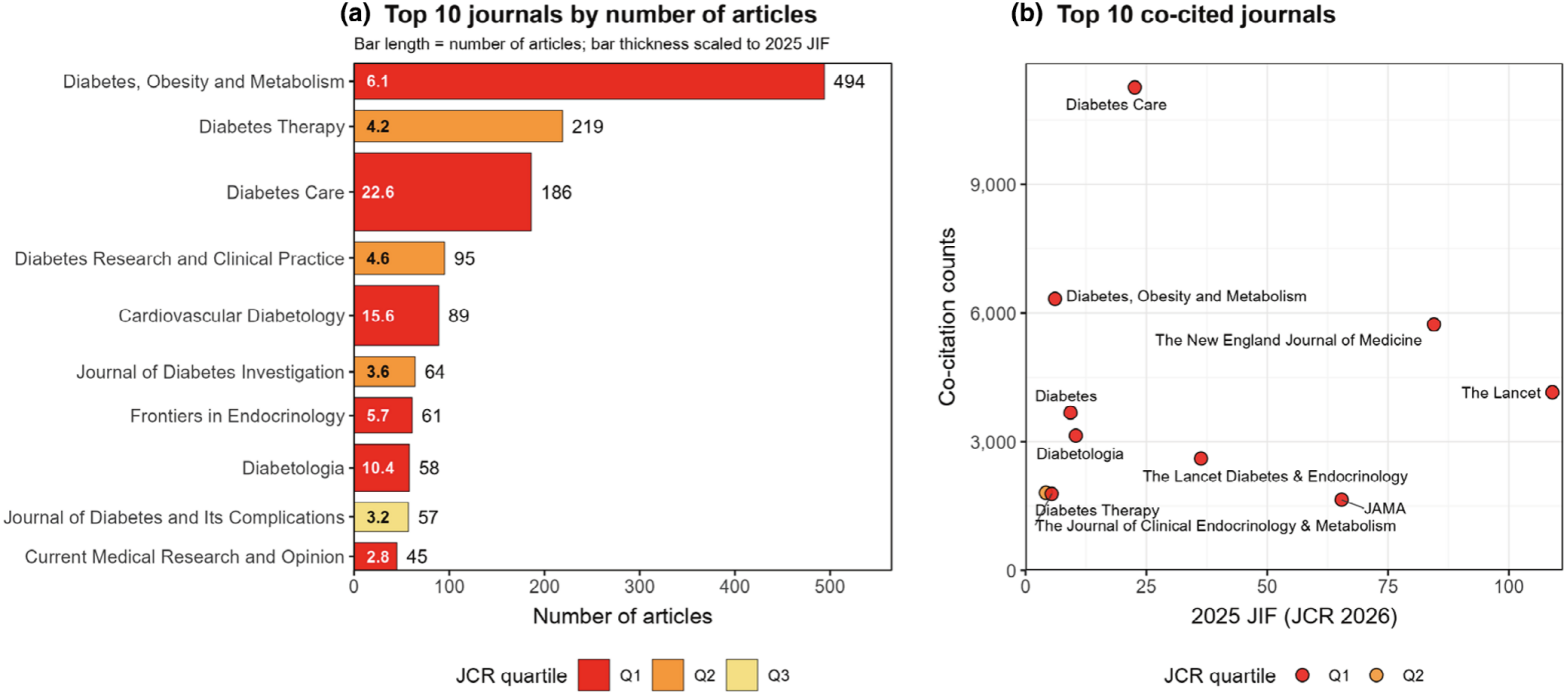
Leading publication outlets and co‐cited journals in research on GLP‐1–based agonists in T2DM, 2004–2025. (a) Top 10 journals ranked by the number of included publications. Bar length represents publication count, bar width is scaled to the 2025 JIF, and fill colour denotes the corresponding JCR quartile in the 2026 JCR release. (b) Top 10 co‐cited journals. The *x*‐axis represents the 2025 JIF, the *y*‐axis represents co‐citation frequency, and point colour denotes the corresponding JIF quartile.

The co‐citation ranking differed from the publication‐output ranking. Diabetes Care was the most frequently co‐cited journal, with 11,268 co‐citations, followed by Diabetes, Obesity and Metabolism (6334) and the New England Journal of Medicine (5739) (Figure 11b). Other highly co‐cited journals included The Lancet, Diabetes, Diabetologia and The Lancet Diabetes & Endocrinology. Diabetes, Obesity and Metabolism and Diabetes Care occupied prominent positions in both rankings, whereas The New England Journal of Medicine and The Lancet were highly co‐cited despite not ranking among the leading publication outlets.

The journal‐level networks further distinguished the outlets disseminating research on GLP‐1–based agonists from the journals constituting its cited knowledge base. The citation network of source journals comprised 68 journals with at least 10 included articles and was divided into six clusters (Figure 12a). It was centered on major diabetes and metabolism journals, particularly Diabetes, Obesity and Metabolism, Diabetes Therapy, Diabetes Care, Diabetes Research and Clinical Practice, and Cardiovascular Diabetology. The network also contained groups broadly associated with clinical therapeutics, endocrinology, cardiovascular and metabolic medicine, and health services research.

**FIGURE 12:**
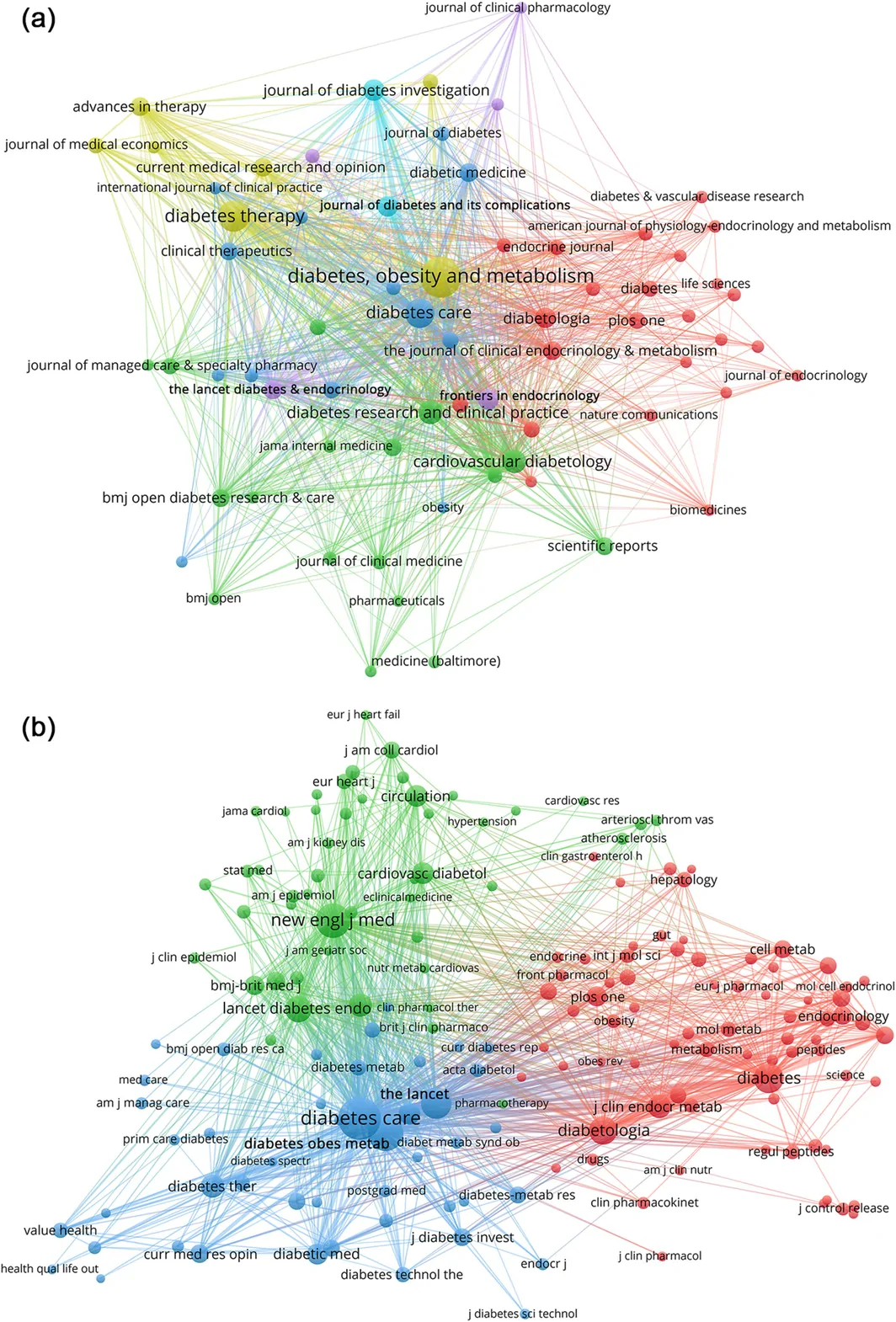
Journal citation and co‐citation networks in research on GLP‐1–based agonists in T2DM, 2004–2025. (a) Citation network of source journals. Journals with at least 10 included publications were retained, resulting in 68 journals and six clusters. Nodes represent source journals, node size reflects publication output and link thickness represents the strength of direct citation relationships between journals. (b) Co‐citation network of cited journals. Journals with at least 100 citations were retained, resulting in 168 journals and three clusters; node size reflects citation frequency, and link thickness represents co‐citation strength.

The co‐citation network comprised 168 journals with at least 100 citations and contained three clusters (Figure 12b). It retained a strong diabetes‐specialty core centred on Diabetes Care, Diabetes, Obesity and Metabolism, Diabetologia, and Diabetes, while also showing the prominent influence of general medical and cardiovascular journals, including The New England Journal of Medicine, The Lancet, Circulation and the European Heart Journal.

The dual‐map overlay revealed three dominant citation trajectories (Figure 13). Research published in the Medicine/Medical/Clinical domain primarily cited literature in the Molecular Biology/Genetics and Health/Nursing/Medicine domains, while publications in the Molecular Biology/Immunology domain predominantly cited work in Molecular Biology/Genetics. These citation paths indicate that the clinically oriented research front integrated evidence from biomedical mechanisms, clinical medicine and healthcare research.

**FIGURE 13:**
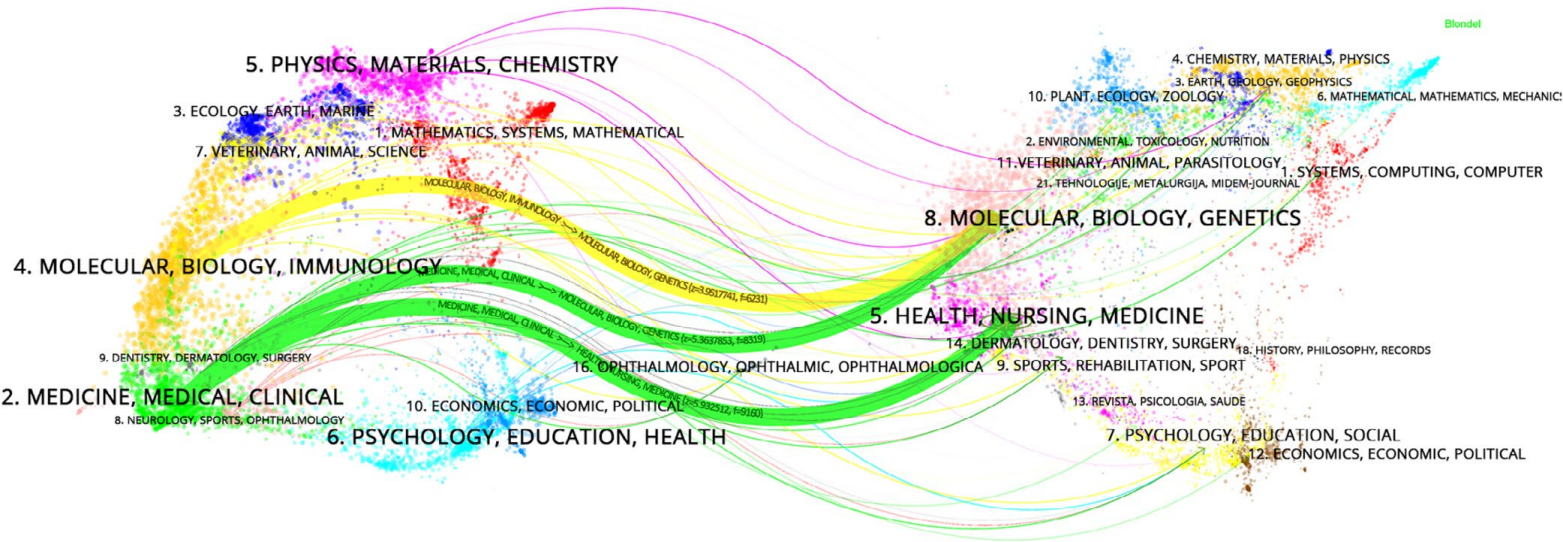
Dual‐map overlay of journals in research on GLP‐1–based agonists in T2DM, 2004–2025. The base map represents the global journal landscape derived from the JCR. Citing journals, representing the research front, are positioned on the left, while cited journals, representing the knowledge base, are positioned on the right. Coloured paths represent aggregated citation trajectories between disciplinary domains.

### 3.6 Keyword‐Based Thematic Structure and Evolution

#### 3.6.1 Core Thematic Structure and Keyword Connectivity

Of the 149 author keywords occurring at least nine times, the 100 most frequent were visualized in the co‐occurrence network and grouped into six clusters (Figure 14). The scope‐defining terms T2DM and GLP‐1 receptor agonists had the highest total link strengths. Among the more discriminative terms, liraglutide (TLS = 1064), semaglutide (874), exenatide (736), glycemic control (726), SGLT‐2 inhibitors (527), obesity (518) and dulaglutide (500) showed the strongest connectivity (Table 6). Tirzepatide ranked 19th, with a TLS of 220.

**FIGURE 14:**
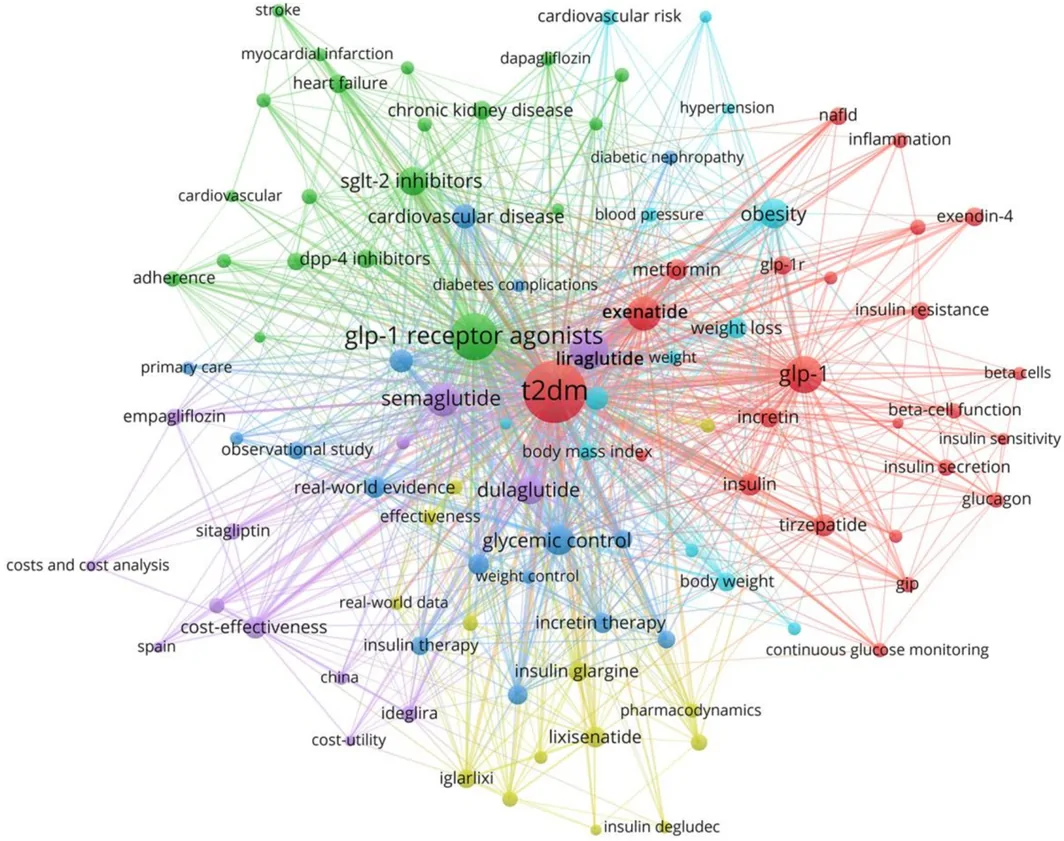
Author‐keyword co‐occurrence network in research on GLP‐1–based agonists in T2DM, 2004–2025. Of the 149 author keywords occurring at least nine times, the 100 most frequent were displayed, forming six clusters. Node size represents occurrence frequency, node colour indicates cluster membership and link thickness represents co‐occurrence strength.

**TABLE 6:** Top 20 keywords in research on GLP‐1–based agonists in T2DM, 2004–2025, ranked by TLS.

Rank	Keyword	TLS	Rank	Keyword	TLS
1	T2DM	4783	11	HbA1c	327
2	GLP‐1 receptor agonists	2388	12	Cardiovascular disease	316
3	Liraglutide	1064	13	Cost‐effectiveness	313
4	GLP‐1	953	14	Antidiabetic drugs	295
5	Semaglutide	874	15	Real‐world evidence	288
6	Exenatide	736	16	Basal insulin	261
7	Glycemic control	726	17	Insulin	235
8	SGLT‐2 inhibitors	527	18	Incretin therapy	228
9	Obesity	518	19	Tirzepatide	220
10	Dulaglutide	500	20	Hypoglycemia	216

The high connectivity of T2DM and GLP‐1 receptor agonists was expected because these terms defined the scope of the dataset. Beyond these scope‐defining terms, the network showed closely interconnected themes involving incretin biology and insulin physiology; individual GLP‐1–based therapies; glycemic management and insulin‐based combination regimens; obesity and body‐weight outcomes; cardiovascular and renal complications; comparative antidiabetic treatment; and real‐world and pharmacoeconomic evaluation. Although the six clusters were distinguishable, their extensive interconnections indicated substantial thematic integration across pharmacological, clinical and outcomes‐oriented research.

#### 3.6.2 Temporal Evolution of Major Research Themes

The temporal heatmap and annual frequency plots revealed a progressive shift in drug‐ and outcome‐related research interests (Figures 15 and 16). Exenatide and exendin‐4 were comparatively prominent during the earlier years, whereas liraglutide became increasingly visible during the mid‐to‐late 2010s. In the most recent period, semaglutide and tirzepatide showed marked increases, accompanied by growing attention to obesity, weight loss, SGLT‐2 inhibitors, cardiovascular disease and chronic kidney disease. Dulaglutide remained prominent through the late 2010s and early 2020s, while interest in cost‐effectiveness and insulin‐based regimens fluctuated across the study period.

**FIGURE 15:**
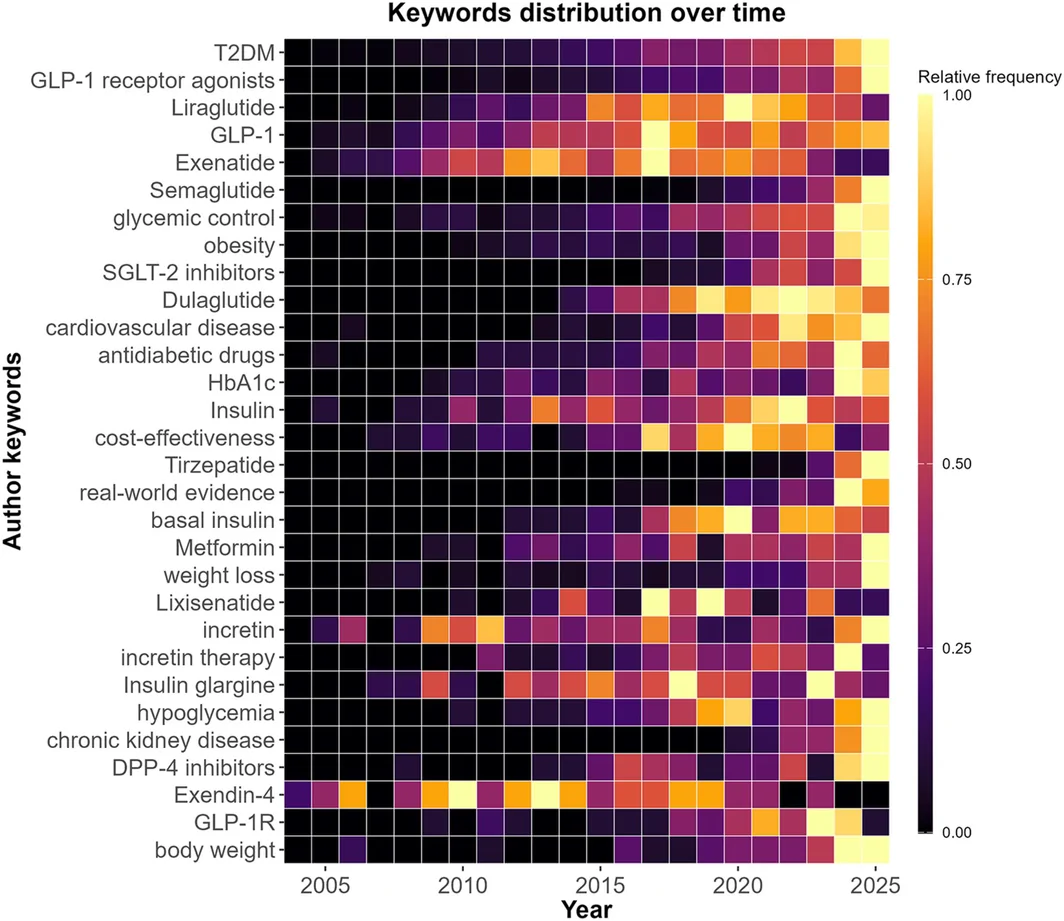
Temporal heatmap of the top 30 author keywords in research on GLP‐1–based agonists in T2DM, 2004–2025. Keywords are ordered by total occurrence frequency. Annual frequencies for each keyword were independently rescaled to a 0–1 range using within‐keyword min‐max normalization. Colour intensity therefore represents the relative annual frequency of each keyword compared with its own temporal range and should not be interpreted as an absolute comparison between different keywords.

**FIGURE 16:**
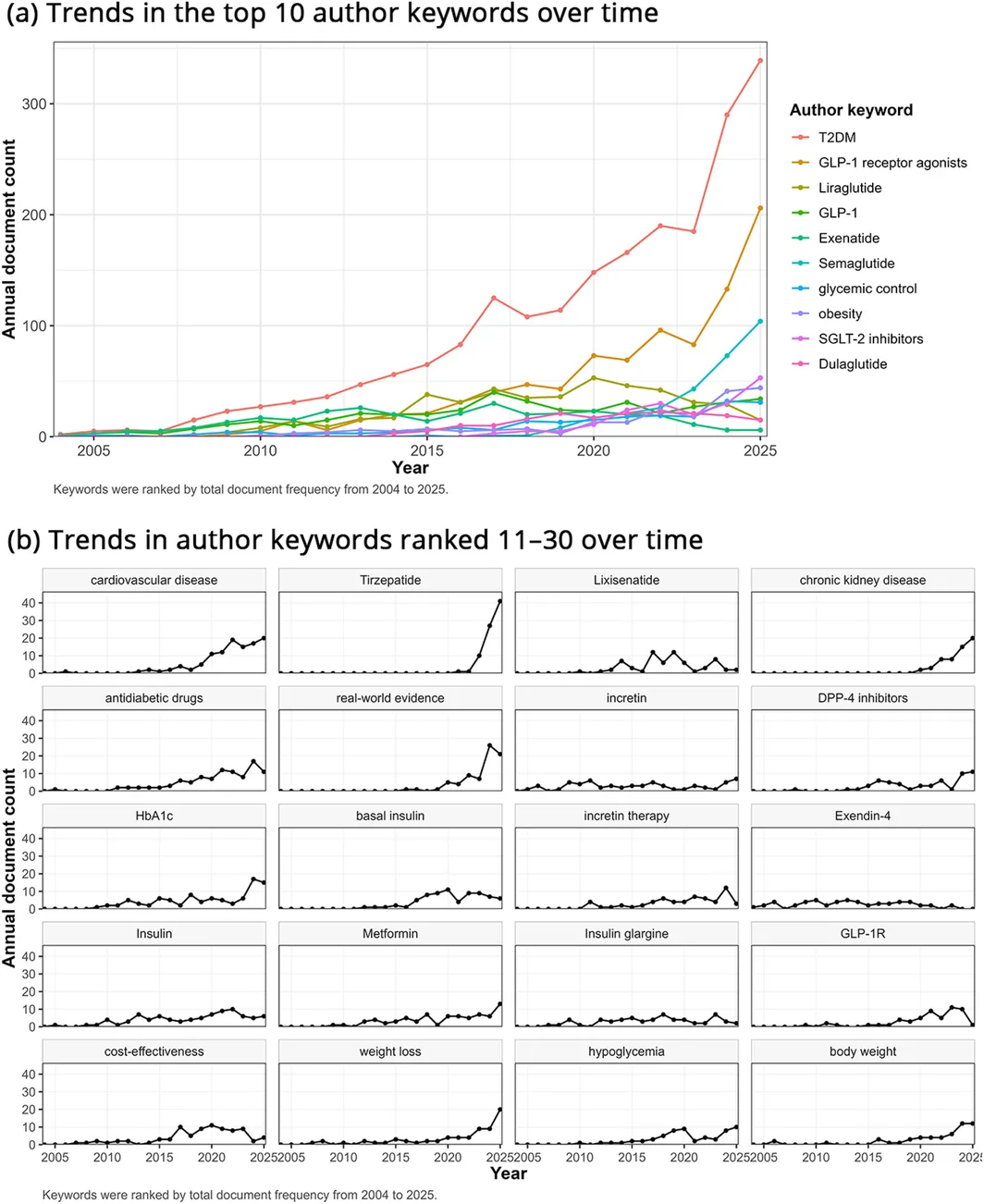
Annual occurrence trends of the 30 most frequent author keywords in research on GLP‐1–based agonists in T2DM, 2004–2025. (a) Annual document frequencies of the 10 most frequent author keywords. (b) Annual document frequencies of author keywords ranked 11–30, displayed using a common y‐axis scale to facilitate comparison within the panel. Keywords were ranked by their total document frequency across the study period.

Keywords were normalized in the heatmap and their colour intensity represents temporal variation relative to its own annual range rather than differences in absolute frequency between keywords.

Timeline analysis identified six clusters labelled ‘Diabetes mellitus,’ ‘GLP‐1 receptor agonist,’ ‘real‐world evidence,’ ‘cardiovascular disease,’ ‘chronic kidney disease,’ and ‘non‐alcoholic fatty liver disease’ (Figure 17). The diabetes mellitus and GLP‐1 receptor agonist clusters extended across most of the study period, reflecting the persistent core of the field. The real‐world evidence cluster evolved from glycemic control and body‐weight research toward extended‐release formulations, continuous glucose monitoring, real‐world evidence, fixed‐ratio combinations and oral and subcutaneous semaglutide. The cardiovascular disease cluster expanded from cardiovascular disease and blood pressure to cardiovascular outcomes, SGLT‐2 inhibitors, major adverse cardiovascular events and renal outcomes. The non‐alcoholic fatty liver disease cluster linked earlier mechanistic topics, including oxidative stress and insulin resistance, with later attention to metabolic syndrome and fatty liver disease.

**FIGURE 17:**
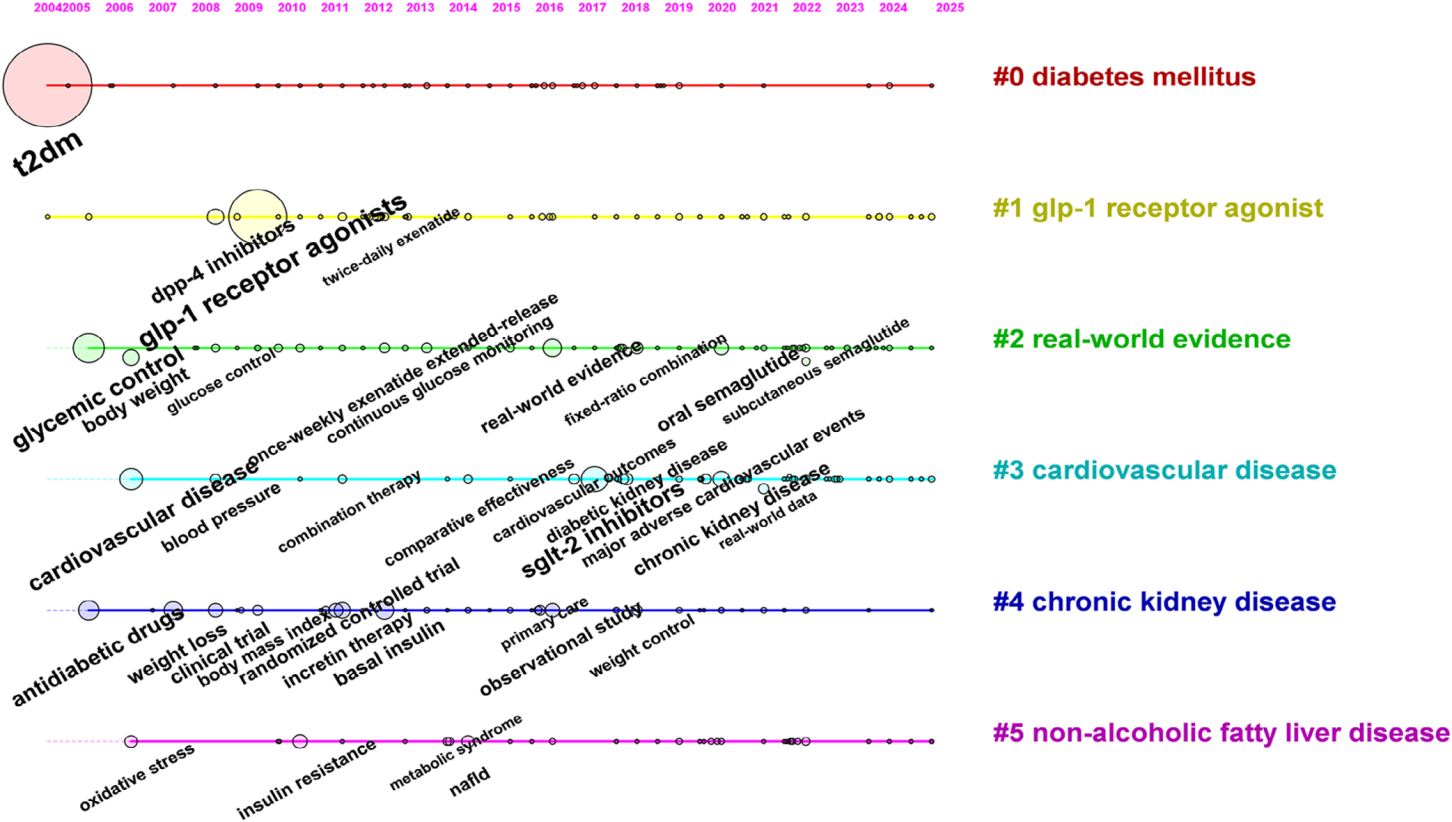
Timeline visualization of author‐keyword clusters in research on GLP‐1–based agonists in T2DM, 2004–2025. Six clusters were identified and labelled using the log‐likelihood ratio algorithm: ‘Diabetes mellitus’, ‘GLP‐1 receptor agonist’, ‘real‐world evidence’, ‘cardiovascular disease’, ‘chronic kidney disease’ and ‘non‐alcoholic fatty liver disease’. The horizontal axis represents publication year, and the vertical axis displays the keyword clusters. Nodes represent keywords, with larger nodes indicating higher occurrence frequencies; links represent co‐occurrence relationships.

#### 3.6.3 Keyword Bursts

Burst analysis identified 20 author keywords that experienced pronounced increases in research attention during specific periods (Figure 18). GLP‐1 exhibited the strongest burst (strength = 21.04), lasting from 2008 to 2018. Several early and sustained bursts were associated with incretin physiology and established treatment strategies, including incretin mimetics (strength = 7.15; 2004–2010), insulin secretion (5.56; 2004–2014), insulin glargine (6.52; 2007–2019) and insulin therapy (8.49; 2014–2020).

**FIGURE 18:**
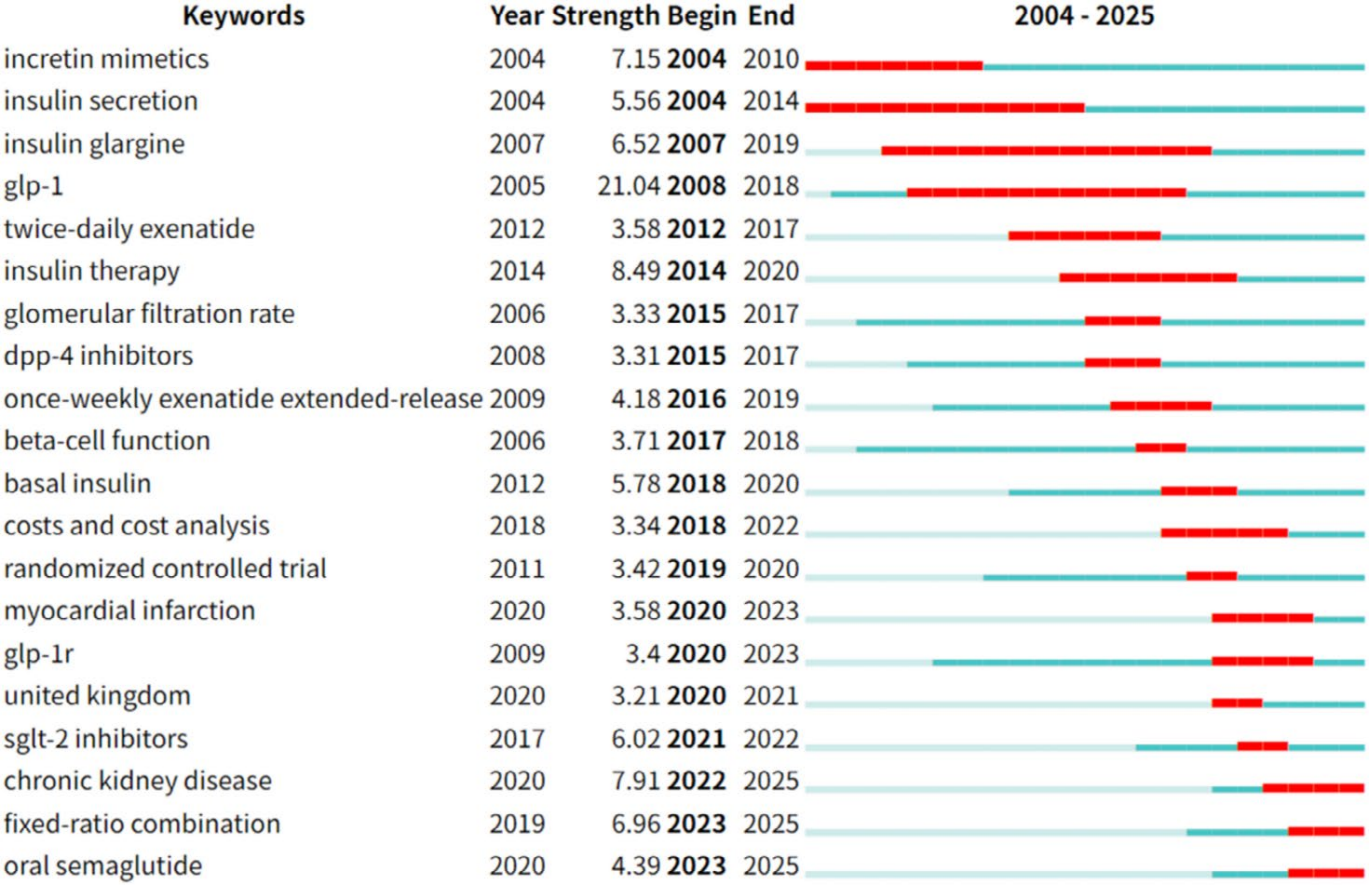
Top 20 author keywords with the strongest occurrence bursts in research on GLP‐1–based agonists in T2DM, 2004–2025. Red segments indicate periods of pronounced increases in keyword occurrence, and burst strength quantifies the intensity of each increase. ‘Year’ denotes the first year in which the keyword appeared in the dataset, whereas ‘Begin’ and ‘End’ indicate the detected burst interval.

Subsequent bursts reflected increasing attention to treatment comparisons, economic evaluation and cardiometabolic outcomes. These included costs and cost analysis (strength = 3.34; 2018–2022), myocardial infarction (3.58; 2020–2023) and SGLT‐2 inhibitors (6.02; 2021–2022). The most recent sustained bursts involved chronic kidney disease (strength = 7.91; 2022–2025), fixed‐ratio combination therapy (6.96; 2023–2025) and oral semaglutide (4.39; 2023–2025). Collectively, the burst patterns suggest a transition from early incretin physiology and glucose‐lowering regimens toward renal outcomes, integrated treatment strategies and newer GLP‐1–based formulations.

### 3.7 Citation Impact of Included Publications and Citation Bursts of Cited References

#### 3.7.1 Citation Impact of Included Publications

The LEADER trial by Marso et al. ranked first by year‐normalized citation score, with 6891 total citations and a normalized score of 62.94 (Table 7). This score indicates that the article had received approximately 62.94 times the mean number of citations of included publications published in 2016. The 2024 trial by Perkovic et al. examining semaglutide in patients with T2DM and chronic kidney disease ranked second, with 1393 citations and a normalized score of 61.54. It was followed by the REWIND trial by Gerstein et al. (2551 citations; normalized score = 40.99), the trial comparing tirzepatide with semaglutide by Frías et al. (1564; 34.00) and the ELIXA trial by Pfeffer et al. (2007; 30.90).

**TABLE 7:** Top 10 included publications on GLP‐1–based agonists in T2DM, 2004–2024, ranked by publication‐year‐normalized citation score.

Rank	First author	Year	Article title	Journal	Total citations	Normalized citation score
1	Marso, Steven P.	2016	Liraglutide and cardiovascular outcomes in Type 2 diabetes	The New England Journal of Medicine	6891	62.94
2	Perkovic, Vlado	2024	Effects of semaglutide on chronic kidney disease in patients with Type 2 diabetes	The New England Journal of Medicine	1393	61.54
3	Gerstein, Hertzel C.	2019	Dulaglutide and cardiovascular outcomes in Type 2 diabetes (REWIND): a double‐blind, randomized placebo‐controlled trial	The Lancet	2551	40.99
4	Frias, Juan P.	2021	Tirzepatide versus semaglutide once weekly in patients with Type 2 diabetes	The New England Journal of Medicine	1564	34.00
5	Pfeffer, Marc A.	2015	Lixisenatide in patients with Type 2 diabetes and acute coronary syndrome	The New England Journal of Medicine	2007	30.90
6	Holman, Rury R.	2017	Effects of once‐weekly exenatide on cardiovascular outcomes in Type 2 diabetes	The New England Journal of Medicine	1754	30.79
7	Hernandez, Adrian F.	2018	Albiglutide and cardiovascular outcomes in patients with Type 2 diabetes and cardiovascular disease (Harmony Outcomes): a double‐blind, randomized placebo‐controlled trial	The Lancet	1506	26.14
8	Garvey, W. Timothy	2023	Tirzepatide once weekly for the treatment of obesity in people with Type 2 diabetes (SURMOUNT‐2): a double‐blind, randomized, multicentre, placebo‐controlled, Phase 3 trial	The Lancet	638	25.84
9	Kosiborod, M. N.	2024	Semaglutide in patients with obesity‐related heart failure and Type 2 diabetes	The New England Journal of Medicine	567	25.05
10	Davies, Melanie	2021	Semaglutide 2.4 mg once a week in adults with overweight or obesity, and Type 2 diabetes (STEP 2): a randomized, double‐blind, double‐dummy, placebo‐controlled, Phase 3 trial	The Lancet	1149	24.98

*Note:* The year‐normalized citation score was calculated by dividing the total citation count of each included publication by the mean citation count of all included publications published in the same year. Publications from 2025 were excluded because of their short and immature citation window and are reported separately in Table S4. Citation counts were retrieved from Web of Science on July 26, 2026.

The year‐normalized top 10 consisted predominantly of major cardiovascular outcome trials and studies addressing kidney disease, obesity and body‐weight management. Six were published in the New England Journal of Medicine and four in The Lancet. Compared with ranking by cumulative citations alone, year normalization reduced the citation‐time advantage of older articles and allowed influential recent studies to be evaluated relative to other publications from the same year.

Citation levels were generally lower in the most recent cohorts, particularly in 2025, consistent with their shorter citation‐accumulation periods (Figure S1). Therefore, publications from 2025 were excluded from the primary year‐normalized ranking and summarized separately by total citations. Within this immature cohort, the most cited publications were the cardiovascular outcome trial of oral semaglutide by McGuire et al. (294 citations), the study of GLP‐1 receptor agonist discontinuation and reinitiation by Rodriguez et al. (248), and the STRIDE trial by Bonaca et al. (114) (Table S4).

Sensitivity analysis supported the overall robustness of the normalized ranking. Eight of the mean‐based Top 10 publications were also included in the percentile‐based Top 10 (Table S5). Across all 3147 publications from 2004 to 2024, the mean‐based year‐normalized citation score and the within‐year Hazen citation percentile were strongly correlated (Spearman's ρ = 0.982, *p* < 0.001).

#### 3.7.2 Citation Bursts Among Cited References

Citation‐burst analysis identified 25 cited references that experienced pronounced increases in citation frequency during specific periods (Figure 19; Table S6). The references were displayed chronologically according to burst onset rather than ranked by burst strength. The earliest bursts were associated with pivotal exenatide trials published in 2004 and 2005. These included the studies by Kendall et al. (strength = 39.67; 2005–2010), DeFronzo et al. (37.77; 2005–2010) and Buse et al. (33.75; 2005–2009), reflecting the early emphasis on glycemic control and weight outcomes.

**FIGURE 19:**
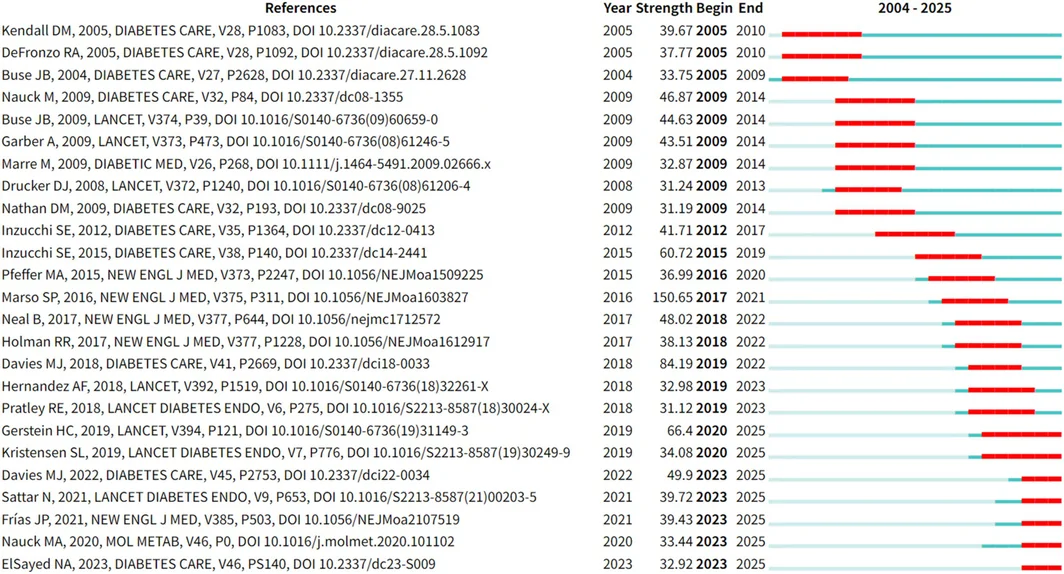
Top 25 references with the strongest citation bursts in research on GLP‐1–based agonists in T2DM, 2004–2025. Red segments indicate periods during which each cited reference experienced a pronounced increase in citation frequency, and burst strength quantifies the intensity of the increase. References are displayed primarily in chronological order according to burst onset.

From 2009 onward, citation bursts shifted toward the liraglutide LEAD program and comparative incretin‐based treatment. Prominent examples included LEAD‐2 by Nauck et al. (strength = 46.87), LEAD‐6 by Buse et al. (44.63), LEAD‐3 by Garber et al. (43.51) and LEAD‐1 by Marre et al. (32.87), all of which showed bursts beginning in 2009. Consensus recommendations also became increasingly influential, including the 2012 and 2015 ADA/EASD statements, with burst strengths of 41.71 and 60.72, respectively.

Subsequent bursts reflected the growing importance of cardiovascular and cardiorenal outcome evidence. The LEADER trial by Marso et al. exhibited the strongest burst among all cited references (strength = 150.65; 2017–2021), followed by the 2018 ADA/EASD consensus report (84.19; 2019–2022) and the REWIND trial by Gerstein et al. (66.40; 2020–2025). Other prominent references included the CANVAS, EXSCEL and Harmony Outcomes trials, as well as systematic reviews and meta‐analyses of cardiovascular, mortality and kidney outcomes.

Seven cited references had bursts extending through 2025. These included the REWIND trial, two systematic reviews and meta‐analyses of cardiovascular and kidney outcomes, the 2022 ADA/EASD consensus report, the tirzepatide‐versus‐semaglutide trial, a state‐of‐the‐art review of GLP‐1 receptor agonists, and the 2023 Standards of Care recommendations. Overall, the burst trajectory shifted from early glycemic efficacy and comparative treatment trials toward cardiovascular and kidney outcomes, newer GLP‐1–based therapies, evidence synthesis and clinical guidance.

## 4 Discussion

### 4.1 Expansion of the Field and Evolution of Research Themes

Annual output increased from 7 articles in 2004 to 589 in 2025. Selective‐only studies (Dataset 1) accounted for 94.86% of all included publications and therefore continued to define the cumulative knowledge base. However, mixed/comparative and multi‐receptor‐only studies first appeared in 2020 and together represented 14.09% of the 2025 output. Their near‐equal annual numbers in 2025 (43 mixed/comparative and 40 multi‐receptor‐only articles) suggest that newer agents are being studied both as a distinct therapeutic platform and in relation to established selective GLP‐1RAs. Thus, the recent expansion should be interpreted as diversification within a literature still dominated by studies of selective GLP‐1RAs, rather than as a wholesale shift toward studies of GLP‐1–based multi‐receptor agonists. The concurrent representation of endocrinology, pharmacology, general medicine, experimental medicine and cardiovascular science further indicates that this diversification extends across mechanistic, therapeutic and outcomes‐oriented research.

The findings suggest four broad developmental stages. The foundational period (2004–2008) centered on incretin physiology and the early clinical evaluation of exenatide, including glycemic and body‐weight effects [14, 15, 16, 17]. During therapeutic expansion (2009–2015), liraglutide and the LEAD program [18, 19, 20, 21] became prominent, alongside comparative glucose‐lowering strategies, basal‐insulin combinations, economic evaluation and clinical recommendations [22, 23]. The cardiovascular‐outcomes period (2016–2020) was marked by the influence of major outcome trials, particularly LEADER and REWIND [24, 25, 26], within a regulatory environment shaped by the 2008 US Food and Drug Administration cardiovascular‐risk guidance [27]. From 2021 onward, the field entered a phase of cardiovascular‐kidney‐metabolic integration and therapeutic diversification, characterized by semaglutide, the dual GIP/GLP‐1 receptor agonist tirzepatide, obesity and weight loss, chronic kidney disease, fixed‐ratio combinations, oral formulations and real‐world evidence [25, 28, 29, 30, 31, 32, 33].

The convergence of complementary bibliometric signals strengthens this interpretation. LEADER ranked first by publication‐year‐normalized citation score and also produced the strongest citation burst, whereas REWIND combined a high normalized rank with a burst extending through 2025 [24, 25, 26]. The 2024 semaglutide kidney‐outcomes trial ranked second after publication‐year normalization despite its short citation window [25]. More broadly, the normalized Top 10 was dominated by cardiovascular outcome trials and studies of kidney disease, obesity and body‐weight management [24, 25, 26, 28, 29, 30, 31, 34, 35, 36]. The close correlation between the mean‐based normalized score and the within‐year Hazen citation percentile (Spearman's ρ = 0.982), together with eight overlapping publications in the two Top 10 lists, supports the stability of the ranking across two approaches. It does not, however, convert citation impact into a measure of methodological quality or clinical benefit. The separate treatment of the immature 2025 cohort was therefore appropriate and avoids overinterpreting rapidly accumulating citations in a short observation window.

The thematic transition is also clinically coherent with diabetes recommendations integrating individualized glycemic management [37], cardiovascular risk reduction [38], kidney protection [39] and weight management [40]. Nevertheless, temporal concordance between bibliometric patterns and guideline development does not establish that one caused the other; both may reflect the same underlying accumulation of outcome evidence. Similarly, the prominence of SGLT‐2 inhibitors should be interpreted as comparative and combination‐therapy context within contemporary T2DM management [41], not as expansion of the corpus beyond its prespecified scope of GLP‐1–based agonists. The non‐alcoholic fatty liver disease cluster likewise represents an adjacent metabolic research branch connecting insulin resistance, oxidative stress, metabolic syndrome and fatty liver disease [42], but its presence alone does not demonstrate an established clinical indication.

### 4.2 Geographic Concentration and International Collaboration

Research participation extended to 80 countries/regions, but the distribution of fractional output was strongly unequal. The 10 leading countries accounted for 79.01% of output, the United States and China together contributed 43.11%, and the country‐level Gini coefficient was 0.837. This concentration contrasts with the comparatively limited contributions from much of Africa, Central Asia and parts of South America. It indicates that the global evidence base has been produced predominantly within a relatively small group of research systems, which may influence the populations, care settings and implementation conditions represented in the literature.

The country results also show why output, citation influence, collaboration breadth and collaboration intensity should not be treated as interchangeable. The United States led in publication output, citations and TLS, while both the United States and the United Kingdom were connected to every other country/region in the thresholded network. The United Kingdom and Denmark achieved higher citations per publication than the United States, despite smaller outputs. China ranked second in output and remained broadly connected, but ranked tenth in TLS and had a markedly lower MCP proportion than the United Kingdom, Germany, Canada and the Netherlands. These differences suggest distinct national research models: some systems combine high domestic output with selective international links, whereas others participate disproportionately in repeated multinational collaborations. High SCP shares do not imply weak research quality, and high MCP shares do not by themselves demonstrate equitable scientific participation; the indicators instead describe different collaboration structures.

Large cardiovascular and kidney outcome trials require extensive recruitment networks, long follow‐ups and substantial organizational resources, which plausibly favour countries with mature trial infrastructure and established academic‐industry networks. Broader multinational participation is nevertheless important because treatment response, tolerability, access, adherence, background therapy and cardiovascular‐kidney risk vary across health systems and patient populations [43]. A fuller assessment of global participation would consider not only whether underrepresented regions appear as participating sites, but also whether investigators from those regions contribute to study leadership, corresponding authorship, analysis and interpretation. At the same time, the observed geographic imbalance must be interpreted in light of the English‐language and Web of Science restrictions, which may underrepresent relevant local and regional publications.

### 4.3 Institutional and Author Participation

The institutional results reveal a research ecosystem built around both pharmaceutical and academic hubs. Novo Nordisk and Eli Lilly occupied the two leading positions in full‐counted output and citations and remained the two largest contributors after fractional allocation. This consistency across counting and network measures indicates sustained participation across many publications and collaborations rather than prominence created solely by repeated full counting of multi‐institutional articles. Academic centers such as the University of Toronto, the University of Copenhagen, the University of Texas Southwestern Medical Center, and the University of North Carolina also ranked highly, illustrating the complementary roles of drug development organizations, clinical investigators, trial networks and evidence‐generating academic centers.

Fractional‐to‐full ratios add information that simple publication rankings cannot provide. The low ratios for the University of Texas Southwestern Medical Center and the University of Toronto are consistent with participation in articles involving larger numbers of institutions, whereas the higher ratios for Shanghai Jiao Tong University and Amylin Pharmaceuticals indicate larger average fractional credit per affiliated article and, therefore, fewer participating institutions on average. These ratios describe team structure rather than scientific independence or study quality. Likewise, affiliation‐based prominence should not be equated with sponsorship, funding control, or ownership of study design and reporting; those questions require article‐level funding and contributorship data.

Institutional output was unequal across the full distribution (Gini = 0.682), yet the leading 10 institutions accounted for only 11.26% of fractional output. The apparent tension between these measures reflects a long‐tailed structure: many institutions contributed very small amounts, producing overall inequality, while no small group captured most of the field. Author‐level production was even more dispersed. The author CR10 was 1.62%, the HHI was 0.00017, the leading author contributed only 0.25%, and the Gini coefficient was 0.45. At the same time, 82 of 83 threshold‐eligible authors formed a connected network with 10 clusters. These results support a hub‐and‐network model in which recurrent investigators and institutions provide continuity across clinical programs, while the majority of knowledge production is distributed across a much wider collaborative community.

### 4.4 Publication Outlets and the Cited Knowledge Base

The 3736 articles were distributed across 627 journals. The 10 most productive outlets published 36.62% of the corpus. This combination of broad dispersion and a recognizable specialty core suggests a layered communication structure. Diabetes and metabolism journals provide continuity for drug‐specific efficacy, safety, comparative treatment and management research, with Diabetes, Obesity and Metabolism, Diabetes Therapy and Diabetes Care serving as the principal publication outlets. In contrast, the New England Journal of Medicine and The Lancet were prominent in the co‐cited knowledge base despite not being among the most productive source journals. Landmark trials may therefore achieve their greatest field‐wide influence through general medical journals, while the cumulative development and application of the evidence remain anchored in specialist outlets.

Diabetes Care and Diabetes, Obesity and Metabolism were prominent as both publication outlets and co‐cited journals, linking the active research front to its field‐specific knowledge base. The dual‐map trajectories add a disciplinary dimension: clinically oriented articles drew on molecular and genetic research as well as health and medical literature, while molecular and immunological work cited the biomedical knowledge base. This pattern is consistent with bidirectional translation between incretin biology, therapeutic development, clinical outcomes and healthcare evaluation.

### 4.5 Implications for Clinical Interpretation and Future Research

The expansion of research on GLP‐1–based agonists has not been uniform across agents, outcomes, or study settings. Selective GLP‐1RAs are supported by extensive programs evaluating glycemic efficacy and cardiovascular outcomes, whereas tirzepatide has been evaluated in head‐to‐head trials against semaglutide for glycemic and weight outcomes and against dulaglutide for cardiovascular outcomes [28, 44]. By comparison, the evidence for GLP‐1–based multi‐receptor agonists other than tirzepatide remains newer and less mature. Representative studies of retatrutide and survodutide in T2DM have primarily examined glycemic control, body weight and short‐ to intermediate‐term safety [45, 46]. Evidence for these agents is therefore heterogeneous in duration and outcome coverage, particularly for durable cardiorenal outcomes and direct comparisons among different receptor combinations.

Treatment sustainability in routine care represents another less‐developed strand. Real‐world studies of GLP‐1–based agonists have documented incomplete adherence, discontinuation and reinitiation, with variation according to age, comorbidity and socioeconomic circumstances [33, 47, 48]. Less is known about how treatment interruptions, tolerability, affordability and mode of administration interact to shape long‐term effectiveness. Patient‐centered evidence is especially limited: a recent systematic review identified only nine studies examining patients' experiences with GLP‐1–based agonists, of which only three used qualitative interviews [49]. Real‐world research integrating clinical outcomes with treatment continuity, healthcare use, cost and patient‐reported experience would complement the efficacy evidence generated in controlled trials.

Evidence is also uneven across populations and healthcare systems. A meta‐epidemiological review of T2DM randomized trials reported underrepresentation of racial and ethnic minority populations relative to their disease burden, while an observational study identified racial, ethnic and socioeconomic differences in the use of selective GLP‐1RAs [50, 51]. Together with the geographic concentration observed in the present analysis, these findings indicate that the generalizability of the evidence and the equitable delivery of these therapies remain incompletely characterized. Studies combining more representative recruitment with analyses of local access, treatment persistence, safety and effectiveness could clarify how trial‐based evidence on GLP‐1–based agonists translates across diverse populations and care settings.

### 4.6 Limitations

Several limitations define the interpretive boundary of this study. First, the analysis was restricted to English‐language original articles indexed in the Web of Science Core Collection. Database and language coverage may have influenced country, institution, journal and citation rankings. Second, author keywords depend on authors' terminology and were subject to harmonization; institutional disambiguation, minimum network thresholds and algorithmic clustering also require analytical choices. Consequently, smaller, weakly connected, or inconsistently labelled themes may be underrepresented. Third, institutional contributions were assessed using author‐affiliation data rather than article‐level funding or sponsorship information; therefore, the prominence of pharmaceutical companies in the institutional analysis should not be interpreted as evidence of funding involvement, commercial influence, or investigator independence. Article‐level funding, sponsorship and conflict‐of‐interest disclosures were outside the scope of the bibliometric analysis. Accordingly, institutional prominence should not be interpreted as evidence of funding involvement, commercial influence, or investigator independence. Finally, citation counts, co‐citations and bursts measure scholarly attention rather than evidence quality, causal importance, or clinical benefit. Publication‐year normalization reduced the time advantage of older articles and was supported by an alternative percentile analysis, but it did not remove journal‐ and topic‐related citation differences.

## 5 Conclusion

This bibliometric analysis of 3736 original articles traces the substantial expansion of research on GLP‐1–based agonists in T2DM between 2004 and 2025. Over this period, the literature broadened from its early focus on incretin biology and glycemic efficacy to encompass cardiovascular and kidney outcomes, obesity and weight management, integrated treatment strategies and real‐world evaluation. Selective‐only studies continued to form the main body of evidence, whereas the recent growth of mixed/comparative and multi‐receptor‐only studies signals increasing diversification. Although research output was concentrated in a limited number of countries, contributions from institutions and authors were more widely distributed, producing a collaborative but geographically uneven literature. The field has increasingly adopted a cardiovascular–kidney–metabolic perspective, although the depth of evidence still varies across agents, longer‐term outcomes, routine‐care settings, patient experience and the populations and regions represented. Evidence from these less‐developed areas would place the well‐established findings on glycemic efficacy and cardiovascular outcomes in a broader long‐term, real‐world and population context.

## Author Contributions

**Yanbing Wang:** conceptualization, formal analysis, investigation, methodology, project administration, validation, visualization, writing – original draft, writing – review and editing. **Zhanru Liu:** conceptualization, data curation, formal analysis, investigation, methodology, visualization, writing – review and editing, writing – original draft. **Shifeng Wang:** formal analysis, resources, writing – review and editing, writing – original draft. **Cuiyan Lv:** formal analysis, resources, writing – review and editing, writing – original draft. **Hesham R. El‐Seedi:** writing – review and editing, writing – original draft, validation. **Shaden A. M. Khalifa:** validation, writing – review and editing, writing – original draft. **Haiyan Wang:** conceptualization, formal analysis, funding acquisition, investigation, project administration, supervision, validation, writing – review and editing, writing – original draft.

## Funding

The study was supported by Beijing University of Chinese Medicine (2023‐JYB‐XJSJJ001), Key Laboratory of Traditional Chinese Medicine Health Preservation, Ministry of Education, China (2010–12), Beijing University of Chinese Medicine (2023‐ZXFZJJ‐JW‐001), and National Foreign Expert Program (Category S) of Ministry of Human Resources and Social Security of Peoples Republic of China (S20260038).

## Ethics Statement

The authors have nothing to report. This bibliometric study analysed publicly available bibliographic records and involved no human or animal participants.

## Conflicts of Interest

The authors declare no conflicts of interest.

## Supporting information


**Data S1:** Data and Code package.zip contains data and detailed equations, analytic procedures and corresponding scripts.

**Data S2:** Supplementary Methods.docx contains supplementary information in methods section.

**File S1:** WoSCC search string.txt contains the exact WoSCC search string in text format.

**File S2:** Eligibility assessment manual for source articles included in the bibliometric analysis.pdf contains the screening manual.

**File S3:** GLP‐1‐Based Agonists Regimen‐Level Author Keyword Standardization.txt contains regimen‐level standardization dictionaries.

**File S4:** GLP‐1‐Based Agonists_Drug‐Level Author Keyword Standardization.txt contains drug‐level standardization dictionaries.

**File S5:** GLP‐1‐Based Agonists dataset sensitivity analysis report.pdf contains Spearman's rank correlation, the adjusted Rand index, and normalized mutual information.

**Figure S1:** Annual citation distributions of included publications in GLP‐1–based agonist research on T2DM, 2004–2025. Red circles and solid lines indicate mean citations per publication; blue triangles and dashed lines indicate median citations; and shading indicates the interquartile range. The *y*‐axis is shown on a log1p‐transformed scale to accommodate the strongly right‐skewed citation distributions. Publications from 2025 were excluded from the primary year‐normalized ranking because of their short citation window.
**Table S1:** Parameters used for constructing and visualizing bibliometric networks in VOSviewer.
**Table S2:** PubMed coverage of the most‐cited eligible WoSCC articles using tie‐inclusive citation thresholds.
**Table S3:** Threshold sensitivity analysis of the bibliometric networks.
**Table S4:** Top 10 included publications from the immature 2025 citation cohort, ranked by total citations.
**Table S5:** Sensitivity comparison of the Top 10 included publications identified using mean‐based year‐normalized citation scores and within‐year Hazen citation percentiles, 2004–2024.
**Table S6:** Top 25 references with the strongest citation bursts in GLP‐1–based agonist research in T2DM, 2004–2025.
**Table S6:** Top 25 references with the strongest citation bursts in GLP‐1–based agonist research in T2DM, 2004–2025.

## Data Availability

All the data in this study can be obtained upon reasonable request.

## References

[1] International Diabetes Federation , IDF Diabetes Atlas (International Diabetes Federation, 2025).

[2] A. Ceriello and F. Prattichizzo , “Variability of Risk Factors and Diabetes Complications,” Cardiovascular Diabetology 20, no. 1 (2021): 101. 10.1186/s12933-021-01289-4 PMC8106175 33962641

[3] O. Mosenzon , A. Alguwaihes , J. L. A. Leon , et al., “CAPTURE: A Multinational, Cross‐Sectional Study of Cardiovascular Disease Prevalence in Adults With Type 2 Diabetes Across 13 Countries,” Cardiovascular Diabetology 20, no. 1 (2021): 154. 10.1186/s12933-021-01344-0 PMC8317423 34315481

[4] E. Lin and K. F. Erickson , “Payer Mix Among Patients Receiving Dialysis,” JAMA 324, no. 9 (2020): 900–901. 10.1001/jama.2020.10771 32870292

[5] S. Klein , A. Gastaldelli , H. Yki‐Järvinen , and P. E. Scherer , “Why Does Obesity Cause Diabetes?,” Cell Metabolism 34, no. 1 (2022): 11–20. 10.1016/j.cmet.2021.12.012 PMC8740746 34986330

[6] D. Giugliano , M. Longo , L. Scappaticcio , et al., “BEYOND 2 Years: Durability of Metabolic Benefits by Simplification of Complex Insulin Regimens in Type 2 Diabetes,” Endocrine 83, no. 2 (2024): 399–404. 10.1007/s12020-023-03547-9 PMC10850008 37787888

[7] M. D. Kelsey , A. J. Nelson , J. B. Green , et al., “Guidelines for Cardiovascular Risk Reduction in Patients With Type 2 Diabetes: JACC Guideline Comparison,” Journal of the American College of Cardiology 79, no. 18 (2022): 1849–1857. 10.1016/S0735-1097(22)02840-6 PMC8972581 35512864

[8] F. B. Rivera , L. L. A. Cruz , J. V. Magalong , et al., “Cardiovascular and Renal Outcomes of Glucagon‐Like Peptide 1 Receptor Agonists Among Patients With and Without Type 2 Diabetes Mellitus: A Meta‐Analysis of Randomized Placebo‐Controlled Trials,” American Journal of Preventive Cardiology 18 (2024): 100679. 10.1016/j.ajpc.2024.100679 PMC11108827 38779187

[9] N. A. ElSayed , G. Aleppo , V. R. Aroda , et al., “8. Obesity and Weight Management for the Prevention and Treatment of Type 2 Diabetes: Standards of Care in Diabetes—2023,” Diabetes Care 46, no. Supplement_1 (2023): S128–S139. 10.2337/dc23-S008 PMC9810466 36507637

[10] E. U. Alum , “Optimizing Patient Education for Sustainable Self‐Management in Type 2 Diabetes,” Discover Public Health 22, no. 1 (2025): 44.

[11] S. Ciardullo , L. Savaré , F. Rea , G. Perseghin , and G. Corrao , “Adherence to GLP1‐RA and SGLT2‐I Affects Clinical Outcomes and Costs in Patients With Type 2 Diabetes,” Diabetes/Metabolism Research and Reviews 40, no. 4 (2024): e3791. 10.1002/dmrr.3791 38549238

[12] R. R. Kalyani , J. J. Neumiller , N. M. Maruthur , and D. J. Wexler , “Diagnosis and Treatment of Type 2 Diabetes in Adults: A Review,” JAMA 334, no. 11 (2025): 984–1002. 10.1001/jama.2025.5956 PMC13014273 40549398

[13] M. A. Nauck and J. J. Meier , “Incretin Hormones: Their Role in Health and Disease,” Diabetes, Obesity & Metabolism 20 (2018): 5–21. 10.1111/dom.13129 29364588

[14] D. M. Kendall , M. C. Riddle , J. Rosenstock , et al., “Effects of Exenatide (Exendin‐4) on Glycemic Control Over 30 Weeks in Patients With Type 2 Diabetes Treated With Metformin and a Sulfonylurea,” Diabetes Care 28, no. 5 (2005): 1083–1091. 10.2337/diacare.28.5.1083 15855571

[15] R. A. DeFronzo , R. E. Ratner , J. Han , D. D. Kim , M. S. Fineman , and A. D. Baron , “Effects of Exenatide (Exendin‐4) on Glycemic Control and Weight Over 30 Weeks in Metformin‐Treated Patients With Type 2 Diabetes,” Diabetes Care 28, no. 5 (2005): 1092–1100. 10.2337/diacare.28.5.1092 15855572

[16] J. B. Buse , R. R. Henry , J. Han , et al., “Effects of Exenatide (Exendin‐4) on Glycemic Control Over 30 Weeks in Sulfonylurea‐Treated Patients With Type 2 Diabetes,” Diabetes Care 27, no. 11 (2004): 2628–2635. 10.2337/diacare.27.11.2628 15504997

[17] M. A. Nauck , D. R. Quast , J. Wefers , and J. J. Meier , “GLP‐1 Receptor Agonists in the Treatment of Type 2 Diabetes‐State‐Of‐The‐Art,” Molecular Metabolism 46 (2020): 101102. 10.1016/j.molmet.2020.101102 PMC8085572 33068776

[18] M. Nauck , A. Frid , K. Hermansen , et al., “Efficacy and Safety Comparison of Liraglutide, Glimepiride, and Placebo, All in Combination With Metformin, in Type 2 Diabetes the LEAD (Liraglutide Effect and Action in Diabetes)‐2 Study,” Diabetes Care 32, no. 1 (2009): 84–90. 10.2337/dc08-1355 PMC2606836 18931095

[19] J. B. Buse , J. Rosenstock , G. Sesti , et al., “Liraglutide Once a Day Versus Exenatide Twice a Day for Type 2 Diabetes: A 26‐Week Randomised, Parallel‐Group, Multinational, Open‐Label Trial (LEAD‐6),” Lancet 374, no. 9683 (2009): 39–47. 10.1016/S0140-6736(09)60659-0 19515413

[20] A. Garber , R. Henry , R. Ratner , et al., “Liraglutide Versus Glimepiride Monotherapy for Type 2 Diabetes (LEAD‐3 Mono): A Randomised, 52‐Week, Phase III, Double‐Blind, Parallel‐Treatment Trial,” Lancet 373, no. 9662 (2009): 473–481. 10.1016/S0140-6736(08)61246-5 18819705

[21] M. Marre , J. Shaw , M. Brändle , et al., “Liraglutide, a Once‐Daily Human GLP‐1 Analogue, Added to a Sulphonylurea Over 26 Weeks Produces Greater Improvements in Glycaemic and Weight Control Compared With Adding Rosiglitazone or Placebo in Subjects With Type 2 Diabetes (LEAD‐1 SU),” Diabetic Medicine 26, no. 3 (2009): 268–278. 10.1111/j.1464-5491.2009.02666.x PMC2871176 19317822

[22] S. E. Inzucchi , R. M. Bergenstal , J. B. Buse , et al., “Management of Hyperglycemia in Type 2 Diabetes: A Patient‐Centered Approach,” Diabetes Care 35, no. 6 (2012): 1364–1379. 10.2337/dc12-0413 PMC3357214 22517736

[23] S. E. Inzucchi , R. M. Bergenstal , J. B. Buse , et al., “Management of Hyperglycemia in Type 2 Diabetes, 2015: A Patient‐Centered Approach Update to a Position Statement of the American Diabetes Association and the European Association for the Study of Diabetes,” Diabetes Care 38, no. 1 (2015): 140–149. 10.2337/dc14-2441 25538310

[24] S. P. Marso , G. H. Daniels , K. Brown‐Frandsen , et al., “Liraglutide and Cardiovascular Outcomes in Type 2 Diabetes,” New England Journal of Medicine 375, no. 4 (2016): 311–322. 10.1056/NEJMoa1603827 PMC4985288 27295427

[25] V. Perkovic , K. R. Tuttle , P. Rossing , et al., “Effects of Semaglutide on Chronic Kidney Disease in Patients With Type 2 Diabetes,” New England Journal of Medicine 391, no. 2 (2024): 109–121. 10.1056/NEJMoa2403347 38785209

[26] H. C. Gerstein , H. M. Colhoun , G. R. Dagenais , et al., “Dulaglutide and Cardiovascular Outcomes in Type 2 Diabetes (REWIND): A Double‐Blind, Randomised Placebo‐Controlled Trial,” Lancet 394, no. 10193 (2019): 121–130. 10.1016/S0140-6736(19)31149-3 31189511

[27] D. K. McGuire , N. Marx , O. E. Johansen , S. E. Inzucchi , J. Rosenstock , and J. T. George , “FDA Guidance on Antihyperglyacemic Therapies for Type 2 Diabetes: One Decade Later,” Diabetes, Obesity & Metabolism 21, no. 5 (2019): 1073–1078. 10.1111/dom.13645 30690856

[28] J. P. Frías , M. J. Davies , J. Rosenstock , et al., “Tirzepatide Versus Semaglutide Once Weekly in Patients With Type 2 Diabetes,” New England Journal of Medicine 385, no. 6 (2021): 503–515. 10.1056/NEJMoa2107519 34170647

[29] W. T. Garvey , J. P. Frias , A. M. Jastreboff , et al., “Tirzepatide Once Weekly for the Treatment of Obesity in People With Type 2 Diabetes (SURMOUNT‐2): A Double‐Blind, Randomised, Multicentre, Placebo‐Controlled, Phase 3 Trial,” Lancet 402, no. 10402 (2023): 613–626. 10.1016/S0140-6736(23)01200-X 37385275

[30] M. N. Kosiborod , M. C. Petrie , B. A. Borlaug , et al., “Semaglutide in Patients With Obesity‐Related Heart Failure and Type 2 Diabetes,” New England Journal of Medicine 390, no. 15 (2024): 1394–1407. 10.1056/NEJMoa2313917 38587233

[31] M. Davies , L. Færch , O. K. Jeppesen , et al., “Semaglutide 2.4 mg Once a Week in Adults With Overweight or Obesity, and Type 2 Diabetes (STEP 2): A Randomised, Doubleblind, Double‐Dummy, Placebo‐Controlled, Phase 3 Trial,” Lancet 397, no. 10278 (2021): 971–984. 10.1016/S0140-6736(21)00213-0 33667417

[32] D. K. McGuire , N. Marx , S. L. Mulvagh , et al., “Oral Semaglutide and Cardiovascular Outcomes in High‐Risk Type 2 Diabetes,” New England Journal of Medicine 392, no. 20 (2025): 2001–2012. 10.1056/NEJMoa2501006 40162642

[33] P. J. Rodriguez , V. Zhang , S. Gratzl , et al., “Discontinuation and Reinitiation of Dual‐Labeled GLP‐1 Receptor Agonists Among US Adults With Overweight or Obesity,” JAMA Network Open 8, no. 1 (2025): e2457349. 10.1001/jamanetworkopen.2024.57349 PMC11786232 39888616

[34] M. A. Pfeffer , B. Claggett , R. Diaz , et al., “Lixisenatide in Patients With Type 2 Diabetes and Acute Coronary Syndrome,” New England Journal of Medicine 373, no. 23 (2015): 2247–2257. 10.1056/NEJMoa1509225 26630143

[35] R. R. Holman , M. A. Bethel , R. J. Mentz , et al., “Effects of Once‐Weekly Exenatide on Cardiovascular Outcomes in Type 2 Diabetes,” New England Journal of Medicine 377, no. 13 (2017): 1228–1239. 10.1056/NEJMoa1612917 PMC9792409 28910237

[36] A. F. Hernandez , J. B. Green , S. Janmohamed , et al., “Albiglutide and Cardiovascular Outcomes in Patients With Type 2 Diabetes and Cardiovascular Disease (Harmony Outcomes): A Double‐Blind, Randomised Placebo‐Controlled Trial,” Lancet 392, no. 10157 (2018): 1519–1529. 10.1016/S0140-6736(18)32261-X 30291013

[37] Amer Diabet Assoc Prof Practice, C , et al., “9. Pharmacologic Approaches to Glycemic Treatment: Standards of Care in Diabetes‐2025,” Diabetes Care 48 (2025): S181–S206. 10.2337/dc25-S009 PMC11635045 39651989

[38] N. A. Elsayed , R. G. McCoy , G. Aleppo , et al., “10. Cardiovascular Disease and Risk Management: Standards of Care in Diabetes‐2025,” Diabetes Care 48 (2025): S207–S238. 10.2337/dc25-S010 PMC11635050 39651970

[39] N. A. Elsayed , R. G. McCoy , G. Aleppo , et al., “11. Chronic Kidney Disease and Risk Management: Standards of Care in Diabetes‐2025,” Diabetes Care 48 (2025): S239–S251. 10.2337/dc25-S011 PMC11635029 39651975

[40] AmerDiabetAssocProfessionalPractice , “8. Obesity and Weight Management for the Prevention and Treatment of Type 2 Diabetes: Standards of Care in Diabetes‐2025,” Diabetes Care 48 (2025): S167–S180. 10.2337/dc25-S008 PMC11635032 39651976

[41] B. Neal , V. Perkovic , K. W. Mahaffey , et al., “Canagliflozin and Cardiovascular and Renal Events in Type 2 Diabetes,” New England Journal of Medicine 377, no. 7 (2017): 644–657. 10.1056/NEJMoa1611925 28605608

[42] S. L. Friedman , B. A. Neuschwander‐Tetri , M. Rinella , and A. J. Sanyal , “Mechanisms of NAFLD Development and Therapeutic Strategies,” Nature Medicine 24, no. 7 (2018): 908–922. 10.1038/s41591-018-0104-9 PMC6553468 29967350

[43] S. K. Kunutsor , K. Khunti , and S. Seidu , “Racial, Ethnic and Regional Differences in the Effect of Sodium‐Glucose Co‐Transporter 2 Inhibitors and Glucagon‐Like Peptide 1 Receptor Agonists on Cardiovascular and Renal Outcomes: A Systematic Review and Meta‐Analysis of Cardiovascular Outcome Trials,” Journal of the Royal Society of Medicine 117, no. 8 (2024): 267–283. 10.1177/01410768231198442 PMC11450921 37734450

[44] S. J. Nicholls , I. Pavo , D. L. Bhatt , et al., “Cardiovascular Outcomes With Tirzepatide Versus Dulaglutide in Type 2 Diabetes,” New England Journal of Medicine 393, no. 24 (2025): 2409–2420. 10.1056/NEJMoa2505928 41406444

[45] J. Rosenstock , J. Frias , A. M. Jastreboff , et al., “Retatrutide, a GIP, GLP‐1 and Glucagon Receptor Agonist, for People With Type 2 Diabetes: A Randomised, Double‐Blind, Placebo and Active‐Controlled, Parallel‐Group, Phase 2 Trial Conducted in the USA,” Lancet 402, no. 10401 (2023): 529–544. 10.1016/S0140-6736(23)01053-X 37385280

[46] M. Blüher , J. Rosenstock , J. Hoefler , R. Manuel , and A. M. Hennige , “Dose‐Response Effects on HbA1c and Bodyweight Reduction of Survodutide, a Dual Glucagon/GLP‐1 Receptor Agonist, Compared With Placebo and Open‐Label Semaglutide in People With Type 2 Diabetes: A Randomised Clinical Trial,” Diabetologia 67, no. 3 (2024): 470–482. 10.1007/s00125-023-06053-9 PMC10844353 38095657

[47] D. Do , T. Lee , S. K. Peasah , C. B. Good , A. Inneh , and U. Patel , “GLP‐1 Receptor Agonist Discontinuation Among Patients With Obesity and/or Type 2 Diabetes,” JAMA Network Open 7, no. 5 (2024): e2413172. 10.1001/jamanetworkopen.2024.13172 PMC11127113 38787563

[48] L. Lv , B. L. Brady , L. Xie , M. Guevarra , and A. Turchin , “Adherence and Persistence Among People With Type 2 Diabetes Newly Initiating Oral Semaglutide Versus DPP‐4is in a US Real‐World Setting,” Primary Care Diabetes 18, no. 5 (2024): 511–517. 10.1016/j.pcd.2024.06.013 38991896

[49] C. K. Ibsen , M. Brostrøm Kousgaard , S. Olsen , et al., “Patients' Experiences With GLP1‐RAs ‐ a Systematic Review,” Scandinavian Journal of Primary Health Care 43, no. 2 (2025): 370–379. 10.1080/02813432.2025.2477141 PMC12090293 40071612

[50] R. Ahmed , R. J. de Souza , V. Li , L. Banfield , and S. S. Anand , “Twenty Years of Participation of Racialised Groups in Type 2 Diabetes Randomised Clinical Trials: A Meta‐Epidemiological Review,” Diabetologia 67, no. 3 (2024): 443–458. 10.1007/s00125-023-06052-w PMC10844363 38177564

[51] L. A. Eberly , L. Yang , U. R. Essien , et al., “Racial, Ethnic, and Socioeconomic Inequities in Glucagon‐Like Peptide‐1 Receptor Agonist Use Among Patients With Diabetes in the US,” JAMA Health Forum 2, no. 12 (2021): e214182. 10.1001/jamahealthforum.2021.4182 PMC8796881 35977298

