# Supplementary material for: Global Landscape and Thematic Evolution of Selective GLP‐1 Receptor Agonists and GLP‐1–Based Multi‐Receptor Agonists in Type 2 Diabetes Mellitus: A Bibliometric Analysis
Source: Endocrinol Diabetes Metab. 2026 Sep 30;9(6):e70351. doi: 10.1002/edm2.70351 (PMC13627873; doi:10.1002/edm2.70351)
Supplement: Supplementary file 1 [file EDM2-9-e70351-s003.pdf]

Dataset-Definition Sensitivity Analysis

Selective GLP-1 Receptor Agonists and Dual/Triple Receptor Agonists with GLP-1 Activity

Study base	Coverage	Core analyses
Included WoS source articles	2004–2025; n = 3,736	Annual output, keyword occurrence, TLS, and thematic clustering

Key finding. The core keyword rankings were virtually unchanged across dataset definitions: all occurrence- and TLS-based Top 10 lists overlapped completely, and all pairwise Spearman correlations exceeded 0.99. The overall distribution of aligned theme sizes was not statistically different. Limited changes in peripheral thematic boundaries were observed only after studies focusing exclusively on multi-receptor agonists were added, with the number of VOSviewer clusters decreasing from seven to six.

1. Objective and analytical design

This sensitivity analysis evaluated whether the bibliometric conclusions depended on how the scope of GLP-1 receptor agonist research was defined. The 3,736 included Web of Science (WoS) source articles were partitioned into three mutually exclusive datasets. Progressively expanded dataset combinations were then used to compare annual publication patterns, keyword occurrence frequencies, total link strength (TLS), and thematic cluster structure.

The mutually exclusive groups were Dataset 1 (selective GLP-1RA-only studies), Dataset 2 (mixed or comparative studies involving both selective GLP-1RAs and multi-receptor agonists), and Dataset 3 (studies restricted to dual/triple receptor agonists with GLP-1 activity). The annual publication figure displays these three mutually exclusive groups directly. Keyword and clustering analyses instead compare Dataset 1, Dataset 1+2, and Dataset 1+2+3 to assess the effect of progressively broadening the study scope.

2. Construction of the three sensitivity datasets

2.1 Automated dictionary-based screening

The title (TI), abstract (AB), author keywords (DE), and Keywords Plus (ID) fields were combined to approximate the WoS Topic field. Two prespecified drug dictionaries were used. The selective GLP-1RA dictionary included exenatide, liraglutide, lixisenatide, albiglutide, dulaglutide, semaglutide, efpeglenatide, and their principal brand names. The multi-receptor dictionary included tirzepatide, cotadutide, retatrutide, survodutide, mazdutide, and their principal brand names. Matching was case-insensitive and used alphanumeric boundaries to reduce false matches within longer strings.

Records without any multi-receptor drug term in TI, AB, DE, or ID entered the operational Dataset 1 directly; records containing a multi-receptor term entered manual review. Automated screening yielded 3,536 operational Dataset 1 records and 200 multi-receptor candidates.

2.2 Manual title-and-abstract review

All 200 candidates were reviewed using the title, abstract, author keywords, and matched field. Classification was based on whether the relevant drug constituted a study objective, intervention or exposure, primary comparator, or separately interpretable result—not merely whether a drug name appeared. Records mentioning a multi-receptor drug only in background text, contextual discussion, or Keywords Plus, while substantively studying a selective GLP-1RA, were assigned to Dataset 1.

Manual review reassigned 8 of the 200 candidates to Dataset 1, while 93 were classified as Dataset 2 and 99 as Dataset 3. The final mutually exclusive datasets therefore contained 3,544, 93, and 99 articles, respectively, for a total of 3,736.

2.3 Matching to WoS records and quality control

The classifications were matched to the WoS plain-text records using UT and DOI. A standardized UT match was attempted first, followed by standardized DOI when UT did not match. Each record was permitted to occur in only one dataset. Quality checks covered unmatched and ambiguous records,

UT/DOI conflicts, duplication across datasets, and closure of the total count. All records were successfully extracted into the three final datasets.

Dataset	Definition	Articles	Share of total
Dataset 1	Selective GLP-1RA-only	3,544	94.86%
Dataset 2	Mixed/comparative selective and multi-receptor studies	93	2.49%
Dataset 3	Dual/triple receptor agonist-only	99	2.65%
Total	Three mutually exclusive datasets	3,736	100.00%

3. Sensitivity analysis of annual publication output

3.1 Methods

The publication year (PY) was extracted from each of the three WoS text files and summarized for 2004–2025. Missing year-by-dataset combinations were set to zero. A stacked bar chart was constructed with Dataset 1 at the bottom, Dataset 3 in the middle, and Dataset 2 at the top; the value centered above each bar represents the total annual output across all three datasets.

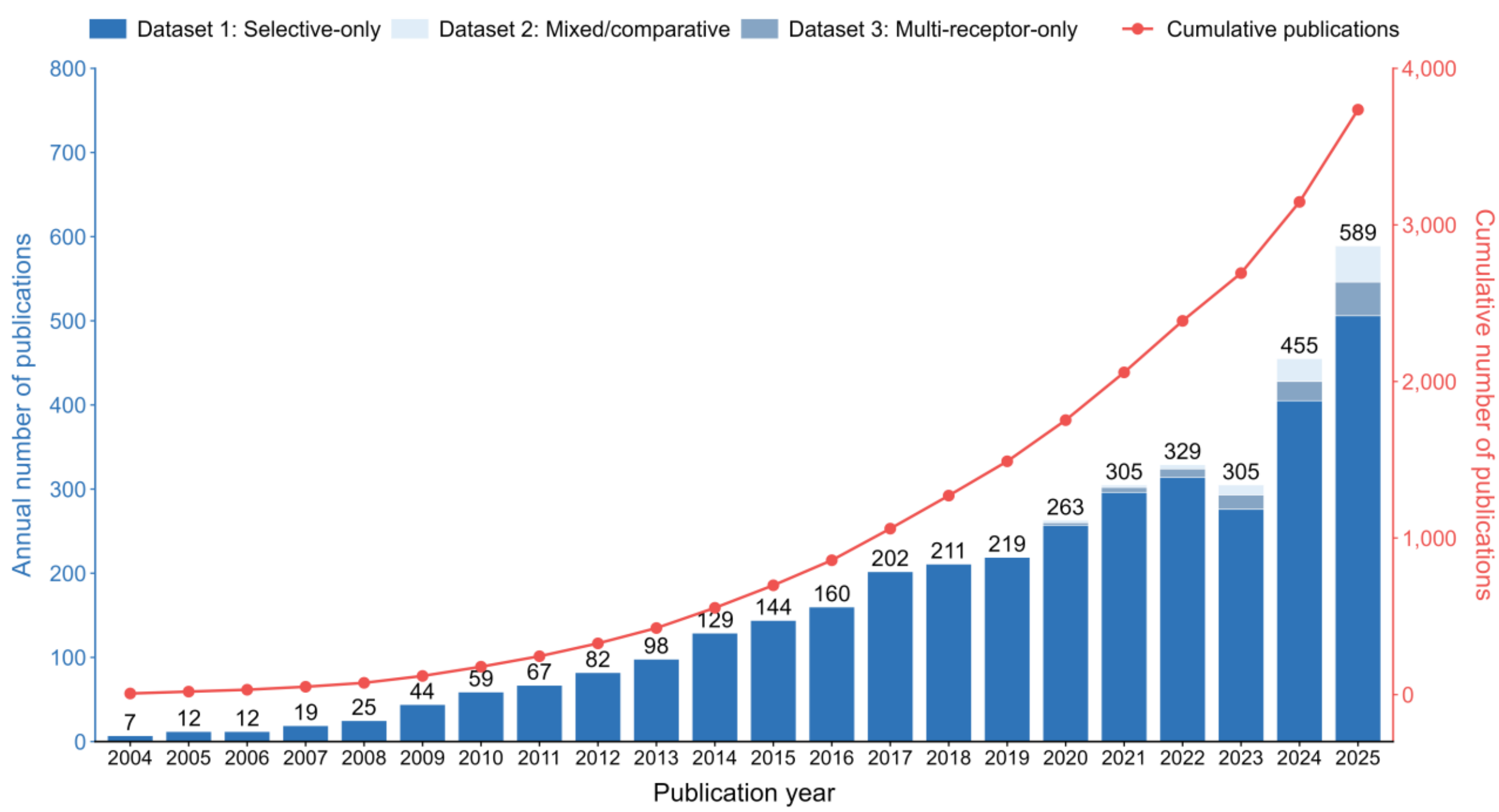

Fig 1. Annual publication output of the three mutually exclusive datasets, 2004–2025.

3.2 Results

Total annual output increased from 7 publications in 2004 to 589 in 2025. All records published during 2004–2019 belonged to Dataset 1; Datasets 2 and 3 first appeared in 2020. Together, the two newer categories contributed 6 articles in 2020 (2.28% of that year's output) and 83 articles in 2025 (14.09%), demonstrating their growing contribution to recent publication growth.

Dataset 1 nevertheless remained dominant throughout the study period. In 2025, Datasets 1, 2, and 3 contained 506, 43, and 40 publications, respectively; Dataset 1 accounted for 85.91% of that year's output. Total output reached 329 in 2022, decreased temporarily to 305 in 2023, and then rose to 455 in 2024 and 589 in 2025.

Year	Dataset 1	Dataset 3	Dataset 2	Annual total
2004	7	0	0	7
2019	219	0	0	219
2020	257	3	3	263
2022	314	10	5	329
2023	276	17	12	305
2024	405	23	27	455
2025	506	40	43	589

4. Stability of keyword occurrence and TLS rankings

4.1 Data and statistical methods

Top 100 keyword co-occurrence networks were generated in VOSviewer for Dataset 1, Dataset 1+2, and Dataset 1+2+3. Each item file contained the keyword label, cluster membership, occurrence count, and TLS. Occurrences measure keyword frequency, whereas TLS is the sum of the link strengths connecting a keyword to all other keywords in the network.

Within each network, keywords were ranked in descending order by occurrences and TLS. For each pair of networks, Spearman's rank correlation coefficient was calculated for keywords shared by both Top 100 lists; Top 100 and Top 10 overlap were also recorded. Kendall's coefficient of concordance (W) was calculated across the 90 keywords shared by all three networks. Average ranks were used for ties. All values reported below were taken from the R output workbook.

4.2 Results

Comparison	Shared keywords	Top 10 overlap	Occurrence ρ	TLS ρ
Dataset 1 vs Dataset 1+2	95	10/10 (100%)	0.998	0.995
Dataset 1 vs full dataset	91	10/10 (100%)	0.993	0.990
Dataset 1+2 vs full dataset	94	10/10 (100%)	0.992	0.993

All pairwise Spearman correlations were statistically significant ($p < 0.001$). Correlations ranged from 0.992 to 0.998 for occurrence ranks and from 0.990 to 0.995 for TLS ranks, indicating extremely high stability. Every occurrence- and TLS-based Top 10 list showed complete overlap. Across the 90 keywords common to all three networks, Kendall's W was 0.998 for occurrences and 0.996 for TLS (both $p < 0.001$).

The common Top 10 keyword set consisted of t2dm, glp-1 receptor agonists, liraglutide, glp-1, exenatide, semaglutide, glycemic control, sglt-2 inhibitors, obesity, and dulaglutide. The first four positions were identical across all datasets; only minor order changes occurred among exenatide and semaglutide and among obesity, sglt-2 inhibitors, and dulaglutide.

Interpretation. The Spearman and Kendall results indicate that the relative importance of the core keywords was highly robust to dataset definition.

5. Thematic cluster structure and distribution

5.1 Cluster alignment and agreement

VOSviewer generated cluster labels independently for each network; consequently, identically numbered clusters across files could not be assumed to represent the same theme. Cross-tabulations were created from shared keywords, and clusters in Dataset 1+2 and the full dataset were aligned to the seven Dataset 1 reference clusters by maximizing shared membership. The R implementation selected one maximum-overlap assignment using its deterministic optimization result, and this R-selected solution was used throughout the report.

Agreement between the independent partitions was evaluated using the adjusted Rand index (ARI), normalized mutual information (NMI), and the proportion of shared keywords assigned to corresponding themes after optimal alignment. Auxiliary p values for ARI were obtained from 100,000 permutations. ARI and NMI are invariant to the arbitrary numeric labels assigned to clusters.

Comparison	Clusters	ARI	NMI	Same aligned theme
Dataset 1 vs Dataset 1+2	7 vs 7	0.780	0.843	78/95 (82.1%)
Dataset 1 vs full dataset	7 vs 6	0.611	0.688	65/91 (71.4%)
Dataset 1+2 vs full dataset	7 vs 6	0.641	0.675	68/94 (72.3%)

Dataset 1 and Dataset 1+2 showed high agreement (ARI = 0.780; NMI = 0.843), with 82.1% of their shared keywords remaining in corresponding themes after alignment. Comparisons involving the full dataset yielded ARI values of 0.611–0.641 and NMI values of 0.675–0.688, with approximately 71%–72% of shared keywords retained in corresponding themes. These findings indicate moderate-to-high structural agreement, but greater reorganization after Dataset 3 was added. All ARI permutation p values were <0.001.

5.2 Theme interpretation and change in cluster count

Dataset 1 and Dataset 1+2 each contained seven clusters, whereas the full dataset contained six. Based on its high-occurrence keywords, the seven Dataset 1 reference clusters were summarized as follows.

Reference cluster	Indicative theme	Representative keywords
1	Cardiorenal outcomes and real-world treatment	GLP-1RAs; SGLT2 inhibitors; cardiovascular disease; chronic kidney disease; heart failure
2	Drugs and fundamental metabolic mechanisms	T2DM; liraglutide; GLP-1; exenatide; insulin resistance
3	Insulin combinations and economic evaluation	dulaglutide; cost-effectiveness; basal insulin; iGlarLixi; IDegLira
4	Obesity, weight loss, and cardiometabolic risk	obesity; HbA1c; weight loss; body weight; cardiovascular risk
5	Clinical trials, pharmacokinetics, and safety	incretin; randomized controlled trial; pharmacokinetics; safety
6	Glycemic management and real-world evidence	glycemic control; antidiabetic drugs; real-world evidence; insulin therapy
7	Semaglutide-related metabolic and inflammatory research	semaglutide; metformin; NAFLD; inflammation; gut microbiota

Under the R-selected alignment, Dataset 1 reference cluster 5 had no independent counterpart in the full dataset, whereas full-dataset cluster 5 aligned to Dataset 1 reference cluster 7. The keywords of the unrepresented reference theme were redistributed across other clusters. Tirzepatide entered the drug/mechanism cluster centered on T2DM, GLP-1, and insulin. The reduction from seven to six clusters

therefore represents consolidation or reorganization of thematic boundaries rather than disappearance of the field's principal subjects.

5.3 Test of the aligned theme-size distribution

After applying the R-selected optimal mapping, the three Top 100 keyword sets were classified into the seven Dataset 1 reference themes, forming a 3 × 7 contingency table. Because the full dataset contained only six clusters, the unmatched reference theme was assigned a count of zero. A chi-square homogeneity test was supplemented by 200,000 Monte Carlo replicates, and Cramér's V was calculated as an effect-size measure.

Statistic	R result
χ^2	19.572
Degrees of freedom	12
Asymptotic p value	0.076
Monte Carlo p value	0.075
Cramér's V	0.181
Minimum expected count	7.67

At $\alpha = 0.05$, the overall distribution of aligned theme sizes did not differ statistically across the three dataset definitions. The R estimate of Cramér's V was 0.181, indicating a small difference in the distribution. This supports relative stability in broad thematic composition.

Statistical limitation. The three analytical datasets are nested, and every network was restricted to its Top 100 keywords; the observations in the contingency table are therefore not strictly independent. The chi-square and Monte Carlo p values should be treated as supportive and descriptive. Cluster count, ARI/NMI, aligned agreement, and changes in thematic meaning should be considered jointly.

6. Overall conclusion

The core keyword rankings and network connectivity measures were highly robust to dataset definition. Whether the analysis was restricted to selective GLP-1RA studies or progressively expanded to include mixed/comparative and multi-receptor-only studies, the Top 10 keyword sets remained unchanged and every pairwise Spearman correlation exceeded 0.99.

At the thematic level, the findings are best characterized as overall stability with boundary reorganization. Dataset 1 and Dataset 1+2 showed high cluster agreement. After Dataset 3 was added, the number of clusters decreased from seven to six and some keywords were reassigned, but the R-based test did not identify a statistically significant difference in the overall aligned theme-size distribution. The principal bibliometric conclusions were therefore not dependent on modest changes in dataset scope, although multi-receptor research broadened and reorganized some recent thematic boundaries.

Appendix A. Annual publications in the three mutually exclusive datasets, 2004–2025

Year	Dataset 1	Dataset 3	Dataset 2	Total
2004	7	0	0	7
2005	12	0	0	12
2006	12	0	0	12
2007	19	0	0	19
2008	25	0	0	25
2009	44	0	0	44
2010	59	0	0	59
2011	67	0	0	67
2012	82	0	0	82
2013	98	0	0	98
2014	129	0	0	129
2015	144	0	0	144
2016	160	0	0	160
2017	202	0	0	202
2018	211	0	0	211
2019	219	0	0	219
2020	257	3	3	263
2021	296	6	3	305
2022	314	10	5	329
2023	276	17	12	305
2024	405	23	27	455
2025	506	40	43	589

Note: The appendix follows the visual stacking order used in Figure 1: Dataset 1 (bottom), Dataset 3 (middle), and Dataset 2 (top).

Appendix B. R-selected alignment and aligned cluster counts

The following mapping and counts reproduce the single maximum-overlap solution selected by the R analysis and used for the theme-size test in this report.

Expanded dataset	Original cluster → Dataset 1 reference cluster
Dataset 1+2	1→2; 2→1; 3→7; 4→5; 5→3; 6→4; 7→6
Full dataset	1→2; 2→1; 3→6; 4→3; 5→7; 6→4

Dataset	Cluster 1	Cluster 2	Cluster 3	Cluster 4	Cluster 5	Cluster 6	Cluster 7
Dataset 1	21	20	18	14	11	9	7
Dataset 1+2	21	24	12	9	12	9	13
Full dataset	20	24	14	13	0	16	13
