# Supplementary material for: Global Landscape and Thematic Evolution of Selective GLP‐1 Receptor Agonists and GLP‐1–Based Multi‐Receptor Agonists in Type 2 Diabetes Mellitus: A Bibliometric Analysis
Source: Endocrinol Diabetes Metab. 2026 Sep 30;9(6):e70351. doi: 10.1002/edm2.70351 (PMC13627873; doi:10.1002/edm2.70351)
Supplement: Supplementary file 2 [file EDM2-9-e70351-s005.pdf]

Eligibility Assessment Manual for Source Articles in a Bibliometric Study of Selective GLP-1 Receptor Agonists and GLP-1–Based Multi-Receptor Agonists in Type 2 Diabetes Mellitus

**Document status**	Frozen screening rules
**Effective date**	25 July 2026
**Applies to**	Two-stage independent rescreening of candidate source records
**Primary database**	Web of Science Core Collection candidate records
**Screening unit**	One bibliographic record
**Required reviewers**	Two independent reviewers; disagreements resolved after independent assessment

Contents

1. **Purpose and Governing Principles**
2. **Terminology and Units of Screening and Analysis**
3. **Overarching Eligibility Criteria**
4. **Sequential Screening Algorithm**
5. **Detailed Eligibility Rules**
6. **Original Secondary Analyses Versus Evidence Syntheses**
7. **Mixed-Population Studies**
8. **Animal, Cellular, and Mechanistic Studies**
9. **Exclusion Codes and the First Unmet Criterion Principle**
10. **Screening Workflow, Reviewer Independence, and Disagreement Resolution**

1. Purpose and Governing Principles

This manual is intended to guide the independent, auditable, and reproducible rescreening of candidate publications in order to establish the final source-document dataset that conforms to the predefined scope of the study. This dataset will serve as the basis for all bibliometric analyses conducted on the included publications, including analyses of annual scientific output, countries, institutions, authors, source journals, keywords, direct citation counts, and normalized citation rankings.

Core Principles
The designation "Article" in the Web of Science Core Collection is a bibliographic document-type label used solely to define the initial set of candidate records. This designation alone does not establish that a publication reports original research, nor does it demonstrate that T2DM and an eligible drug or drug class constitute substantive components of the study. Final inclusion must therefore be determined according to the content-based eligibility criteria specified in this manual.

Note:

1. The unit of screening is the candidate source-document record.
2. Cited references are not required to meet the eligibility criteria applied to source documents. Guidelines, consensus statements, reviews, and studies not specifically focused on T2DM may still form part of the intellectual knowledge base represented in the co-cited reference analysis.
3. Only one primary reason for exclusion should be recorded for each excluded publication. The reason must be assigned according to the predefined hierarchical order using the "first unmet criterion" principle.
4. When the title and abstract do not provide sufficient information to justify exclusion, the publication should proceed to full-text review.

2. Terminology and Units of Screening and Analysis

Term	Operational Definition
**Candidate record**	A bibliographic record that remains after completion of all database-level preprocessing steps, including search restrictions, deduplication, removal of retracted records, and exclusion of records with a verified final publication year of 2026, and that therefore enters title and abstract screening.
**Included source article**	A publication that satisfies all eligibility criteria specified in this manual and is included in the final bibliometric dataset.
**Cited reference**	A publication listed in the reference section of an included source article. Cited references are not required to satisfy the eligibility criteria applied to source articles.
**Original study**	A study that reports new findings derived from clinical, observational, experimental, case-based, pharmacovigilance, individual-level or record-level secondary analyses, or original analytical modelling.
**Full-text review**	Examination of the full-text methods and results when at least one key eligibility criterion cannot be assessed reliably from the title and abstract alone.
**Mixed population**	A study population that includes both participants with T2DM and participants without T2DM, or participants representing multiple types of diabetes or disease states.

Unit of Analysis:

The screening unit is one deduplicated bibliographic record. Multiple publications arising from the same trial, cohort, or study population may each be included when they report substantively distinct original analyses or findings. Duplicate reports of the same publication or the same results must not be counted more than once.

3. Overarching Eligibility Criteria

Operational Definition of an Eligible Source Article

An eligible source article must be a full-length research article reporting original experimental, clinical, observational, case-based, or model-based findings, or a new original analysis of previously defined trials, cohorts, databases, pharmacovigilance systems, or other identifiable data sources.

A selective glucagon-like peptide-1 receptor agonist (GLP-1RA), or a dual- or triple-receptor agonist with GLP-1 receptor agonist activity, must constitute a central intervention, exposure, comparator, target of efficacy or safety evaluation, or subject of mechanistic investigation. In addition, type 2 diabetes mellitus (T2DM) must represent a substantive component of the study population, experimental model, primary research question, or a subgroup for which the results can be interpreted independently.

3.1 All Four Eligibility Domains Must Be Satisfied

Domain	Required Criterion
**A. Study design**	The publication reports original research findings, an eligible original secondary analysis, or an eligible original model-based analysis.
**B. Drug scope**	The study investigates at least one selective GLP-1RA or a dual- or triple-receptor agonist with GLP-1 receptor agonist activity.
**C. Centrality of the eligible drug**	The eligible drug must be a central intervention, exposure, comparator, target of efficacy or safety evaluation, or subject of mechanistic investigation.
**D. Substantive relevance to T2DM**	T2DM must constitute a central component of the study population, experimental model, research question, or a subgroup for which results are independently interpretable.

3.2 Publication Forms Excluded from the Source-Article Corpus

1. Systematic reviews and meta-analyses, including network meta-analyses and individual participant data meta-analyses;
2. Narrative reviews, scoping reviews, and other literature-based reviews;
3. Clinical guidelines, consensus statements, position statements, and expert recommendations;
4. Editorials, commentaries, news items, and letters that do not report original data;
5. Study protocols, trial design papers, and statistical analysis plans that do not report study results;
6. Conference abstracts are excluded under E6 regardless of whether a corresponding full-length article exists.

4. Sequential Screening Algorithm

Step	Screening Question	Action at the Title and Abstract Stage	Exclusion Code / Reason for Full-Text Review
**1**	Does the publication report original research or an eligible original secondary analysis?	**No:** Exclude. **Uncertain:** Proceed to full-text review.	E1 / F1
**2**	Does the study drug fall within the predefined drug scope?	**No:** Exclude. **Uncertain:** Proceed to full-text review.	E2 / F2
**3**	Is the eligible drug a central subject of the study?	**No:** Exclude. **Uncertain:** Proceed to full-text review.	E2 / F2
**4**	Does T2DM constitute a substantive component of the study?	**No:** Exclude. **Uncertain:** Proceed to full-text review.	E3 / F3
**5**	For a mixed population, can the T2DM-specific results be identified and interpreted independently?	**No:** Exclude. **Uncertain:** Proceed to full-text review.	E4 / F4
**6**	For a non-human study, is the model explicitly relevant to T2DM?	**No:** Exclude. **Uncertain:** Proceed to full-text review.	E5 / F5
**7**	Does the publication report complete and assessable original results?	**No:** Exclude. **Uncertain:** Proceed to full-text review.	E6 / F6
**8**	Complete the title and abstract screening decision.	**Retain** if no definite exclusion criterion and no unresolved uncertainty are present. **Proceed to full-text review** if any criterion remains uncertain.	TA
**9**	Reapply Steps 1–7 at the full-text stage.	Make the final inclusion or exclusion decision. Exclude the publication if the full text remains unavailable or if the available information is insufficient to determine eligibility (E7).	E1–E7

First Unmet Criterion Principle

When a publication meets more than one potential exclusion condition, exclusion criteria E1–E6 are applied in the predefined hierarchical order. The first criterion that the publication clearly fails determines the single primary exclusion code. E7 is not part of the first-unmet-criterion sequence and is used only when eligibility cannot be determined after documented reasonable efforts have been made to obtain the full text or verify the essential information required for assessment.

At the title and abstract stage, an exclusion code from E1 to E6 should be assigned only when the corresponding exclusion criterion is clearly met. A publication must not be excluded on the basis of assumptions. When eligibility remains uncertain, the corresponding full-text review reason from F1 to F6 must be recorded, and the publication must proceed to full-text review.

For example:

1. *a meta-analysis of obesity studies that does not include a substantive T2DM component is assigned **E1**, because it first fails the originality criterion, rather than **E3**.*
2. *By contrast, an original randomized controlled trial of semaglutide conducted exclusively in participants with obesity and without T2DM satisfies the originality and drug-scope criteria but fails the T2DM substantive-relevance criterion. It is therefore assigned **E3**.*

5. Detailed Eligibility Rules

5.1 Preparation of the Candidate Dataset Before Content Screening

Before title and abstract screening, the candidate dataset must undergo the following preprocessing steps. Records removed at this stage are not considered exclusions arising from title and abstract screening or full-text assessment and must not be assigned an E1–E7 exclusion code. The number of records removed at each step should be reported separately in the study flow diagram.

1. Duplicate Records

Duplicate records must be systematically identified and removed before content-based screening. The Web of Science accession number (UT) should be used as the primary identifier. DOI, PMID, title, author information, and publication year should be used as secondary fields to verify potential duplicates. The number of duplicate records removed has been recorded and reported separately in the study flow diagram.

2. Records with a Verified Final Publication Year of 2026

Records with a verified final publication year of 2026 (PY = 2026) must be removed before content-based screening because they fall outside the predefined publication period. The number of records removed has been recorded and reported separately in the study flow diagram.

3. Retracted Articles and Retraction Notices

Retracted articles and retraction notices must be removed before content-based screening on the basis of the publication status recorded in Web of Science. These records have been recorded and reported separately in the study flow diagram and have not proceeded to title and abstract screening.

5.2 Originality of the Study

Decision	Eligible or Ineligible Study Types
**Include**	Randomized or non-randomized human intervention studies; observational and real-world studies; case reports and case series; animal, cellular, and mechanistic studies; pharmacovigilance studies; eligible post hoc, pooled, or secondary analyses; and original pharmacoeconomic, pharmacodynamic, pharmacokinetic, or other modelling analyses.
**Exclude**	Systematic reviews, conventional meta-analyses, network meta-analyses, individual participant data meta-analyses, narrative reviews, guidelines, consensus statements, position statements, editorials, commentaries.

5.3 Drug Scope and Centrality

Drug Category	Scope and Examples
**D1. Selective GLP-1 receptor agonists**	Examples include exenatide, liraglutide, lixisenatide, albiglutide, dulaglutide, semaglutide, and efpeglenatide.
**D2. Dual GIP/GLP-1 receptor agonists**	Examples include tirzepatide.
**D3. Dual GLP-1/glucagon receptor agonists**	Examples include mazdutide, cotadutide, and survodutide.
**D4. Triple GIP/GLP-1/glucagon receptor agonists**	Examples include retatrutide.

Note: A publication is not eligible when an in-scope drug is mentioned only in the introduction, discussion, Keywords Plus, or reference list and is not substantively investigated in the study. To meet the drug-centrality criterion, an eligible drug must serve in at least one of the following substantive roles:

1. a primary or secondary intervention;
2. a clinical exposure;
3. an active comparator;
4. the subject of an efficacy or safety evaluation;
5. the subject of a pharmacokinetic or pharmacodynamic evaluation; or
6. the experimental target of a mechanistic investigation.

5.4 T2DM as a Substantive Component

T2DM is considered a substantive component of a study when at least one of the following conditions is met:

1. All study participants, or a clearly defined study population, have T2DM.
2. A mixed population includes a distinct T2DM cohort or subgroup for which the results can be identified and interpreted independently.
3. The study uses an explicitly defined non-human model of T2DM, insulin resistance, impaired glucose tolerance, pancreatic β-cell injury or dysfunction, or a T2DM-related complication.
4. The research question explicitly evaluates the efficacy, safety, mechanism, use, outcomes, or treatment patterns of an eligible drug in the context of T2DM.

The following circumstances are not sufficient to establish substantive relevance to T2DM:

1. T2DM is mentioned only as background information.
2. T2DM is recorded only as a baseline comorbidity.
3. T2DM is used solely as an adjustment covariate.
4. T2DM is considered only as a future risk or potential outcome.
5. T2DM is discussed only as a possible future indication.
6. T2DM appears only through Keywords Plus indexing.

Studies conducted exclusively in participants with obesity or overweight who are explicitly described as not having T2DM must be excluded.

5.5 Complete Original Results

Publications that report only a study protocol, statistical analysis plan, study or trial design, trial registration, conference abstract, or baseline description without substantive study results should be assigned exclusion code E6.

6. Original Secondary Analyses Versus Evidence Syntheses

Core Distinction
The principal distinction lies in the unit of data being reanalyzed. Original secondary analyses examine individual-level or record-level data, such as data from patients, animals, biological samples, clinical cases, or database records. By contrast, meta-analyses and other forms of evidence synthesis primarily combine study-level estimates that have already been reported across multiple publications.

Feature	Eligible Original Secondary Analyses	Excluded Evidence Syntheses
**Unit of analysis**	Individual patients, animals, samples, cases, or database records	Study-level means, risk ratios, or other summary effect estimates
**Data source**	A predefined trial programme, cohort, database, registry, or pharmacovigilance system	Multiple studies identified through a systematic literature search and study-selection process
**Common terminology**	Post hoc analysis, secondary analysis, pooled participant-level analysis, integrated analysis	Systematic review, meta-analysis, network meta-analysis, individual participant data meta-analysis
**Eligibility**	Included when the other eligibility criteria are satisfied	Excluded under E1
**Typical analyses**	Subgroup analyses, predictor analyses, mediation analyses, and individual-level survival analyses	Forest plots, between-study heterogeneity analyses, publication-bias assessments, and study-level meta-regression

6.1 Examples Generally Eligible for Inclusion

1. Reanalysis of existing participant-level data from a cardiovascular outcome trial to evaluate efficacy and safety across renal-function subgroups.
2. Combination of participant-level data from a predefined set of trials within the SUSTAIN or SURPASS clinical development programme to assess whether age, sex, or baseline HbA1c modifies treatment response.
3. Patient-level analysis of an administrative claims database comparing treatment persistence between semaglutide and dulaglutide.
4. Signal-detection analysis based on individual adverse-event reports from the FDA Adverse Event Reporting System, provided that the T2DM indication forms a substantive part of the analysis.

6.2 Examples Excluded as Evidence Syntheses

1. Identification of multiple randomized controlled trials through a literature search, followed by extraction of published HbA1c means and standard deviations and construction of a forest plot.
2. Meta-regression using the mean age reported by each included study.
3. An analysis that obtains participant-level data from multiple independent studies through a systematic search and study-selection process and is explicitly described as an individual participant data meta-analysis.

Pooled Analyses and Individual Participant Data Meta-Analyses

The term “pooled” does not, by itself, determine eligibility. A combined individual-level analysis of trials from a predefined clinical development programme, or of records drawn from the same predefined database or data system, is generally considered an original secondary analysis and may be included when the remaining eligibility criteria are met. In contrast, an individual participant data meta-analysis assembled through a systematic literature search, formal study selection, and risk-of-bias assessment remains a form of evidence synthesis and is therefore excluded under E1.

7. Mixed-Population Studies

Mixed-population studies are eligible only when the T2DM population is explicitly defined and at least one T2DM-specific outcome, effect estimate, model coefficient, or stratified result relevant to the research question can be identified and interpreted independently.

7.1 Evidence Supporting Independent Interpretation of T2DM-Specific Results

1. The sample size of the T2DM group is reported separately.
2. Means, event rates, effect estimates, or confidence intervals are reported separately for the T2DM group.
3. Stratified tables, figures, or model estimates are presented according to T2DM status.
4. An interaction with diabetes status is reported and allows the effect in the T2DM group to be interpreted.
5. The T2DM cohort or subgroup is clearly defined in both the Methods and Results sections.

7.2 Evidence Considered Insufficient

1. T2DM appears only in the baseline characteristics table.
2. T2DM is included only as an adjustment covariate.
3. The article states that some participants had diabetes but does not report their number or provide diabetes-specific results.
4. Results for participants with and without T2DM are fully combined and cannot be separated.
5. Potential relevance to T2DM is suggested only in the Discussion section.

Example	Decision
A study of participants with obesity includes individuals with T2DM and reports results separately according to diabetes status.	Include
A study of participants with obesity includes individuals with T2DM, but all results are pooled across diabetes-status groups.	Exclude, E4
A study includes participants with T1DM and T2DM and reports the T2DM results separately.	Include
In a mixed CKD or CVD population, T2DM is included only as an adjustment covariate.	Exclude, E3

8. Animal, Cellular, and Mechanistic Studies

Non-human original studies are eligible for inclusion when they meet the criteria specified below. Their inclusion helps preserve a comprehensive view of the research landscape, extending from basic mechanisms and drug development to clinical translation. However, a non-human study is not considered eligible solely because it contains GLP-1-related terminology or examines an obesity-related phenotype.

8.1 Animal Studies

An animal study is eligible when all of the following conditions are met:

1. An eligible drug is a central experimental intervention.
2. The study uses a clearly defined model of T2DM or a metabolic model directly relevant to T2DM.
3. The research question directly concerns the treatment, mechanisms, or complications of T2DM.
4. The publication reports original experimental methods and results.

Eligible models may include:

1. genetic models of T2DM;
2. pharmacologically or diet-induced models of T2DM;
3. clearly defined models of insulin resistance or impaired glucose tolerance;
4. models of pancreatic β-cell dysfunction; and
5. models of T2DM-related cardiovascular, renal, hepatic, neurological, or other complications.

Note: Studies conducted in otherwise healthy animals that examine only food intake, general weight loss, or another outcome without a clearly defined T2DM-related research question are excluded under E5.

8.2 Cellular and In Vitro Studies

Cellular or in vitro studies are eligible when they examine a pathological condition or mechanism directly relevant to T2DM, such as:

1. pancreatic β-cell dysfunction;
2. glucotoxicity or lipotoxicity;
3. high-glucose-induced injury;
4. insulin resistance; or
5. cellular injury associated with a T2DM-related complication.

Note:

1. An eligible drug should serve as a central experimental intervention or as the principal subject of the mechanistic investigation.
2. General receptor-binding studies or broadly applicable signalling studies without a clearly defined T2DM-related question are excluded under **E5**.

9. Exclusion Codes and the First Unmet Criterion Principle

Code	Primary Reason for Exclusion	Operational Guidance
**E1**	**Not an original study**	Reviews, meta-analyses, guidelines, consensus statements, editorials, and other publications that do not report original data.
**E2**	**Drug outside the predefined scope or not a central study subject**	The study does not investigate an eligible drug, or the eligible drug is mentioned only incidentally and does not serve as a substantive intervention, exposure, comparator, evaluation target, or mechanistic subject.
**E3**	**T2DM is not a substantive component**	Studies focused exclusively on obesity, healthy populations, or other diseases without independently identifiable or substantively interpretable T2DM-related content.
**E4**	**T2DM-specific results are not independently interpretable in a mixed population**	The study includes participants with T2DM, but the T2DM-specific findings cannot be separated from the overall results or interpreted independently.
**E5**	**Non-human model is not sufficiently relevant to T2DM**	Studies using healthy animals, general weight-loss models, or broadly applicable cellular or mechanistic models without a clearly defined T2DM-related context.
**E6**	**Complete original results are not reported**	Study protocols, design papers, conference abstracts, statistical analysis plans, or other records that do not provide assessable original results.
**E7**	**Full text unavailable or information insufficient**	Eligibility cannot be determined reliably after reasonable efforts to obtain the full text and verify the information required for assessment.

Application of the First Unmet Criterion Principle

For the operational application of the first unmet criterion principle during title and abstract screening and full-text assessment, see Section 4, “Sequential Screening Algorithm.”

10. Screening Workflow, Reviewer Independence, and Disagreement Resolution

1. Database-level restrictions, deduplication, removal of retracted records, and removal of records assigned to a final publication year of 2026 (PY = 2026) are completed before content screening. The candidate dataset entering content screening is then finalized and frozen, while the original Web of Science export files are retained for documentation and verification.
2. A screening manual is developed to define the eligibility criteria, decision sequence, exclusion codes, and operational procedures.
3. Before formal screening, the two reviewers independently pilot-screen the same set of approximately 50–100 potentially ambiguous or borderline records. Their decisions are then compared and discussed to calibrate the interpretation and application of the screening rules. All pilot records are re-screened independently after the rules have been finalized and are included in the formal screening dataset.
4. After completion of the pilot screening and resolution of any interpretive issues, the screening rules are finalized and remain unchanged throughout the formal screening process.
5. The first stage consists of title and abstract screening. Each reviewer independently records the following:
    - TA_Decision: Retain, Exclude, or Full-text review;
    - TA_Exclusion_Code: E1–E6 when the record is excluded;
    - Full_Text_Reason: F1–F6 when full-text review is required; and
    - TA_Note: completed only for borderline cases, unusual circumstances, or decisions requiring additional explanation.
6. After both reviewers have independently completed title and abstract screening for all candidate records, their decisions are merged and compared. A record proceeds to full-text review when either reviewer selects Full-text review or when the two reviewers reach different title and abstract decisions.
7. The second stage consists of full-text assessment. The full-text decision is recorded in addition to, rather than replacing, the title and abstract screening record:
    - FT_Decision: Include or Exclude;
    - FT_Exclusion_Code: E1–E7 when the article is excluded; and
    - FT_Decision_Basis: a concise explanation of the evidence supporting the final full-text decision for every article assessed at this stage.
8. Disagreements are resolved through discussion between the two reviewers. When consensus cannot be reached, a third reviewer adjudicates the decision. This process is intended to promote consistent application of the eligibility criteria and reduce the risk of selection bias.
