# Supplementary material for: Global Landscape and Thematic Evolution of Selective GLP‐1 Receptor Agonists and GLP‐1–Based Multi‐Receptor Agonists in Type 2 Diabetes Mellitus: A Bibliometric Analysis
Source: Endocrinol Diabetes Metab. 2026 Sep 30;9(6):e70351. doi: 10.1002/edm2.70351 (PMC13627873; doi:10.1002/edm2.70351)
Supplement: Supplementary file 3 [file EDM2-9-e70351-s002.docx]

**Supplementary Methods**

**Data processing and analytical definitions**

Custom statistical calculations and most data processing were performed in R (version 4.5.2) using standardized Web of Science Core Collection (WoSCC) records and exported bibliometric results. Python was used only for the initial rule-based identification of records requiring review in the dataset-scope classification. Network occurrence, total link strength (TLS), and cluster assignments were generated by the software specified in the main Methods and imported into R only when required for sensitivity analyses.

**Entity extraction and counting**

Countries and institutions were derived from the standardized affiliation-address field (C1), and authors were derived from the standardized author field (AF). Repeated appearances of the same standardized entity within one article were removed before counting. Let $\delta_{ij}=1$ if entity $i$ occurred in article $j$ and $\delta_{ij}=0$ otherwise. The full-count output of entity $i$ was

$$D_{i}=\sum_{j=1}^{N} \delta_{ij}.$$

For fractional counting, let $k_{j}=\sum_{i} \delta_{ij}$ be the number of distinct entities of the relevant type in article $j$. The contribution of article $j$ to entity $i$ was

$$w_{ij}=\frac{\delta_{ij}}{k_{j}},$$

and the fractional output of entity $i$ was

$$F_{i}=\sum_{j=1}^{N} w_{ij}.$$

Thus, each article contributed a total weight of one across all valid countries, institutions, or authors represented in that article. Records without usable data for a given entity field were excluded only from analyses requiring that field.

**Corresponding-author country indicators**

Single-country publications (SCP) and multiple-country publications (MCP) were derived from the corresponding-author country output. For country $c$, the corresponding-author article count was

$$A_{c}=\mathrm{SCP}_{c}+\mathrm{MCP}_{c},$$

and the proportion involving international collaboration, denoted by $R_{c}$, was

$$R_{c}=\frac{\mathrm{MCP}_{c}}{A_{c}}.$$

An article was classified as SCP when all identified affiliations were from one country and as MCP when affiliations from at least two countries were present.

**Output shares and concentration measures**

For a given entity type, the fractional output share of entity $i$ was

$$p_{i}=\frac{F_{i}}{\sum_{r=1}^{n} F_{r}},$$

where $n$ is the number of entities with valid fractional output. With shares ordered as $p_{\left[ 1 \right]}\geq p_{\left[ 2 \right]}\geq\cdots\geq p_{\left[ n \right]}$, the share of the ten largest contributors was

$$\mathrm{CR}_{10}=\sum_{i=1}^{10} p_{\left[ i \right]}.$$

The Herfindahl–Hirschman Index (HHI) was

$$HHI=\sum_{i=1}^{n} p_{i}^{2}.$$

Higher CR10 and HHI values indicate greater concentration. For the Gini coefficient, fractional outputs were ordered in ascending order, $F_{\left( 1 \right)}\leq F_{\left( 2 \right)}\leq\cdots\leq F_{\left( n \right)}$, and

$$G=\frac{2\sum_{i=1}^{n} iF_{\left( i \right)}}{n\sum_{i=1}^{n} F_{\left( i \right)}}-\frac{n+1}{n}.$$

The corresponding Lorenz curve used the cumulative coordinates

$$L_{q}=\left( \frac{q}{n},\frac{\sum_{i=1}^{q} F_{\left( i \right)}}{\sum_{i=1}^{n} F_{\left( i \right)}} \right), q=0,1,\ldots,n.$$

A Gini coefficient of 0 denotes equality, whereas values approaching 1 indicate increasing inequality.

**Dataset-specific author h-index**

For each author, citations to that author’s included publications were sorted as $c_{\left( 1 \right)}\geq c_{\left( 2 \right)}\geq\cdots\geq c_{\left( m \right)}$. The dataset-specific h-index was

$$h=max\left\{ q:c_{\left( q \right)}\geq q \right\}.$$

This measure was calculated only from publications in the study corpus and therefore does not represent an author’s lifetime h-index. Self-citations were retained.

**Keyword-frequency normalization**

For the temporal heatmap, $x_{kt}$ denoted the number of included articles published in year $t$ that contained standardized keyword $k$; a keyword was counted at most once per article. For each retained keyword with a nonzero annual range, annual frequencies were independently rescaled to the interval 0–1:

$$z_{kt}=\frac{x_{kt}-\min_{t}\left( x_{kt} \right)}{\max_{t}\left( x_{kt} \right)-\min_{t}\left( x_{kt} \right)}.$$

The transformation preserves the temporal profile of each keyword but does not make normalized values directly comparable as absolute frequencies across different keywords.

**Citation normalization and citation-ranking sensitivity**

Let $TC_{i}$ be the total citations of article $i$, and let $\overline{TC}_{y\left( i \right)}$ be the mean total citations of all eligible articles published in the same year. The year-normalized citation score was

$$\mathrm{YNCS}_{i}=\frac{TC_{i}}{\overline{TC}_{y\left( i \right)}}.$$

The primary normalized ranking was restricted to publications from 2004–2024. Publications from 2025 were summarized separately using total citations because of their short citation window. For the annual citation-distribution plot, the mean, median, and interquartile range were calculated within publication year, and displayed citation values were transformed as

$$y^{*}=log\left( 1+y \right).$$

As a sensitivity analysis, citations were also converted to within-year Hazen percentiles. For an article with within-year citation midrank $r_{iy}$ among $n_{y}$ eligible articles in year $y$,

$$H_{iy}=100\left( \frac{r_{iy}-0.5}{n_{y}} \right).$$

Ranks were assigned in ascending citation order so that higher percentiles indicated greater citation impact; tied citation counts received their average rank. Agreement between rankings based on YNCS and Hazen percentiles was assessed using Top-10 overlap and Spearman’s rank correlation.

**Dataset-scope classification and extraction**

Dataset-scope classification combined automated dictionary-based screening with manual review. Prespecified dictionaries for selective GLP-1RAs and GLP-1–based multi-receptor agonists were matched case-insensitively across the TI, AB, DE, and ID fields using alphanumeric boundaries. Records without a term from the GLP-1–based multi-receptor agonist dictionary were provisionally assigned to Dataset 1, whereas records containing such a term underwent manual title-and-abstract review. The flagged records were classified according to whether selective GLP-1RAs and/or GLP-1–based multi-receptor agonists constituted substantive study objects rather than background or indexing mentions, producing three mutually exclusive datasets: Dataset 1 (selective-only), Dataset 2 (mixed/comparative), and Dataset 3 (multi-receptor-only).

Final classifications were matched to the WoS plain-text records using normalized UT, with normalized DOI used as a fallback. Quality checks ensured successful and unambiguous matching, absence of cross-dataset duplication, and complete coverage of the source records. Detailed drug dictionaries, classification criteria, record counts, and quality-control procedures are provided in Supplementary File S5.

**R-based robustness calculations**

For two ranked results with Top-$K$ sets $A_{K}$ and $B_{K}$, overlap was calculated as

$$O_{K}=\frac{\left| A_{K}\cap B_{K} \right|}{K}.$$

Spearman’s rank correlation was calculated as the Pearson correlation between the corresponding rank vectors,

$$\rho_{s}=cor\left( R_{X},R_{Y} \right),$$

with average ranks assigned to ties. K = 10 was used for all ranking comparisons, and K = 100 was additionally used in the dataset-scope analysis. These measures were used in the citation-ranking, network-threshold, and dataset-scope sensitivity analyses. Network occurrence and TLS values used in these comparisons were imported from the software output files.

For cross-database sensitivity, coverage of a tie-inclusive highly cited set was calculated as

$$\mathrm{Coverage}_{K}=\frac{M_{K}}{N_{K}}\times100\%,$$

where $N_{K}$ is the number of articles in the tie-inclusive set defined by the citation threshold at rank $K$ that had a valid DOI and/or PMID, and $M_{K}$ is the number of those articles matched in PubMed.

For dataset-scope sensitivity, occurrence and TLS ranks were compared using overlap statistics, Spearman’s rank correlation, and Kendall’s coefficient of concordance. For the three-way comparison, Kendall’s W was calculated with correction for tied ranks as

$$W=\frac{12S}{m^{2}\left( n^{3}-n \right)-m\sum_{j=1}^{m} T_{j}},$$

where $m$ was the number of networks ($m$ = 3), $n$ was the number of keywords shared by all three networks ($n$ = 90), $S$ was the sum of squared deviations of keyword rank sums from their mean, and $T_{j}$ was the tie-correction term for network $j$.

Statistical significance was evaluated using the chi-square approximation,

$$\chi^{2}=m\left( n-1 \right)W, df=n-1.$$

Cluster agreement was evaluated from the cross-tabulation of shared-keyword memberships. If $n_{gh}$ denotes the number of shared keywords assigned to cluster $g$ in one solution and cluster $h$ in another, with row totals $a_{g}$, column totals $b_{h}$, and total $n$, then

$$E=\frac{\left[ \sum_{g} \binom{a_{g}}{2} \right]\left[ \sum_{h} \binom{b_{h}}{2} \right]}{\binom{n}{2}},$$

and the adjusted Rand index was

$$ARI=\frac{\sum_{g,h} \binom{n_{gh}}{2}-E}{\frac{1}{2}\left[ \sum_{g} \binom{a_{g}}{2}+\sum_{h} \binom{b_{h}}{2} \right]-E}.$$

Normalized mutual information (NMI) was calculated from the same shared-keyword cluster contingency table. Let each cell contain the number of shared keywords assigned to one cluster in solution A and one cluster in solution B, and let n be the total number of shared keywords. The joint and marginal proportions were defined as follows:

$$p_{gh}=\frac{n_{gh}}{n}, p_{g}=\frac{n_{g\cdot}}{n}, p_{h}=\frac{n_{\cdot h}}{n}$$

$$I\left( A;B \right)=\sum_{g,h:p_{gh}>0} p_{gh}\log\left( \frac{p_{gh}}{p_{g}p_{h}} \right)$$

$$H\left( A \right)=-\sum_{g:p_{g}>0} p_{g}\log p_{g}, H\left( B \right)=-\sum_{h:p_{h}>0} p_{h}\log p_{h}$$

$$\mathrm{NMI}\left( A,B \right)=\frac{2I\left( A;B \right)}{H\left( A \right)+H\left( B \right)}$$

In these expressions, the cell counts and their row and column totals were divided by n to obtain the joint and marginal proportions; I(A;B) is the mutual information, and H(A) and H(B) are the cluster-label entropies. Natural logarithms were used; any consistent logarithm base gives the same normalized value. If H(A) + H(B) equaled zero, NMI was set to 1. Both ARI and NMI are invariant to arbitrary cluster labels. For theme-level presentation, cluster labels were aligned with the Hungarian algorithm to maximize shared keyword membership; this alignment did not alter ARI or NMI. The statistical significance of ARI was assessed using 100,000 label permutations, with the permutation P value calculated as (b + 1)/(B + 1), where b was the number of permuted ARI values at least as large as the observed value and B was the number of permutations. Differences in aligned theme sizes were evaluated by a chi-square test with 200,000 Monte Carlo replicates, and effect size was summarized using Cramer’s V:

$$V=\sqrt{\frac{\chi^{2}}{N_{tab}\min\left( r-1,c-1 \right)}},$$

where $N_{tab}$ is the total frequency in the contingency table, and $r$ and $c$ are the numbers of rows and columns in the contingency table. Dataset classification and the complete dataset-scope sensitivity workflow are reported in Supplementary File S5. The corresponding reproducible scripts are provided in the Data and Code package.
