# Supplementary material for: Global Landscape and Thematic Evolution of Selective GLP‐1 Receptor Agonists and GLP‐1–Based Multi‐Receptor Agonists in Type 2 Diabetes Mellitus: A Bibliometric Analysis
Source: Endocrinol Diabetes Metab. 2026 Sep 30;9(6):e70351. doi: 10.1002/edm2.70351 (PMC13627873; doi:10.1002/edm2.70351)
Supplement: Supplementary file 4 [file EDM2-9-e70351-s004.docx]

**Supplementary figures and tables**

**Fig. S1 Annual citation distributions of included publications in GLP-1–based agonist research on T2DM, 2004–2025.** *Red circles and solid lines indicate mean citations per publication; blue triangles and dashed lines indicate median citations; and shading indicates the interquartile range. The y-axis is shown on a log1p-transformed scale to accommodate the strongly right-skewed citation distributions. Publications from 2025 were excluded from the primary year-normalized ranking because of their short citation window.*

***Table S1. Parameters used for constructing and visualizing bibliometric networks in VOSviewer.***

| **Network** | **Analysis type** | **Unit of analysis** | **Normalization method** | **Counting method** | **Threshold** | **Items meeting threshold** | **Items selected** | **Largest component** | **Attraction** | **Repulsion** | **Resolution** | **Min. cluster size** |
| --- | --- | --- | --- | --- | --- | --- | --- | --- | --- | --- | --- | --- |
| Institution collaboration network | Co-authorship | Organizations | Association strength | Full counting | ≥35 documents per organization | 38 | 38 | 38 | 2 | 1 | 1 | 1 |
| Country collaboration network | Co-authorship | Countries | Association strength | Full counting | ≥10 documents per country | 45 | 45 | 45 | 2 | 1 | 1 | 1 |
| Journal citation network | Citation | Sources | Association strength | Full counting | ≥10 documents per source | 68 | 68 | 68 | 2 | 1 | 1 | 1 |
| Journal co-citation network | Co-citation | Cited sources | Association strength | Full counting | ≥100 citations per cited source | 168 | 168 | 168 | 2 | 1 | 1 | 1 |
| Author collaboration network | Co-authorship | Authors | Association strength | Full counting | ≥15 documents per author | 83 | 83 | 82 | 2 | −1 | 1 | 1 |
| Author-keyword co-occurrence network | Co-occurrence | Author keywords | Association strength | Full counting | ≥9 occurrences per author keyword | 149 | 100 | 100 | 2 | 0 | 1 | 1 |

**Note：***** *For citation analysis, VOSviewer fixes the counting method as full counting; fractional counting is unavailable. All threshold-eligible items were selected, except for the author-keyword co-occurrence network, for which the 100 keywords with the highest relevance scores were selected from 149 eligible keywords. Only the largest connected component was visualized; items outside this component were retained in the corresponding descriptive statistics.*

***Table S2. PubMed coverage of the most-cited eligible WoSCC articles using tie-inclusive citation thresholds***

| Highly cited WoS subset | Citation cutoff (TC) | Articles with a valid DOI and/or PMID, n | Matched in PubMed, n | Coverage, % |
| --- | --- | --- | --- | --- |
| Top 100 | 248 | 101 | 99 | 98.02 |
| Top 200 | 143 | 204 | 198 | 97.06 |
| Top 300 | 109 | 303 | 294 | 97.03 |

**Note:** *Eligible articles identified by the independent PubMed search were matched to the top 100, 200, and 300 most-cited eligible WoSCC articles using DOI and/or PMID. All articles tied at each total-citation (TC) cutoff were included, so the actual subset size could exceed the nominal size. Coverage was calculated among WoSCC articles with a valid DOI and/or PMID.*

***Table S3. Threshold sensitivity analysis of the bibliometric networks***

| Network | Threshold level | Threshold criterion | Items meeting threshold | No. of clusters | Top 10 overlap | Spearman’s ρ | Brief assessment |
| --- | --- | --- | --- | --- | --- | --- | --- |
| Institutions | Lower | 0.8×T; ≥28 documents | 59 | 8 | 10/10(100%) | 0.988 | The Top 10 institutions were fully retained across thresholds. Rankings were highly stable at the lower threshold, while the higher threshold produced somewhat greater rank changes and fewer clusters. |
|  | Main | 1.0×T; ≥35 documents | 38 | 7 | Reference | Reference |  |
|  | Higher | 1.2×T; ≥42 documents | 23 | 5 | 10/10(100%) | 0.915 |  |
| Countries | Lower | 0.8×T; ≥8 documents | 48 | 5 | 10/10(100%) | 1 | The Top 10 countries were fully retained across thresholds. Their rankings were unchanged at the lower threshold and showed only a minor interchange at the higher threshold, while the cluster structure remained stable. |
|  | Main | 1.0×T; ≥10 documents | 45 | 5 | Reference | Reference |  |
|  | Higher | 1.2×T; ≥12 documents | 43 | 5 | 10/10(100%) | 0.988 |  |
| Journals | Lower | 0.8×T; ≥8 documents | 103 | 8 | 10/10(100%) | 1 | The Top 10 journals were fully retained across thresholds. Their rankings were unchanged at the lower threshold and showed only a minor interchange at the higher threshold; the cluster count decreased slightly at the higher threshold. |
|  | Main | 1.0×T; ≥10 documents | 68 | 8 | Reference | Reference |  |
|  | Higher | 1.2×T; ≥12 documents | 65 | 7 | 10/10(100%) | 0.988 |  |
| Co-cited journals | Lower | 0.8×T; ≥80 citations | 198 | 3 | 10/10(100%) | 1 | The composition and rankings of the Top 10 co-cited journals were unchanged across thresholds. Only the higher threshold produced one additional cluster, indicating minor sensitivity in the broader network structure. |
|  | Main | 1.0×T; ≥100 citations | 168 | 3 | Reference | Reference |  |
|  | Higher | 1.2×T; ≥120 citations | 148 | 4 | 10/10(100%) | 1 |  |
| Authors | Lower | 0.8×T; ≥12 documents | 127 | 13 | 9/10 (90%) | 0.909 | Most leading authors remained identifiable across thresholds, but Top 10 membership, rankings, and cluster partitioning showed greater threshold sensitivity than those of the other networks. |
|  | Main | 1.0×T; ≥15 documents | 83 | 10 | Reference | Reference |  |
|  | Higher | 1.2×T; ≥18 documents | 56 | 9 | 8/10 (80%) | 0.867 |  |
| Author keywords | Lower | 0.8×T; ≥7 occurrences | 196 | 12 | 10/10(100%) | 1 | The Top 10 author keywords and their rankings were unchanged across thresholds. Changes in the number of clusters were confined mainly to the broader set of lower-frequency keywords. |
|  | Main | 1.0×T; ≥9 occurrences | 149 | 9 | Reference | Reference |  |
|  | Higher | 1.2×T; ≥11 occurrences | 119 | 9 | 10/10(100%) | 1 |  |

**Note:** *For the lower and higher thresholds, top 10 overlap and Spearman’s ρ were calculated relative to the main-threshold ranking based on total link strength (TLS). T denotes the threshold used in the main analysis.*

***Table S4. Top 10 included publications from the immature 2025 citation cohort, ranked by total citations***

| Rank | First author | Year | Article title | Journal | Total citations |
| --- | --- | --- | --- | --- | --- |
| 1 | McGuire, Darren K. | 2025 | Oral semaglutide and cardiovascular outcomes in high-risk type 2 diabetes | New England Journal of Medicine | 294 |
| 2 | Rodriguez, Patricia J. | 2025 | Discontinuation and reinitiation of dual-labeled GLP-1 receptor agonists among US adults with overweight or obesity | JAMA Network Open | 248 |
| 3 | Bonaca, Marc P. | 2025 | Semaglutide and walking capacity in people with symptomatic peripheral artery disease and type 2 diabetes (STRIDE): a phase 3b, double-blind, randomised, placebo-controlled trial | The Lancet | 114 |
| 4 | Nicholls, Stephen J. | 2025 | Cardiovascular outcomes with tirzepatide versus dulaglutide in type 2 diabetes | New England Journal of Medicine | 99 |
| 5 | Davies, Melanie J. | 2025 | Cagrilintide-semaglutide in adults with overweight or obesity and type 2 diabetes | New England Journal of Medicine | 95 |
| 6 | Chou, Chien-Chih | 2025 | Association between semaglutide and nonarteritic anterior ischemic optic neuropathy: a multinational population-based study | Ophthalmology | 82 |
| 6 | Rosenstock, Julio | 2025 | Orforglipron, an oral small-molecule GLP-1 receptor agonist, in early type 2 diabetes | New England Journal of Medicine | 82 |
| 8 | Harrison, Stephen A. | 2025 | Safety and efficacy of efruxifermin in combination with a GLP-1 receptor agonist in patients with NASH/MASH and type 2 diabetes in a randomized phase 2 study | Clinical Gastroenterology and Hepatology | 80 |
| 9 | Sattar, Naveed | 2025 | Tirzepatide and muscle composition changes in people with type 2 diabetes (SURPASS-3 MRI): a post-hoc analysis of a randomised, open-label, parallel-group, phase 3 trial | The Lancet Diabetes & Endocrinology | 74 |
| 10 | Cai, Cindy X. | 2025 | Semaglutide and nonarteritic anterior ischemic optic neuropathy | JAMA Ophthalmology | 72 |

**Note:** *Publications are ranked descriptively by total citations. The 2025 cohort was excluded from the primary year-normalized ranking because of its short citation window. Citation counts were retrieved from WoSCC on July 26, 2026.*

***Table S5. Sensitivity comparison of the Top 10 included publications identified using mean-based year-normalized citation scores and within-year Hazen citation percentiles, 2004–2024***

| Article | Year | Total citations | Year-normalized score | Mean-based rank | Citation percentile | Percentile-based rank | Rank difference | Mean-based Top 10 | Percentile-based Top 10 |
| --- | --- | --- | --- | --- | --- | --- | --- | --- | --- |
| Marso, Steven P. (2016). Liraglutide and cardiovascular outcomes in type 2 diabetes | 2016 | 6,891 | 62.94 | 1 | 99.69 | 9 | 8 | Yes | Yes |
| Perkovic, Vlado (2024). Effects of semaglutide on chronic kidney disease in patients with type 2 diabetes | 2024 | 1,393 | 61.54 | 2 | 99.89 | 1 | -1 | Yes | Yes |
| Gerstein, Hertzel C. (2019). Dulaglutide and cardiovascular outcomes in type 2 diabetes (REWIND): a double-blind, randomised placebo-controlled trial | 2019 | 2,551 | 40.99 | 3 | 99.77 | 6 | 3 | Yes | Yes |
| Frias, Juan P. (2021). Tirzepatide versus semaglutide once weekly in patients with type 2 diabetes | 2021 | 1,564 | 34.00 | 4 | 99.84 | 3 | -1 | Yes | Yes |
| Pfeffer, Marc A. (2015). Lixisenatide in patients with type 2 diabetes and acute coronary syndrome | 2015 | 2,007 | 30.90 | 5 | 99.65 | 11 | 6 | Yes | No |
| Holman, Rury R. (2017). Effects of once-weekly exenatide on cardiovascular outcomes in type 2 diabetes | 2017 | 1,754 | 30.79 | 6 | 99.75 | 8 | 2 | Yes | Yes |
| Hernandez, Adrian F. (2018). Albiglutide and cardiovascular outcomes in patients with type 2 diabetes and cardiovascular disease (Harmony Outcomes): a double-blind, randomised placebo-controlled trial | 2018 | 1,506 | 26.14 | 7 | 99.76 | 7 | 0 | Yes | Yes |
| Garvey, W. Timothy (2023). Tirzepatide once weekly for the treatment of obesity in people with type 2 diabetes (SURMOUNT-2): a double-blind, randomised, multicentre, placebo-controlled, phase 3 trial | 2023 | 638 | 25.84 | 8 | 99.84 | 3 | -5 | Yes | Yes |
| Kosiborod, M. N. (2024). Semaglutide in patients with obesity-related heart failure and type 2 diabetes | 2024 | 567 | 25.05 | 9 | 99.67 | 10 | 1 | Yes | Yes |
| Davies, Melanie (2021). Semaglutide 2.4 mg once a week in adults with overweight or obesity, and type 2 diabetes (STEP 2): a randomised, double-blind, double-dummy, placebo-controlled, phase 3 trial | 2021 | 1,149 | 24.98 | 10 | 99.51 | 14 | 4 | Yes | No |
| Dahl, Dominik (2022). Effect of subcutaneous tirzepatide versus placebo added to titrated insulin glargine on glycemic control in patients with type 2 diabetes: the SURPASS-5 randomized clinical trial | 2022 | 486 | 16.96 | 15 | 99.85 | 2 | -13 | No | Yes |
| Drucker, Daniel J. (2020). Advances in oral peptide therapeutics | 2020 | 540 | 14.62 | 17 | 99.81 | 5 | -12 | No | Yes |

**Note:** *Rank difference was calculated as percentile-based rank minus mean-based rank. Positive values indicate a lower rank under the percentile-based method, whereas negative values indicate a higher rank.*

***Table S6. Top 25 references with the strongest citation bursts in GLP-1–based agonist research in T2DM, 2004–2025.***

| **No.** | **Title** | **Journal** | **Author** | **DOI** | **Year** | **Burst period** | **Strength** |
| --- | --- | --- | --- | --- | --- | --- | --- |
| 1 | Effects of exenatide (exendin-4) on glycemic control over 30 weeks in patients with type 2 diabetes treated with metformin and a sulfonylurea | Diabetes Care | Kendall, DM. et al. | 10.2337/diacare.28.5.1083 | 2005 | 2005–2010 | 39.67 |
| 2 | Effects of exenatide (exendin-4) on glycemic control and weight over 30 weeks in metformin-treated patients with type 2 diabetes | Diabetes Care | DeFronzo, RA. et al. | 10.2337/diacare.28.5.1092 | 2005 | 2005–2010 | 37.77 |
| 3 | Effects of exenatide (exendin-4) on glycemic control over 30 weeks in sulfonylurea-treated patients with type 2 diabetes | Diabetes Care | Buse, JB. et al. | 10.2337/diacare.27.11.2628 | 2004 | 2005–2009 | 33.75 |
| 4 | Efficacy and safety comparison of liraglutide, glimepiride, and placebo, all in combination with metformin, in type 2 diabetes: the LEAD (Liraglutide Effect and Action in Diabetes)-2 study | Diabetes Care | Nauck, M. et al. | 10.2337/dc08-1355 | 2009 | 2009–2014 | 46.87 |
| 5 | Liraglutide once a day versus exenatide twice a day for type 2 diabetes: a 26-week randomised, parallel-group, multinational, open-label trial (LEAD-6) | The Lancet | Buse, JB. et al. | 10.1016/S0140-6736(09)60659-0 | 2009 | 2009–2014 | 44.63 |
| 6 | Liraglutide versus glimepiride monotherapy for type 2 diabetes (LEAD-3 Mono): a randomised, 52-week, phase III, double-blind, parallel-treatment trial | The Lancet | Garber, A. et al. | 10.1016/S0140-6736(08)61246-5 | 2009 | 2009–2014 | 43.51 |
| 7 | Liraglutide, a once-daily human GLP-1 analogue, added to a sulphonylurea over 26 weeks produces greater improvements in glycaemic and weight control compared with adding rosiglitazone or placebo in subjects with type 2 diabetes (LEAD-1 SU) | Diabetic Medicine | Marre, M. et al. | 10.1111/j.1464-5491.2009.02666.x | 2009 | 2009–2014 | 32.87 |
| 8 | Exenatide once weekly versus twice daily for the treatment of type 2 diabetes: a randomised, open-label, non-inferiority study | The Lancet | Drucker, DJ. et al. | 10.1016/S0140-6736(08)61206-4 | 2008 | 2009–2013 | 31.24 |
| 9 | Medical management of hyperglycemia in type 2 diabetes: a consensus algorithm for the initiation and adjustment of therapy: a consensus statement of the American Diabetes Association and the European Association for the Study of Diabetes | Diabetes Care | Nathan, DM. et al. | 10.2337/dc08-9025 | 2009 | 2009–2014 | 31.19 |
| 10 | Management of hyperglycemia in type 2 diabetes: a patient-centered approach: position statement of the American Diabetes Association (ADA) and the European Association for the Study of Diabetes (EASD) | Diabetes Care | Inzucchi, SE. et al. | 10.2337/dc12-0413 | 2012 | 2012–2017 | 41.71 |
| 11 | Management of hyperglycemia in type 2 diabetes, 2015: a patient-centered approach: update to a position statement of the American Diabetes Association and the European Association for the Study of Diabetes | Diabetes Care | Inzucchi, SE. et al. | 10.2337/dc14-2441 | 2015 | 2015–2019 | 60.72 |
| 12 | Lixisenatide in patients with type 2 diabetes and acute coronary syndrome | New England Journal of Medicine | Pfeffer, MA. et al. | 10.1056/NEJMoa1509225 | 2015 | 2016–2020 | 36.99 |
| 13 | Liraglutide and cardiovascular outcomes in type 2 diabetes | New England Journal of Medicine | Marso, SP. et al. | 10.1056/NEJMoa1603827 | 2016 | 2017–2021 | 150.65 |
| 14 | Canagliflozin and cardiovascular and renal events in type 2 diabetes | New England Journal of Medicine | Neal, B. et al. | 10.1056/NEJMoa1611925 | 2017 | 2018–2022 | 48.02 |
| 15 | Effects of once-weekly exenatide on cardiovascular outcomes in type 2 diabetes | New England Journal of Medicine | Holman, RR. et al. | 10.1056/NEJMoa1612917 | 2017 | 2018–2022 | 38.13 |
| 16 | Management of hyperglycemia in type 2 diabetes, 2018: a consensus report by the American Diabetes Association (ADA) and the European Association for the Study of Diabetes (EASD) | Diabetes Care | Davies, MJ. et al. | 10.2337/dci18-0033 | 2018 | 2019–2022 | 84.19 |
| 17 | Albiglutide and cardiovascular outcomes in patients with type 2 diabetes and cardiovascular disease (Harmony Outcomes): a double-blind, randomised placebo-controlled trial | The Lancet | Hernandez, AF. et al. | 10.1016/S0140-6736(18)32261-X | 2018 | 2019–2023 | 32.98 |
| 18 | Semaglutide versus dulaglutide once weekly in patients with type 2 diabetes (SUSTAIN 7): a randomised, open-label, phase 3b trial | The Lancet Diabetes & Endocrinology | Pratley, RE. et al. | 10.1016/S2213-8587(18)30024-X | 2018 | 2019–2023 | 31.12 |
| 19 | Dulaglutide and cardiovascular outcomes in type 2 diabetes (REWIND): a double-blind, randomised placebo-controlled trial | The Lancet | Gerstein, HC. et al. | 10.1016/S0140-6736(19)31149-3 | 2019 | 2020–2025 | 66.40 |
| 20 | Cardiovascular, mortality, and kidney outcomes with GLP-1 receptor agonists in patients with type 2 diabetes: a systematic review and meta-analysis of cardiovascular outcome trials | The Lancet Diabetes & Endocrinology | Kristensen, SL. et al. | 10.1016/S2213-8587(19)30249-9 | 2019 | 2020–2025 | 34.08 |
| 21 | Management of hyperglycemia in type 2 diabetes, 2022: a consensus report by the American Diabetes Association (ADA) and the European Association for the Study of Diabetes (EASD) | Diabetes Care | Davies, MJ. et al. | 10.2337/dci22-0034 | 2022 | 2023–2025 | 49.90 |
| 22 | Cardiovascular, mortality, and kidney outcomes with GLP-1 receptor agonists in patients with type 2 diabetes: a systematic review and meta-analysis of randomised trials | The Lancet Diabetes & Endocrinology | Sattar, N. et al. | 10.1016/S2213-8587(21)00203-5 | 2021 | 2023–2025 | 39.72 |
| 23 | Tirzepatide versus semaglutide once weekly in patients with type 2 diabetes | New England Journal of Medicine | Frías, JP. et al. | 10.1056/NEJMoa2107519 | 2021 | 2023–2025 | 39.43 |
| 24 | GLP-1 receptor agonists in the treatment of type 2 diabetes—state-of-the-art | Molecular Metabolism | Nauck, MA. et al. | 10.1016/j.molmet.2020.101102 | 2020 | 2023–2025 | 33.44 |
| 25 | 9. Pharmacologic approaches to glycemic treatment: Standards of Care in Diabetes—2023 | Diabetes Care | ElSayed, NA. et al. | 10.2337/dc23-S009 | 2023 | 2023–2025 | 32.92 |

**Note:** *References are ordered primarily by burst onset year to show temporal evolution; “No.” is an item identifier, not a rank by burst strength. Burst period denotes the interval of the citation burst, and strength denotes its intensity.*
